# Supplementary figures and images for: Genome biology and evolution of mating-type loci in four cereal rust fungi
Source: PLoS Genet. 2024 Mar 18;20(3):e1011207. doi: 10.1371/journal.pgen.1011207 (PMC10977897; doi:10.1371/journal.pgen.1011207)

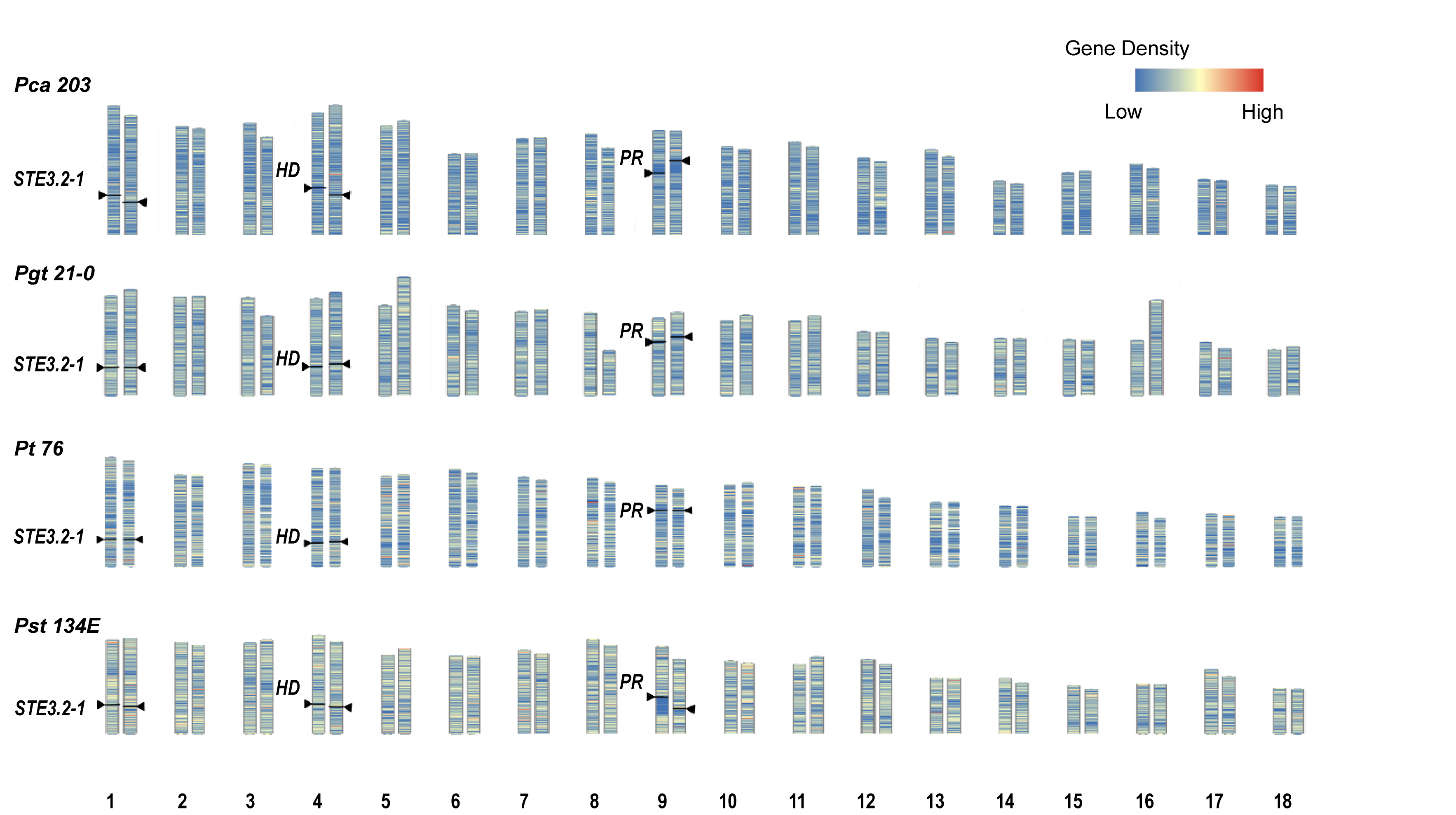

Supplement: S1 Fig — Karyograms of P. coronata f. sp. avenae (“Pca 203”), P. graminis f. sp. tritici (“Pgt 21–0”), P. triticina (“Pt 76”) and P. striiformis f. sp. tritici (“Pst 134E”) with the positions of HD, PR and STE3.2–1 loci marked by black arrow heads. HD, PR and STE3.2–1, located on chromosome 4, chromosome 9 and chromosome 1, respectively, suggest tetrapolar mating types in these four species. (TIF) [file pgen.1011207.s003.tif]

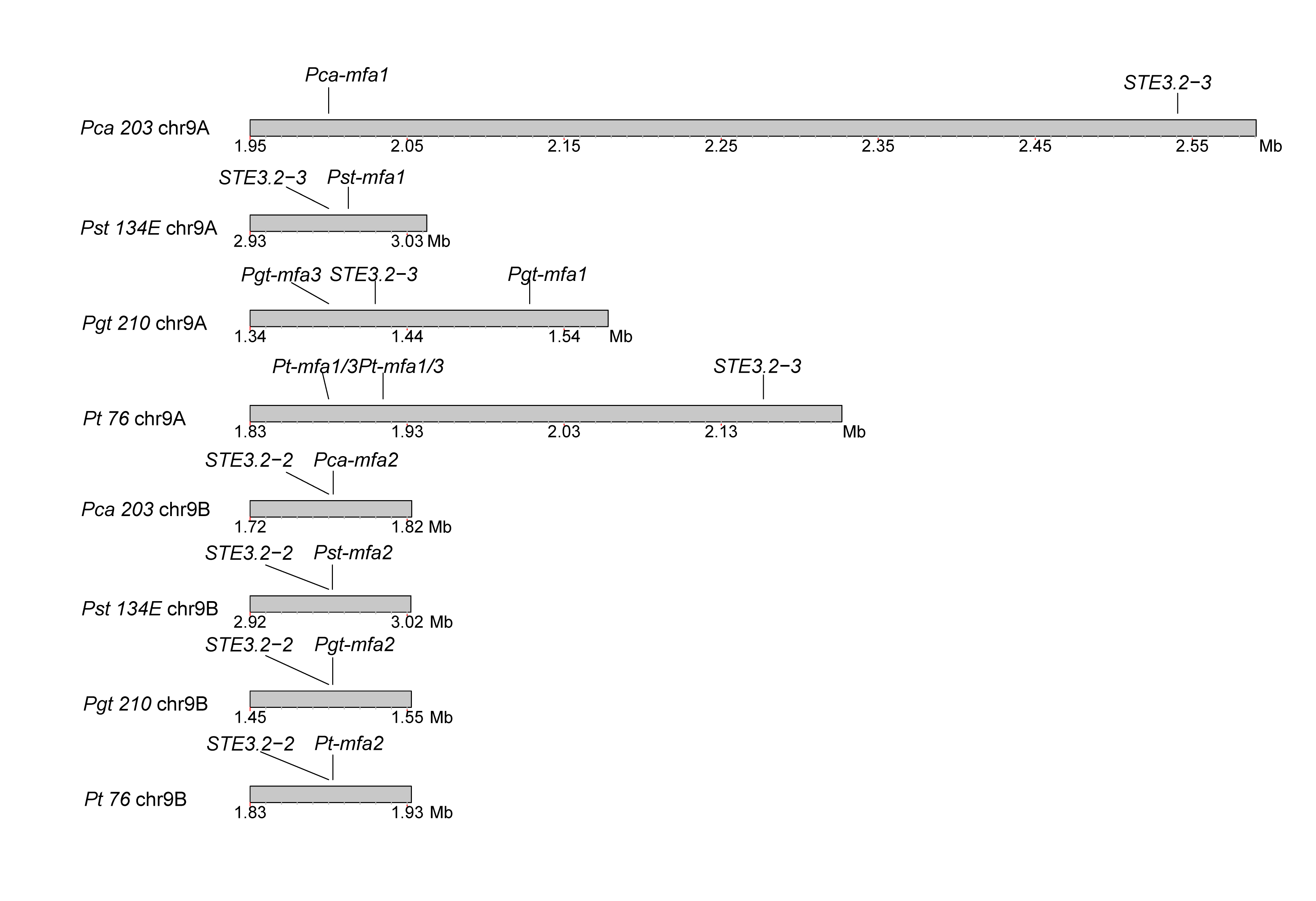

Supplement: S2 Fig — The diagrams display the location of STE3.2–2 and STE3.2–3 and their linked mfa genes on chromosome 9A and 9B in P. coronata f. sp. avenae (“Pca 203”), P. graminis f. sp. tritici (“Pgt 21–0”), P. triticina (“Pt 76”) and P. striiformis f. sp. tritici (“Pst 134E”). The grey bar represents chromosome subsections containing the genes of interest with the numbers indicating the absolute location in mega base pairs on chromosome 9A and 9B. The genetic distance between mfa1/mfa3 and STE3.2–3 are highly variable between species, whereas STE3.2–2 and mfa2 are tightly linked in all species. (TIF) [file pgen.1011207.s004.tif]

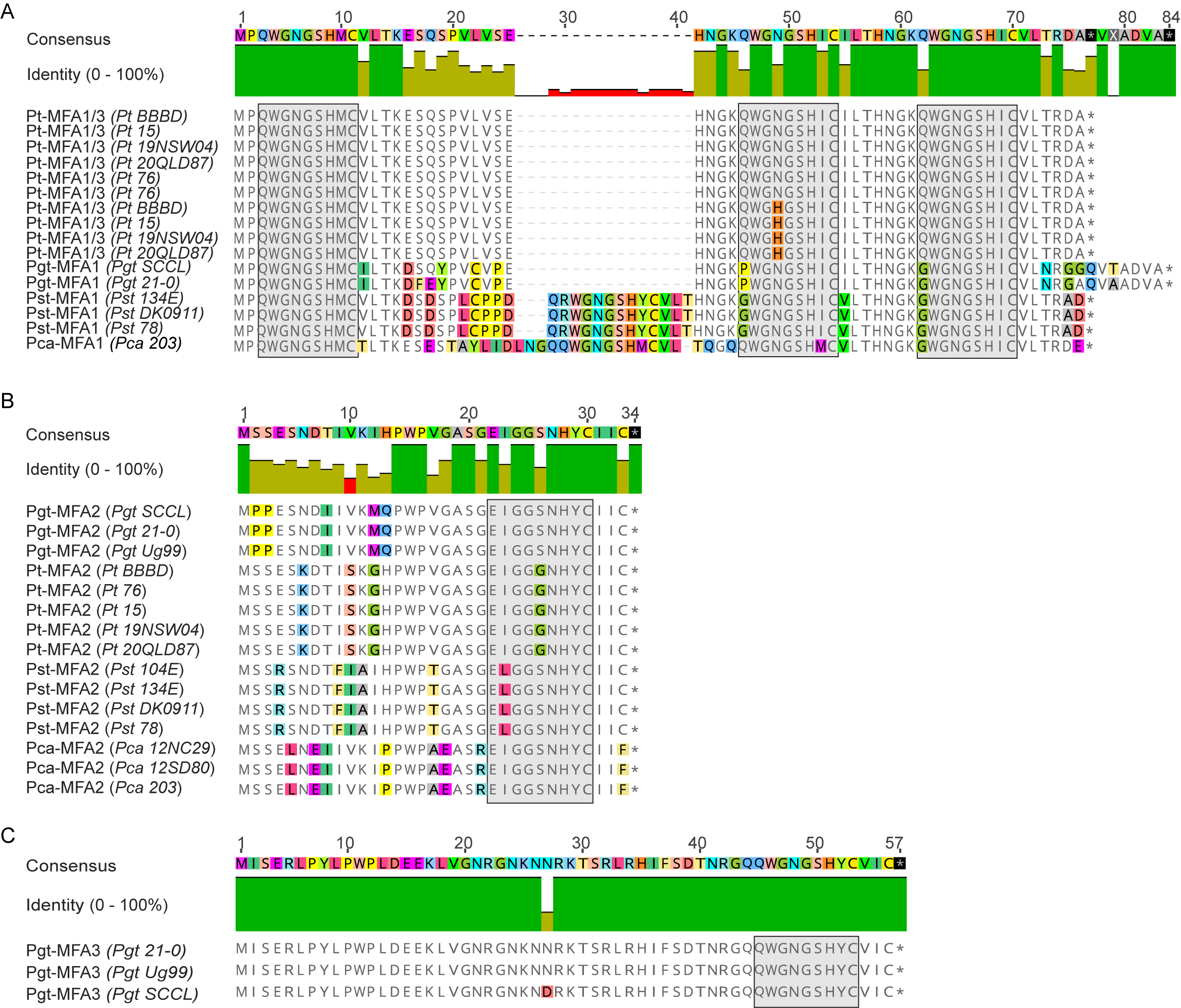

Supplement: S3 Fig — (A) Alignment of MFA1 protein sequences from four different rust fungal species. The MFA1, which is linked to STE3.2–3, is mostly conserved within species. Two near identical MFA1/3 copies can be identified in four P. triticina isolates. In P. graminis f. sp. tritici MFA1 has four amino acid substitutions in Pgt SCCL versus Pgt 21–0. (B) Alignment of MFA2 protein sequences from four different rust fungal species. MFA2 is fully conserved within each species. (C) Alignment of MFA3 protein sequences from three P. graminis f. sp. tritici isolates. Mfa3 is only present P. graminis f. sp. tritici in close proximity to STE3.2–3. Predicted mature pheromone sequences are outlined by boxes. (TIF) [file pgen.1011207.s005.tif]

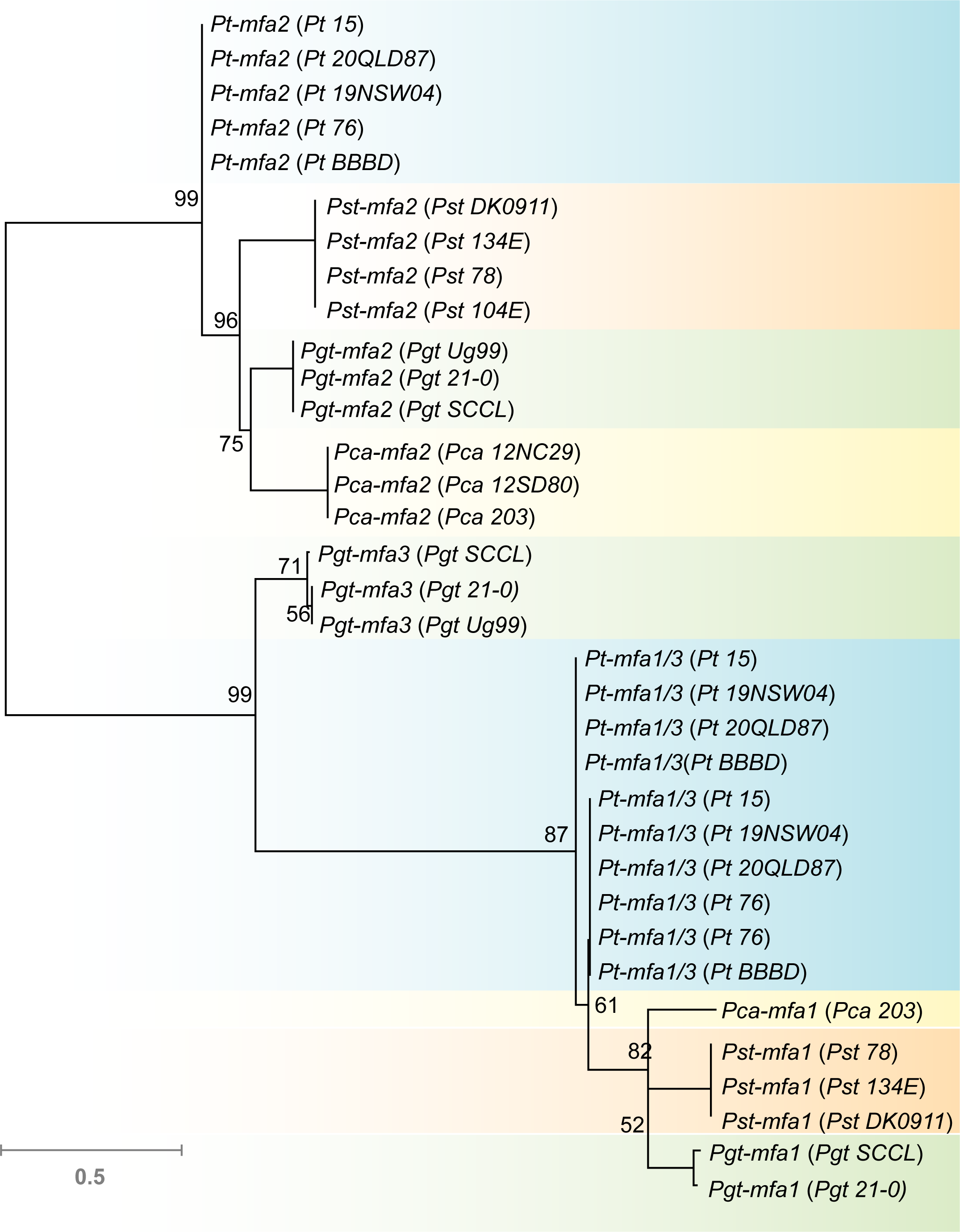

Supplement: S4 Fig — Maximum likelihood tree of mfas identified in four cereal rust fungi including multiple isolates per species. Tips are labelled with the species abbreviation, gene names, and isolate names are provided in parentheses. Branch support was assessed by 10000 replicates. The scale bar represents 0.5 substitutions per site. Pca—P. coronata f. sp. avenae, Pgt—Puccinia graminis f. sp. tritici, Pt—P. triticina and Pst—P. striiformis f. sp. tritici. (TIF) [file pgen.1011207.s006.tif]

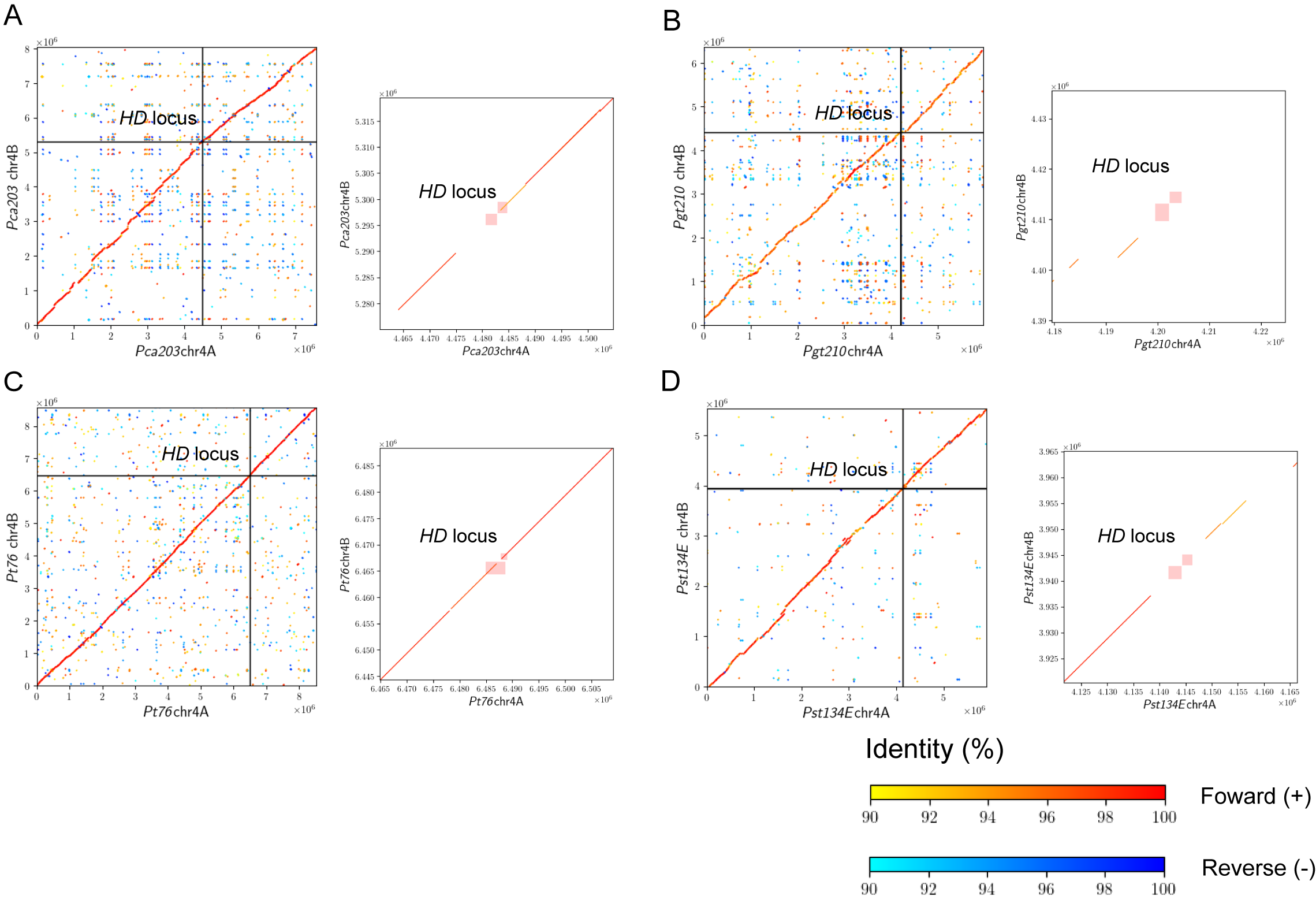

Supplement: S5 Fig — The figure shows dots plots of whole chromosome alignments between the two HD loci containing chromosomes from dikaryotic genome assemblies. Each panel consists of dot plots of the whole chromosome and subset dot plot zooming into the HD locus. The HD locus is labelled and line colors show the nucleotide percentage identity and nucleotide orientation as indicated in the figure legend. Subfigure A to D show P. coronata f. sp. avenae (“Pca 203”), P. graminis f. sp. tritici (“Pgt 21–0”), P. triticina (“Pt 76”) and P. striiformis f. sp. tritici (“Pst 134E”), respectively. (TIF) [file pgen.1011207.s007.tif]

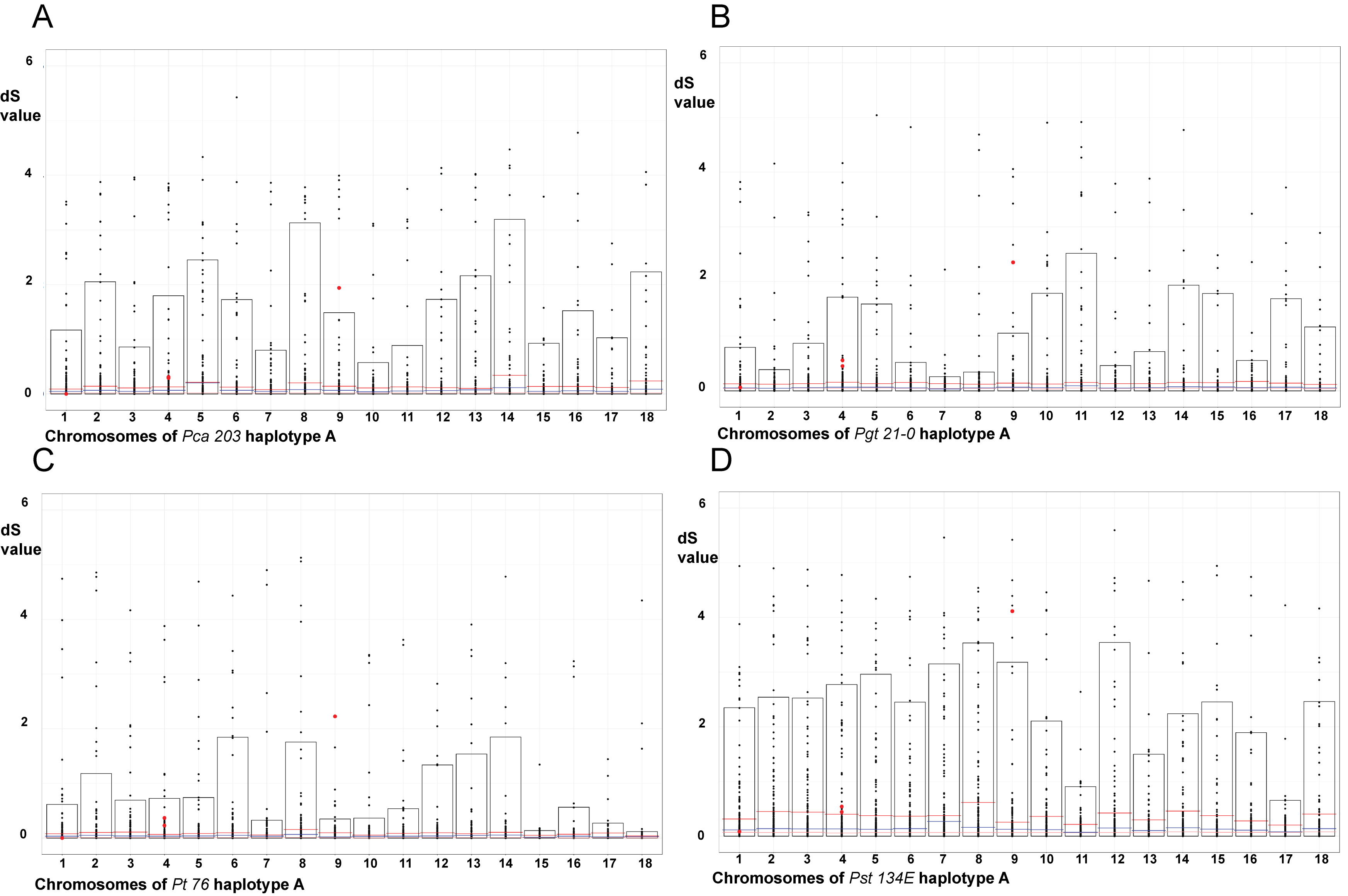

Supplement: S6 Fig — The plots show the distribution of dS values of allele pairs on sister chromosomes in four cereal rust fungal species. Bar plots show the distribution of dS values up to the 99% quantile. Red lines represent threshold of dS values of 95% of all alleles. Blue lines represent threshold of dS values of 90% of all alleles. Black points show dS values of individual allele pairs. HD and STE3 allele pairs are highlighted as red points. Subfigures A to D show P. coronata f. sp. avenae (“Pca 203”), P. graminis f. sp. tritici (“Pgt 21–0”), P. triticina (“Pt 76”) and P. striiformis f. sp. tritici (“Pst 134E”), respectively. (TIF) [file pgen.1011207.s008.tif]

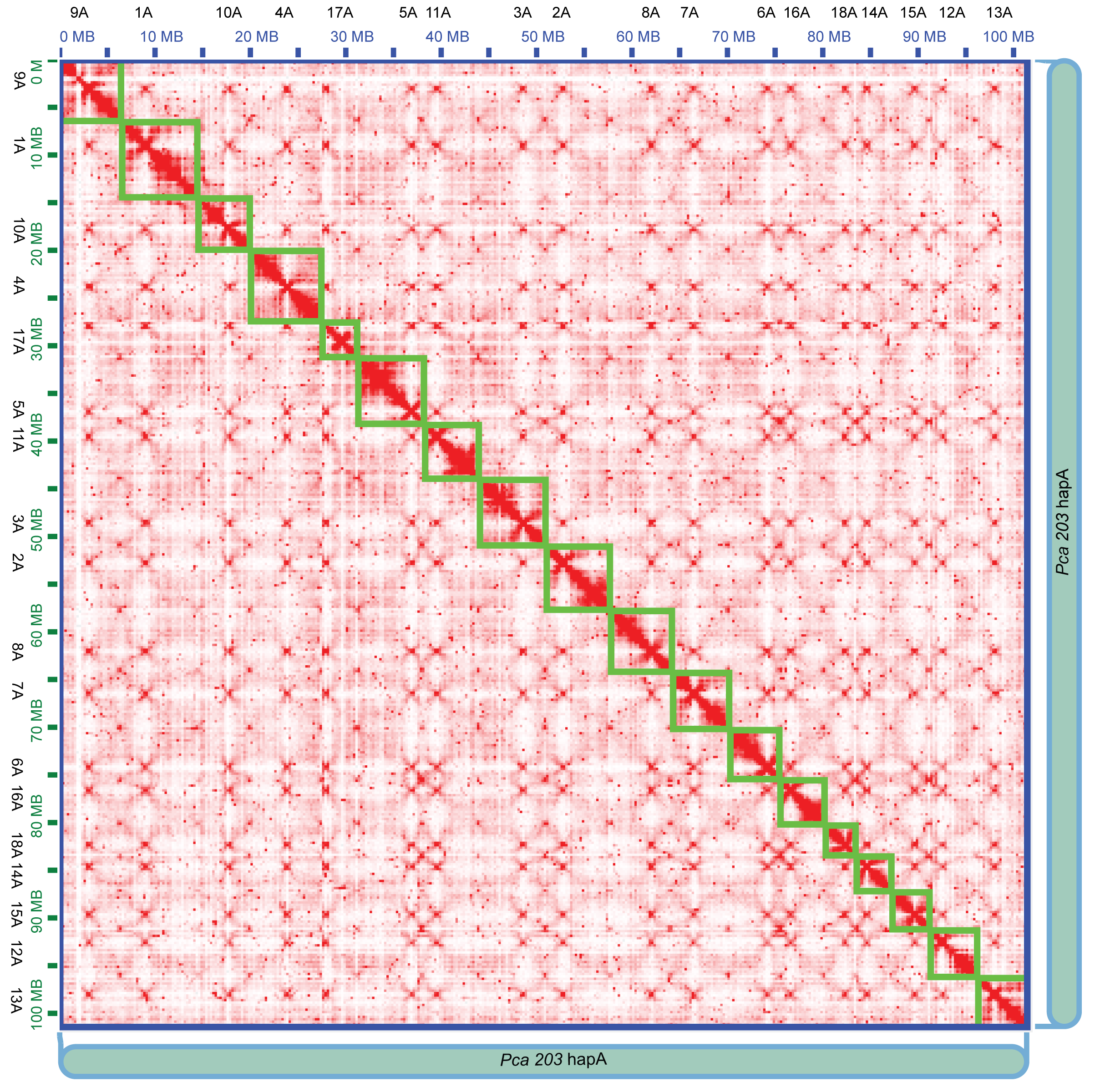

Supplement: S7 Fig — (TIF) [file pgen.1011207.s009.tif]

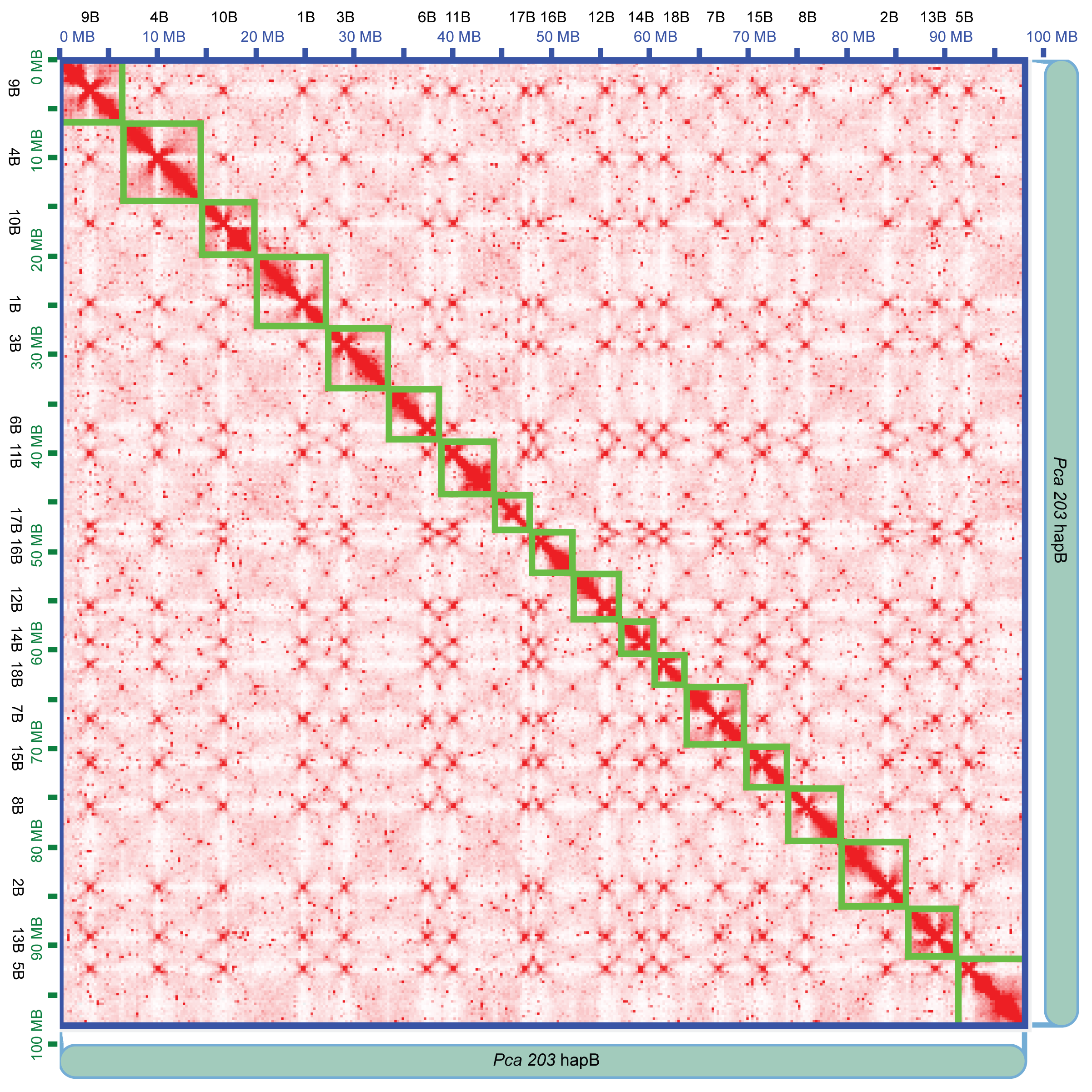

Supplement: S8 Fig — (TIF) [file pgen.1011207.s010.tif]

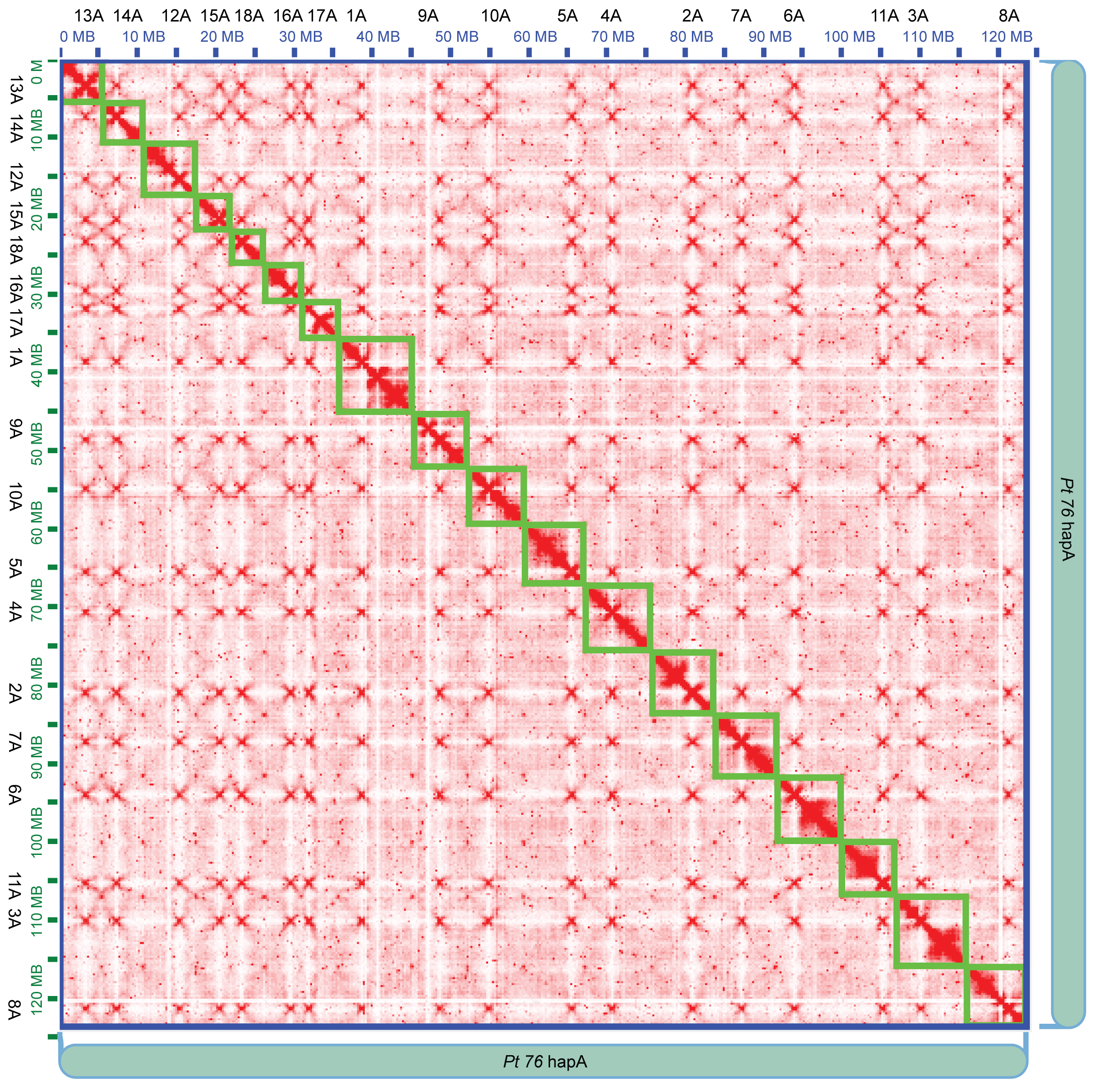

Supplement: S9 Fig — (TIF) [file pgen.1011207.s011.tif]

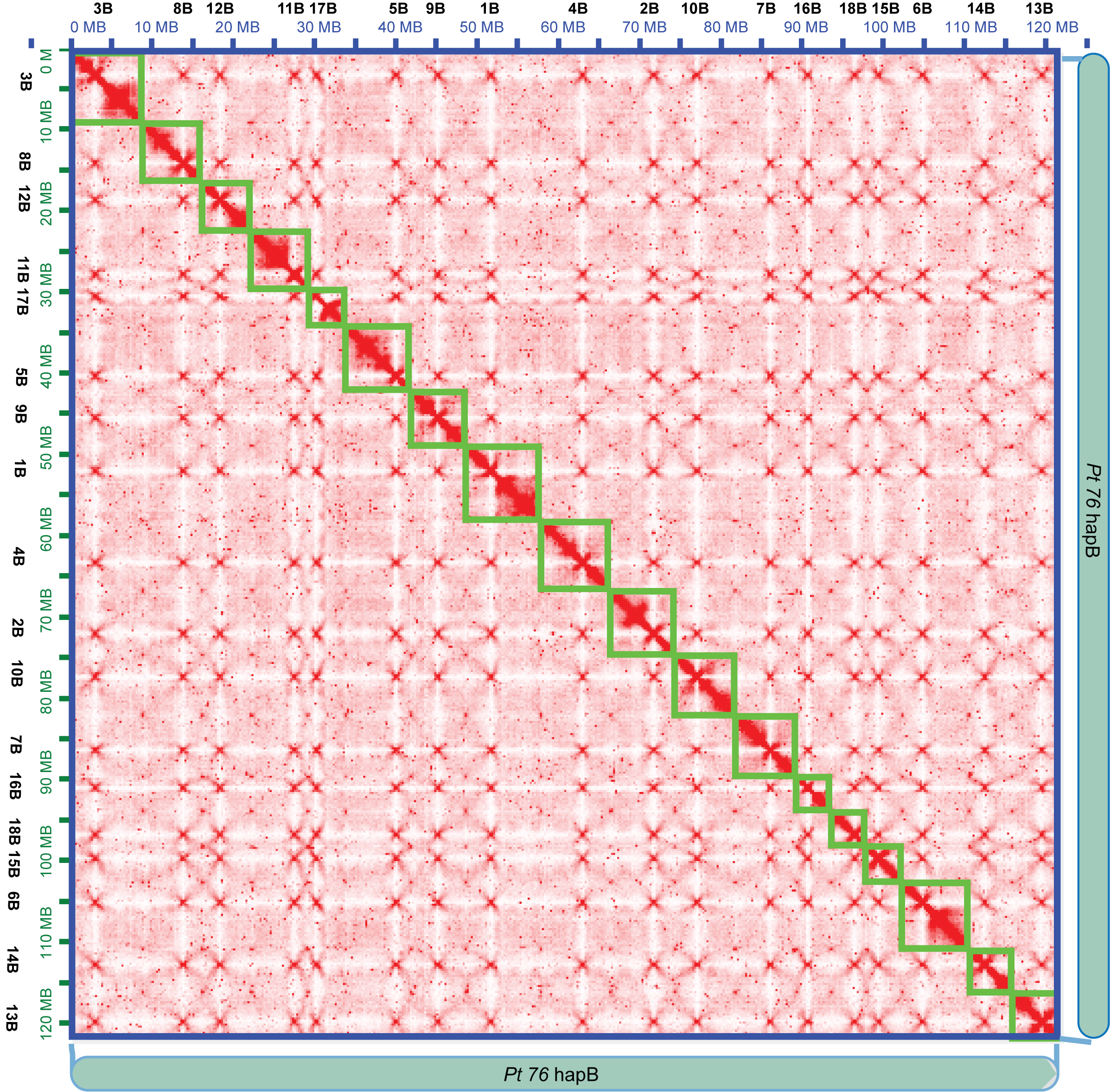

Supplement: S10 Fig — (TIF) [file pgen.1011207.s012.tif]

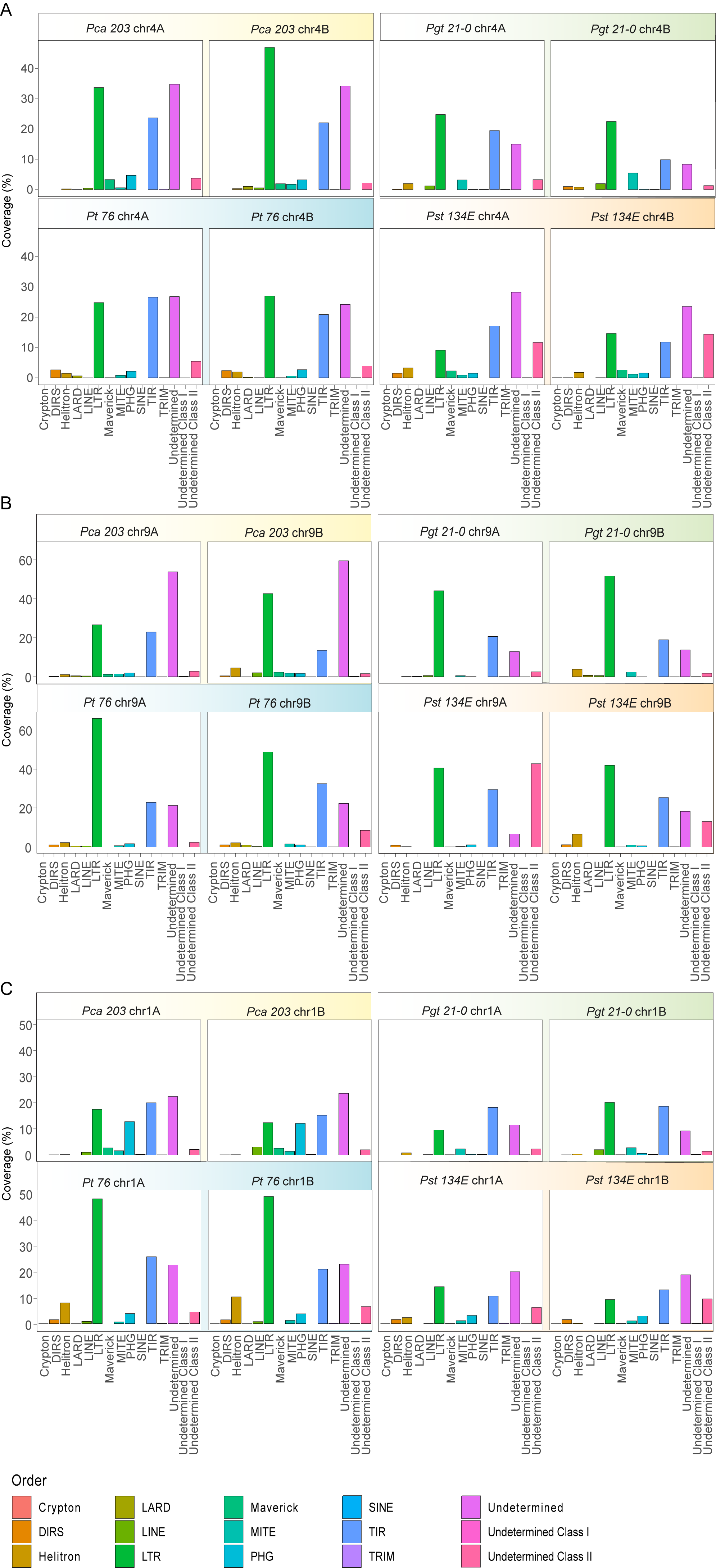

Supplement: S11 Fig — The plots show the percentage of nucleotides covered by different transposable element orders at (A) HD locus (B) PR locus (C) STE3.2–1 locus. Each subfigure A to C shows the coverage in each haplotype of the dikaryotic genomes of P. coronata f. sp. avenae (“Pca 203”), P. graminis f. sp. tritici (“Pgt 21–0”), P. triticina (“Pt 76”) and P. striiformis f. sp. tritici (“Pst 134E”). Different TE orders are color coded as shown in the legend. TEs with no assigned class are labelled “Undetermined”. TEs with no assigned order but belonging to Class I (RNA retrotransposons) or Class II (DNA transposons) are labelled “Undetermined Class I” or “Undetermined Class II”, respectively. (TIF) [file pgen.1011207.s013.tif]

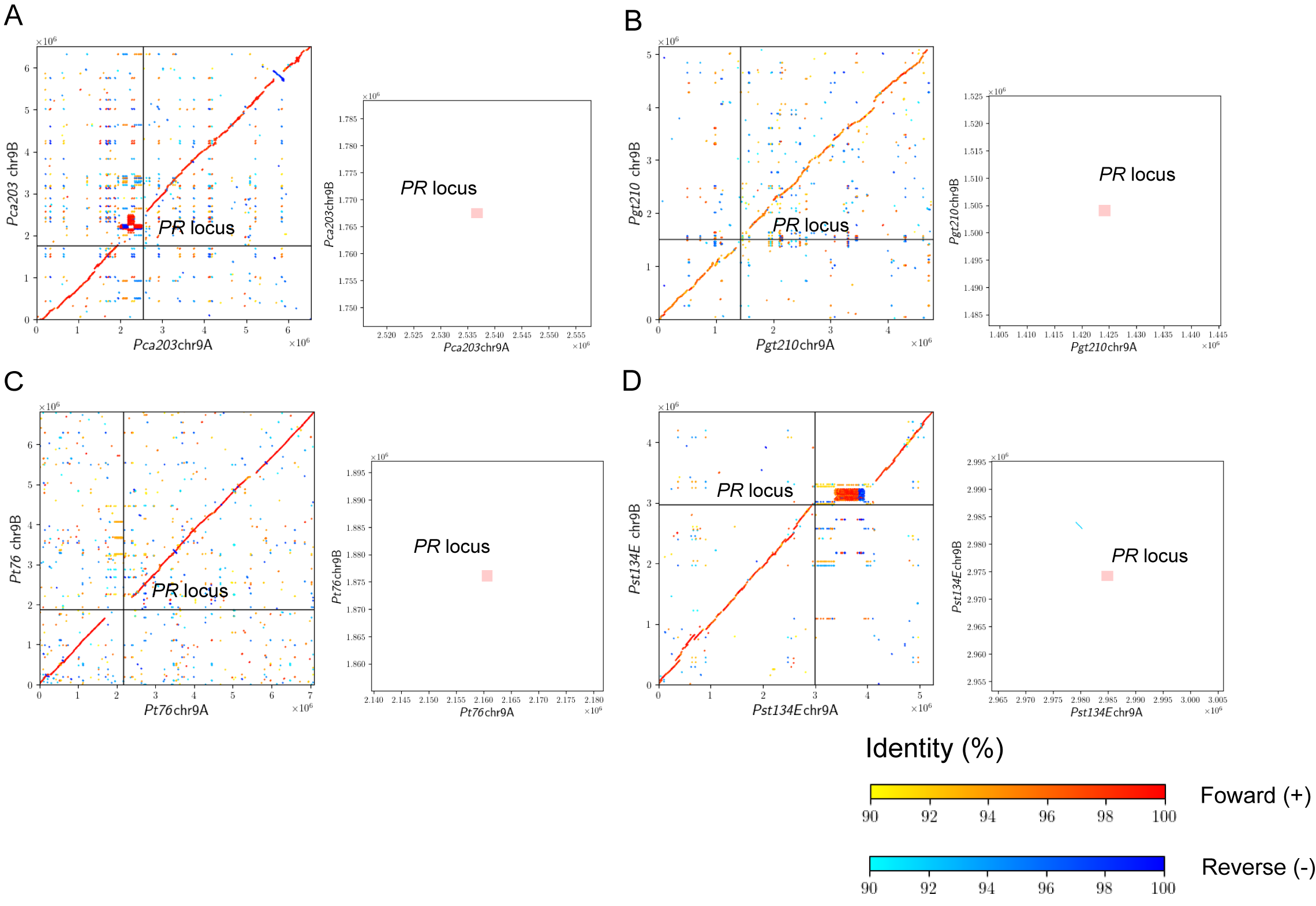

Supplement: S12 Fig — The figure shows dot plots of whole chromosome alignments between the two PR loci containing chromosomes from the dikaryotic genome assemblies. Each panel consists of dot plots of the whole chromosome and subset dot plots zooming into the PR locus. The PR locus is labelled and line colors show the nucleotide percentage identity and nucleotide orientation as indicated in the figure legend. Subfigures A to D show P. coronata f. sp. avenae (“Pca 203”), P. graminis f. sp. tritici (“Pgt 21–0”), P. triticina (“Pt 76”) and P. striiformis f. sp. tritici (“Pst 134E”), respectively. (TIF) [file pgen.1011207.s014.tif]

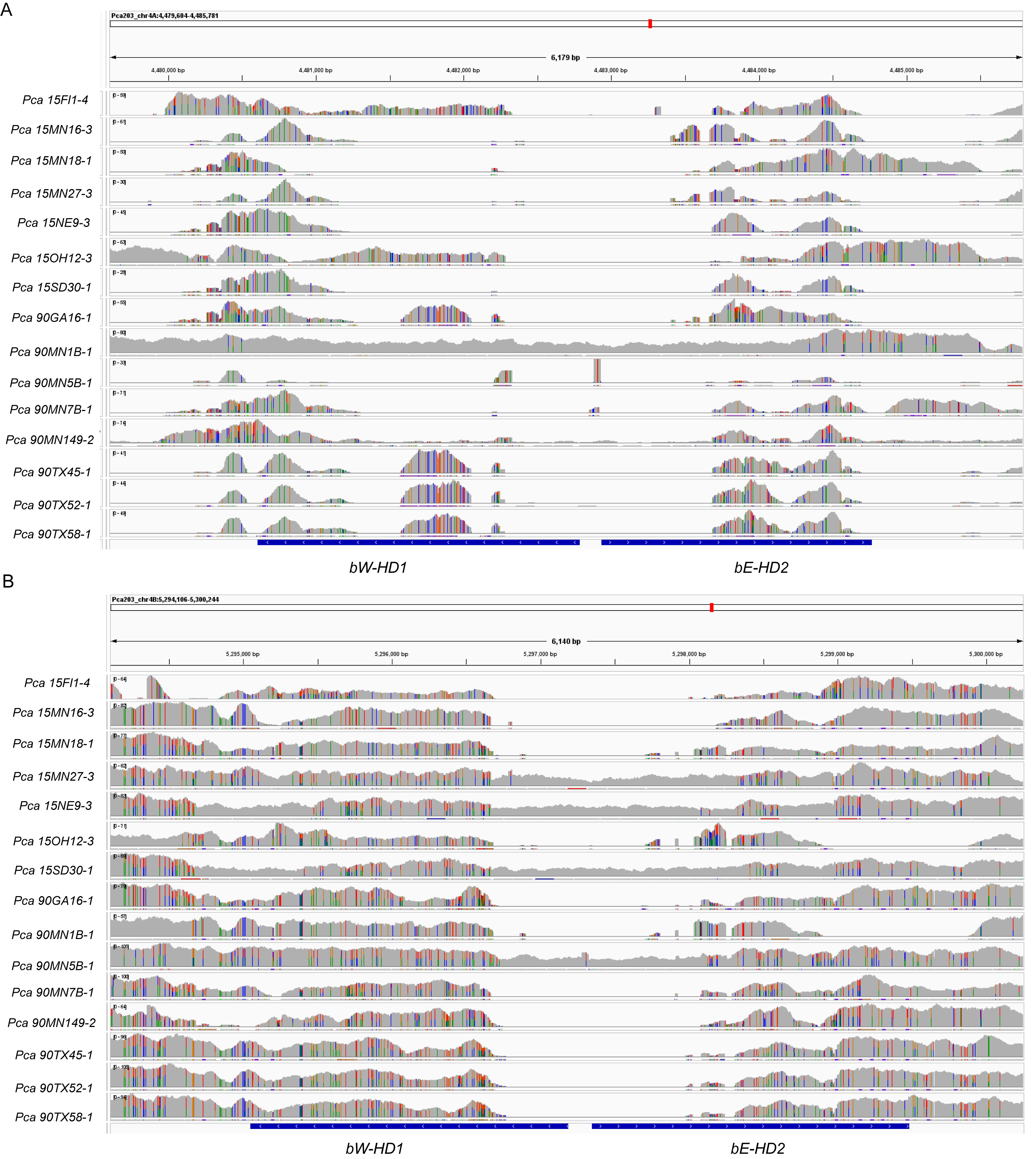

Supplement: S13 Fig — (A) shows mapping against the HD locus on chromosome 4A and (B) chromosome 4B, respectively. (TIF) [file pgen.1011207.s015.tif]

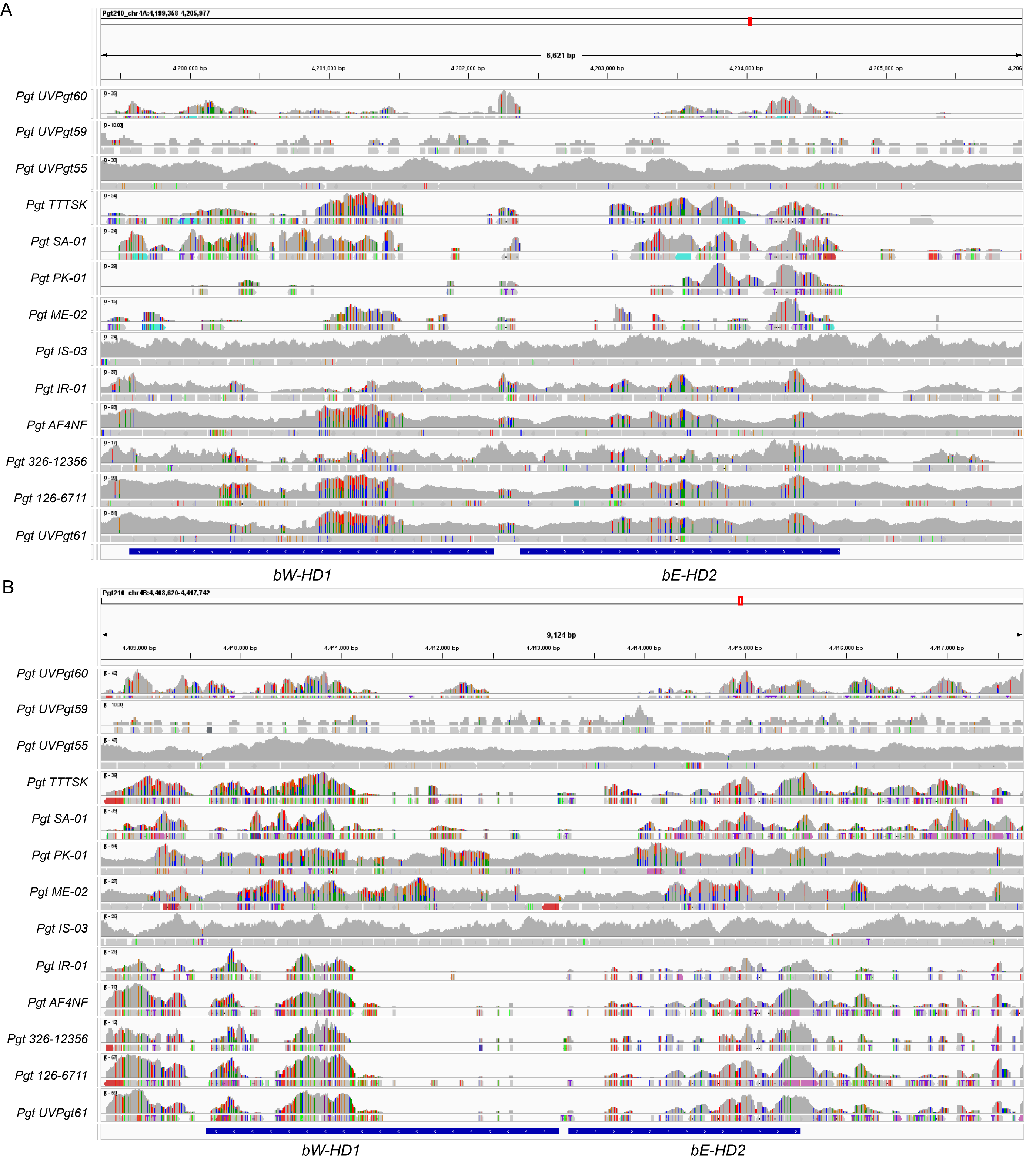

Supplement: S14 Fig — (A) shows mapping against the HD locus on chromosome 4A and (B) chromosome 4B, respectively. (TIF) [file pgen.1011207.s016.tif]

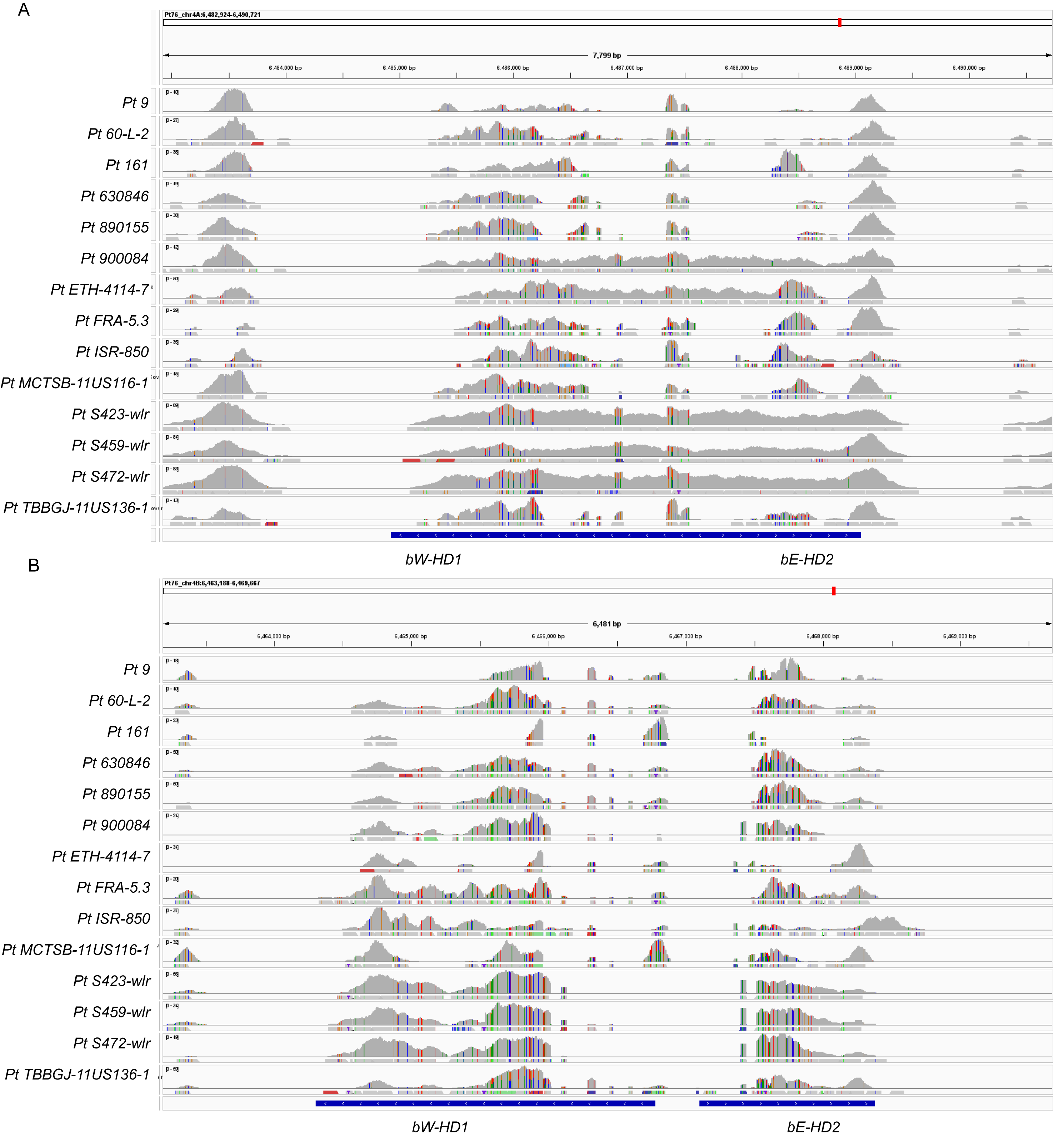

Supplement: S15 Fig — (A) shows mapping against the HD locus on chromosome 4A and (B) chromosome 4B, respectively. (TIF) [file pgen.1011207.s017.tif]

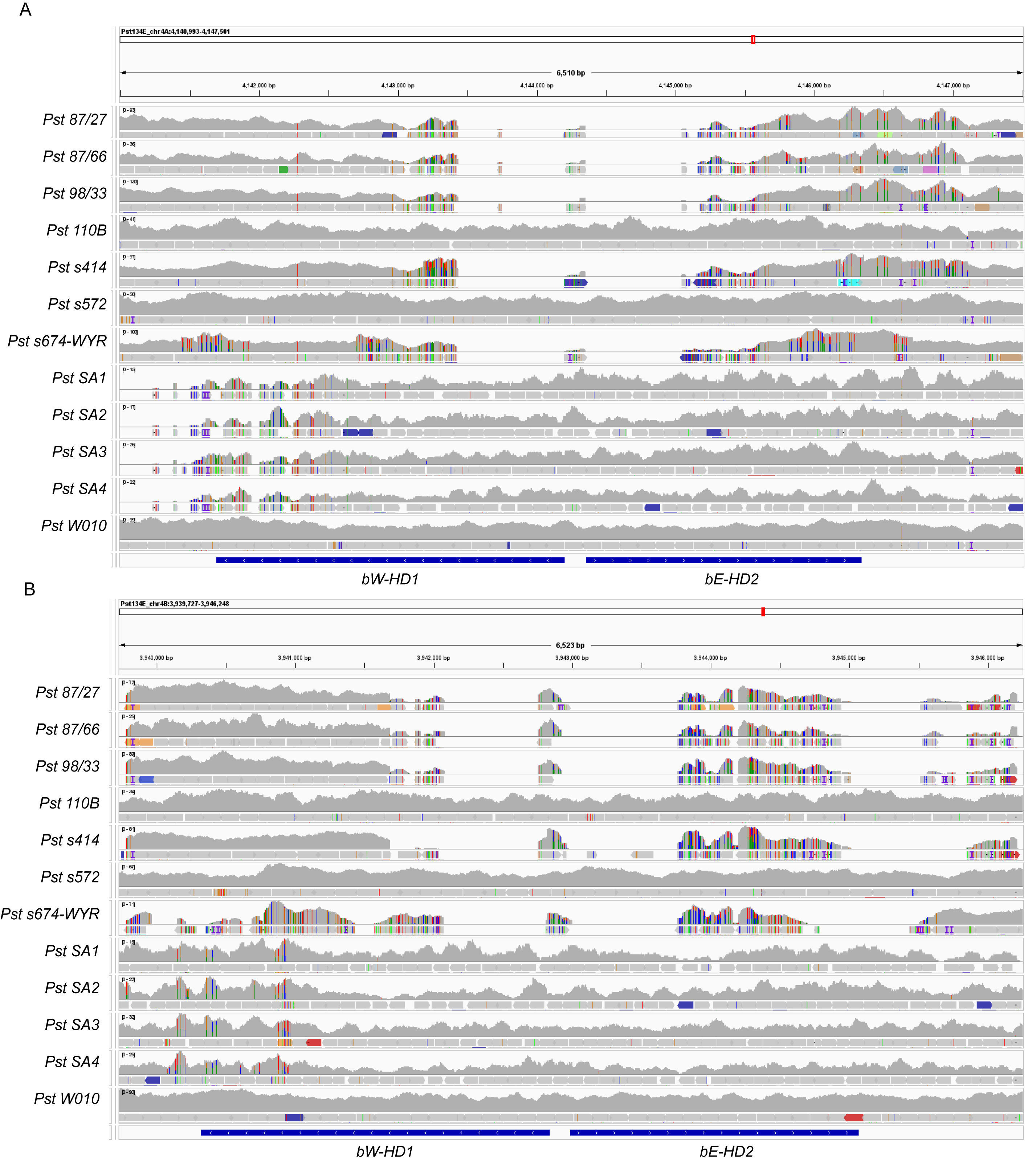

Supplement: S16 Fig — (A) shows mapping against the HD locus on chromosome 4A and (B) chromosome 4B, respectively. (TIF) [file pgen.1011207.s018.tif]

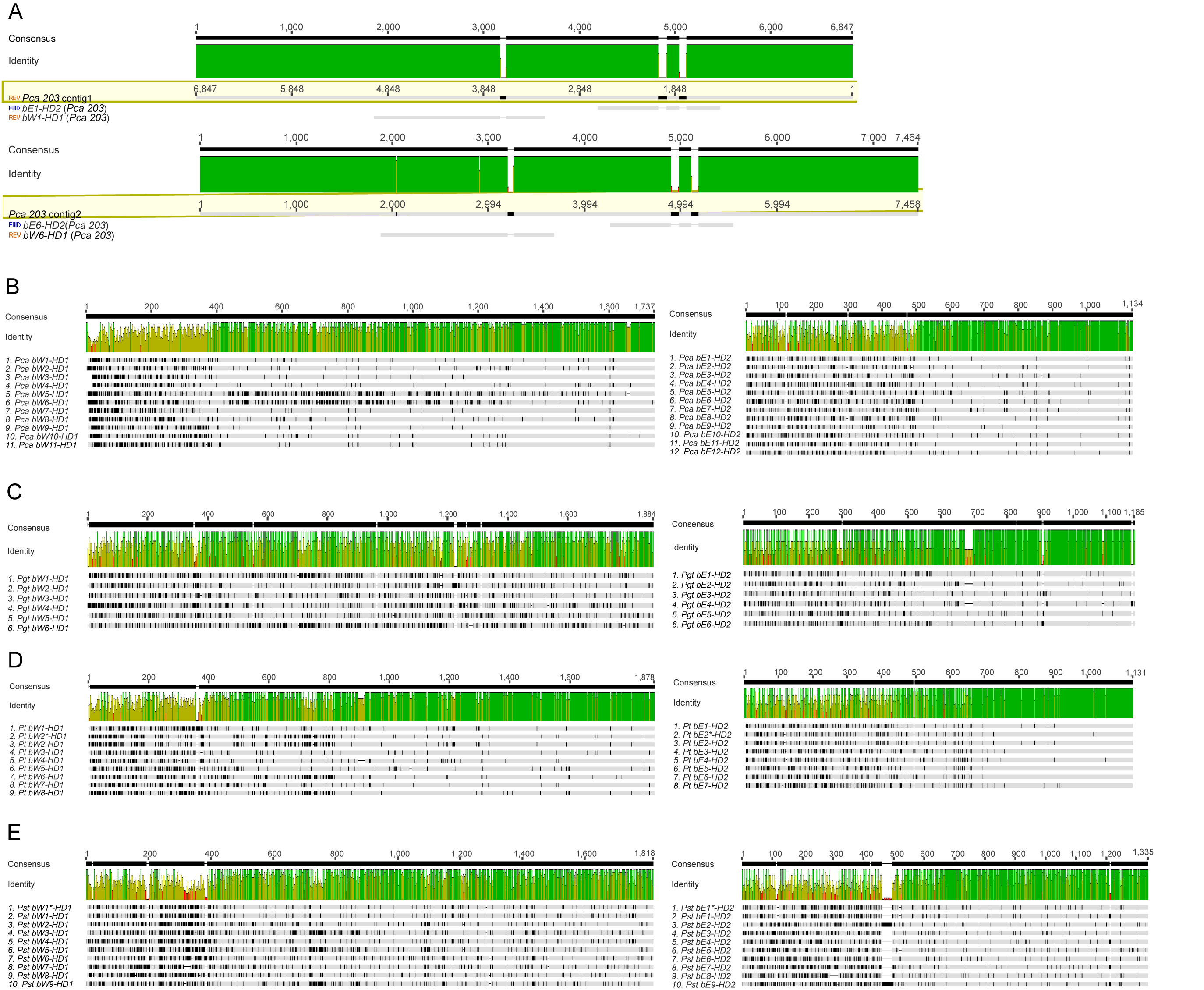

Supplement: S17 Fig — (A) Nucleotide alignment of the de novo reconstructed HD locus from Pca 203 Illumina short-read data with the coding regions of bW-HD1 and bE-HD2 alleles from Pca 203 dikaryotic reference genome. (B) to (D) multiple sequence alignments of de novo reconstructed HD coding regions, (E) multiple sequence alignments of Pst bW-HD1 and bE-HD2 alleles [159]. In each subfigure B to E, the top two track shows the consensus sequence length and relative sequence identity, respectively. Subfigure B to E show P. coronata f. sp. avenae (“Pca”), P. graminis f. sp. tritici (“Pgt”), P. triticina (“Pt”) and P. striiformis f. sp. tritici (“Pst”), respectively. The bW-HD1 and bE-HD2 are numbered in accordance with Fig 1. (TIF) [file pgen.1011207.s019.tif]

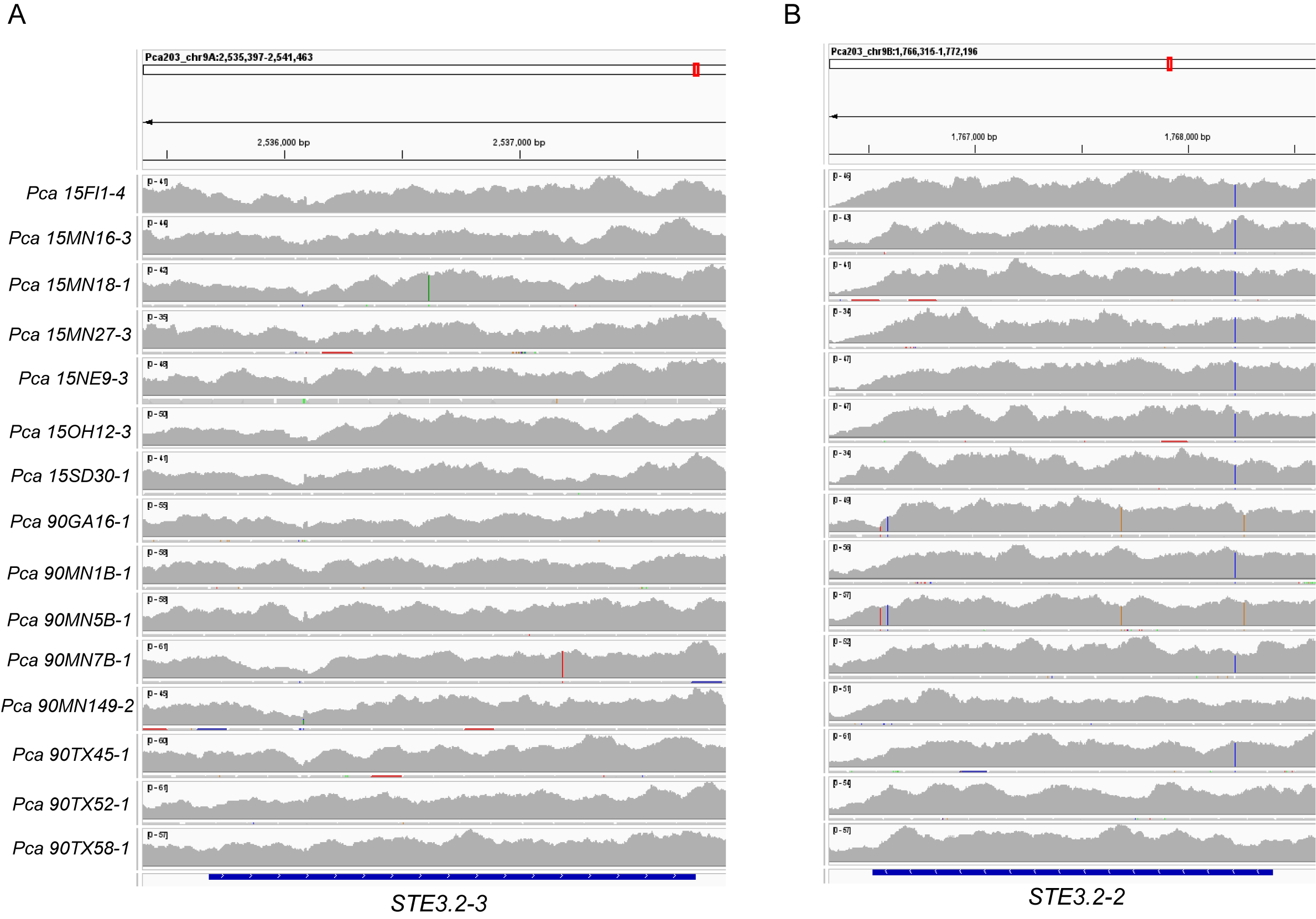

Supplement: S18 Fig — (A) shows mapping against the PR locus on chromosome 9A and (B) chromosome 9B, respectively. (TIF) [file pgen.1011207.s020.tif]

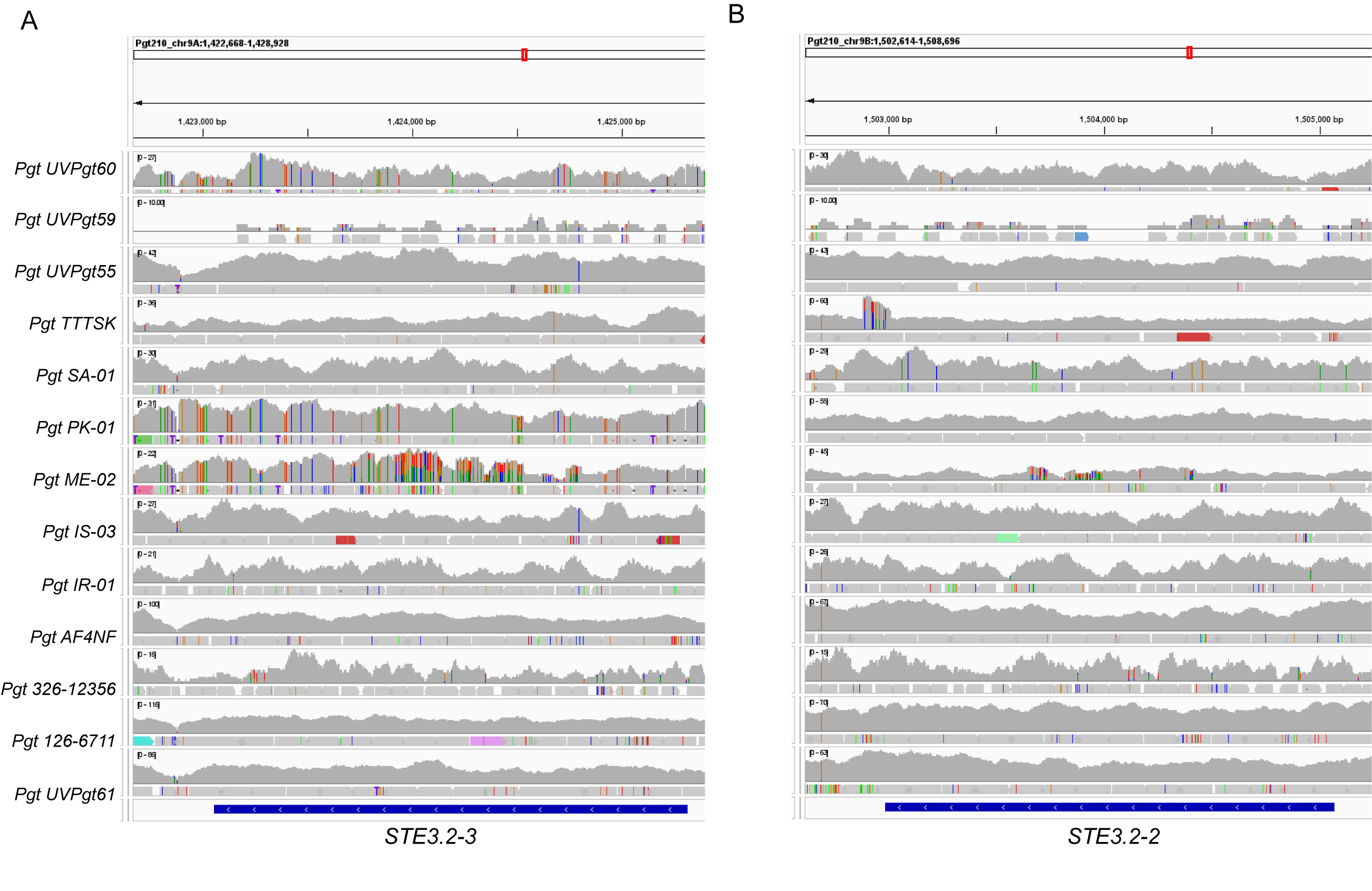

Supplement: S19 Fig — (A) shows mapping against the PR locus on chromosome 9A and (B) chromosome 9B, respectively. (TIF) [file pgen.1011207.s021.tif]

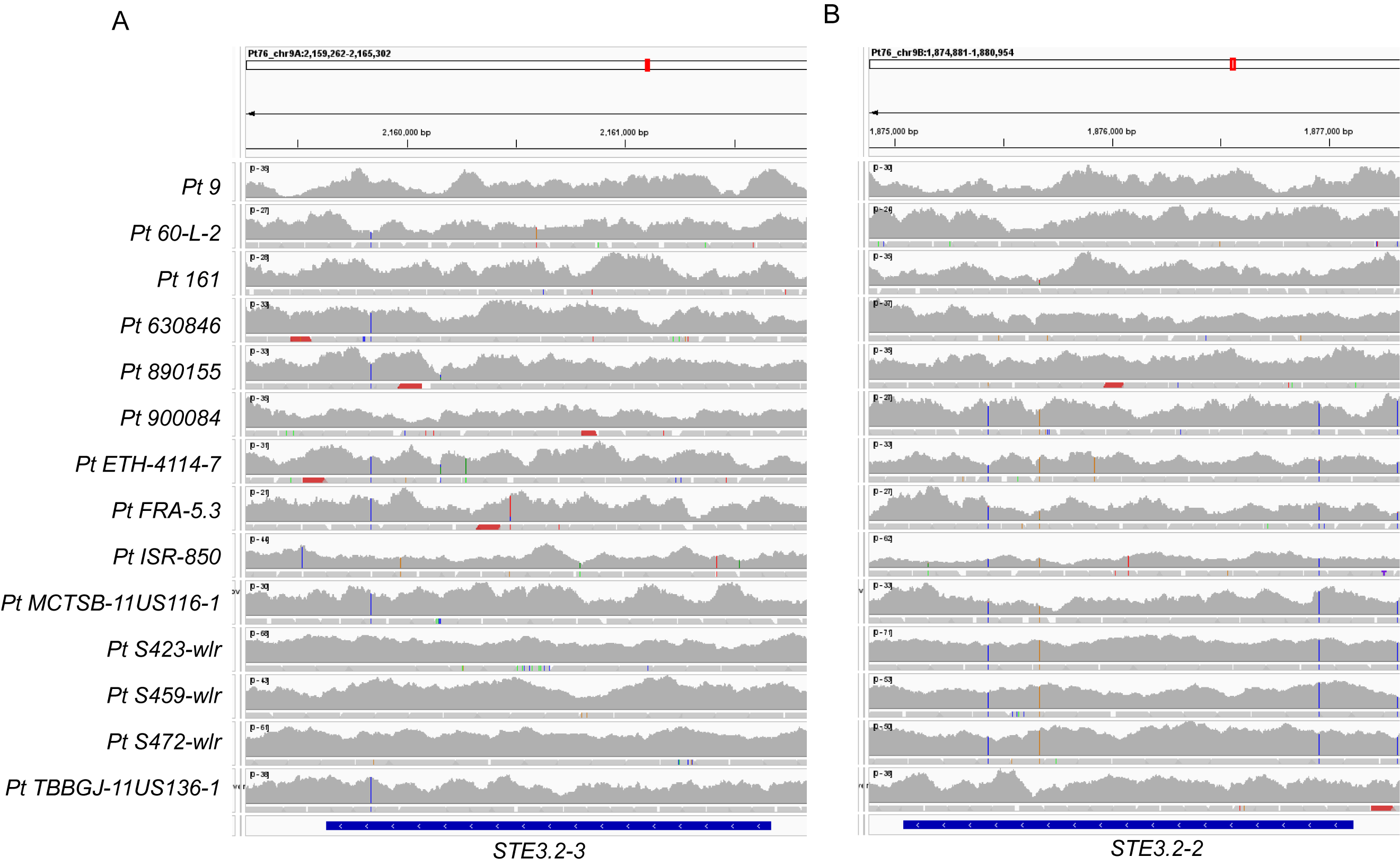

Supplement: S20 Fig — (A) shows mapping against the PR locus on chromosome 9A and (B) chromosome 9B, respectively. (TIF) [file pgen.1011207.s022.tif]

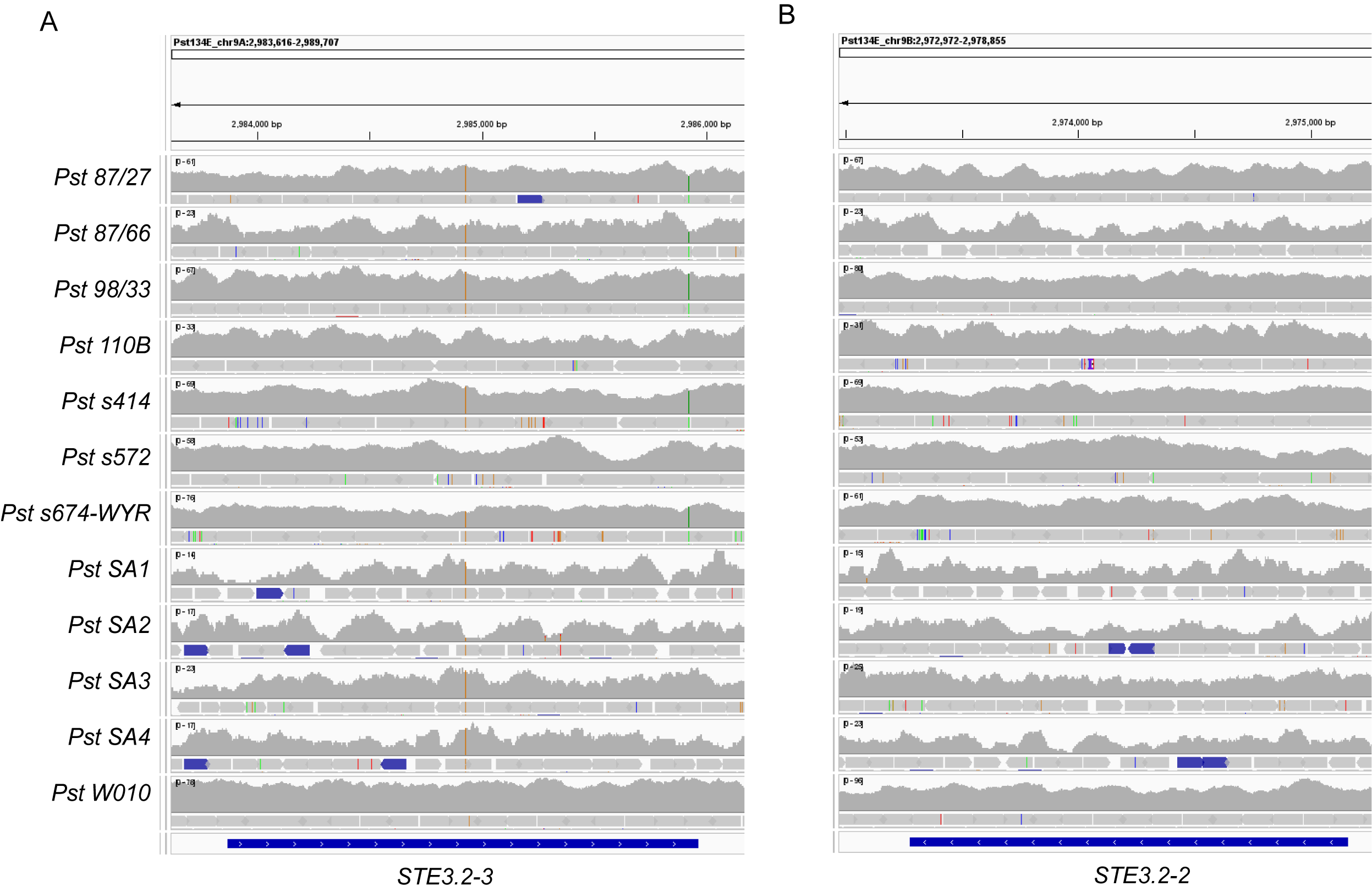

Supplement: S21 Fig — (A) shows mapping against the PR locus on chromosome 9A and (B) chromosome 9B, respectively. (TIF) [file pgen.1011207.s023.tif]

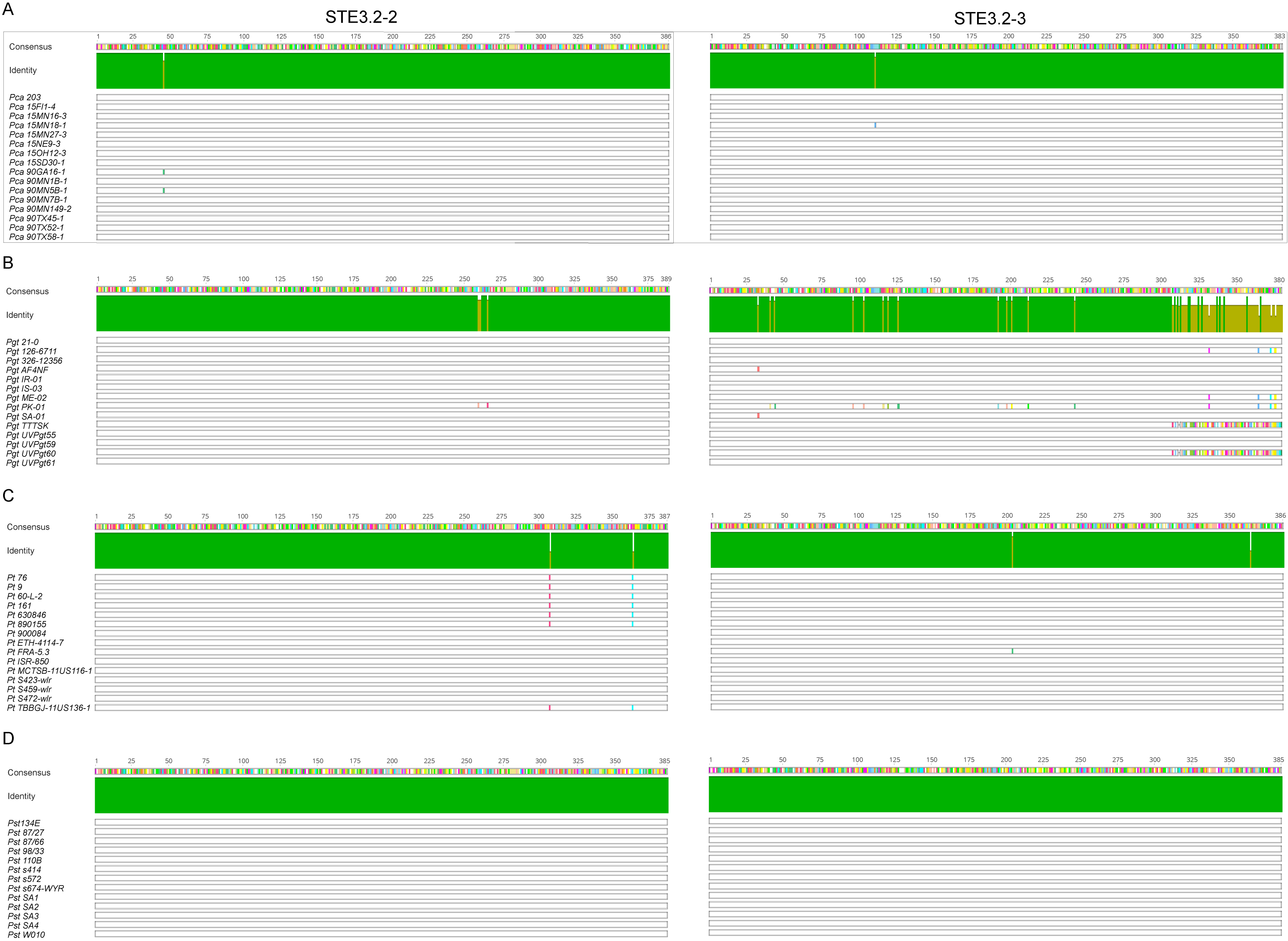

Supplement: S22 Fig — Multiple sequence alignment of de novo reconstructed STE3.2–2 and STE3.2–3 protein sequences. Subfigure contains MSAs; one for STE3.2–2 and one for STE3.2–3. Subfigure A to D show P. coronata f. sp. avenae (“Pca”), P. graminis f. sp. tritici (“Pgt”), P. triticina (“Pt”) and P. striiformis f. sp. tritici (“Pst”), respectively. (TIF) [file pgen.1011207.s024.tif]

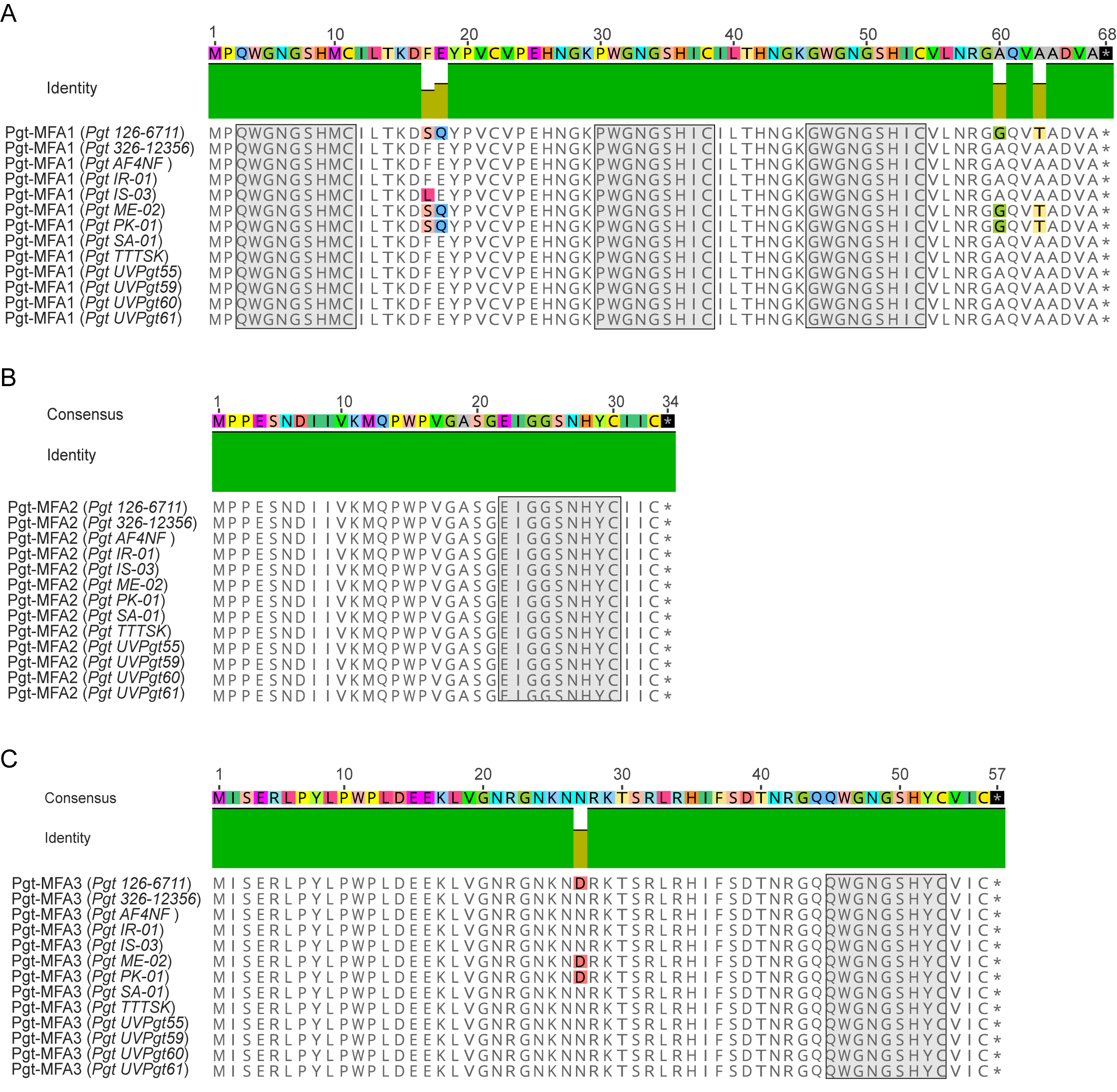

Supplement: S23 Fig — Amino acid substitutions are highlighted by color, whereas predicted mature pheromone sequences are outlined by boxes. (TIF) [file pgen.1011207.s025.tif]

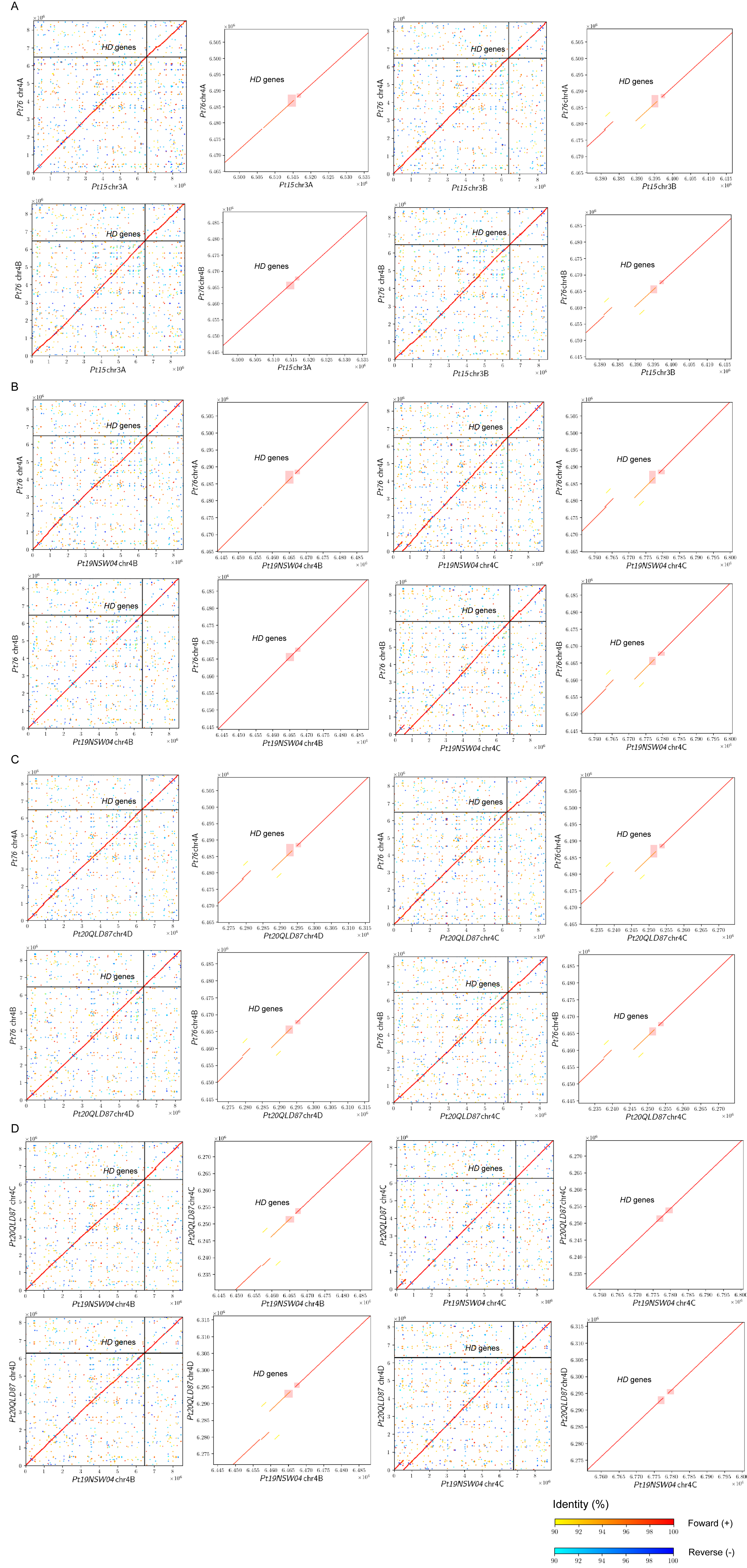

Supplement: S24 Fig — The figure shows dots plots of whole chromosome alignments of HD loci containing chromosomes derived from distinct dikaryotic genomes of four different P. triticina isolates including Pt 15, Pt 19NSW04, Pt 20QLD87 against Pt 76. Each panel consists of a dot plot of the whole chromosome and a subset dot plot zooming into the HD locus. The HD locus is labelled and line colors show the nucleotide percentage identity and nucleotide orientation as indicated in the figure legend. (A) Comparison of nucleotide sequence of chromosome 4s of Pt 15 and Pt 76. (B) Comparison of nucleotide sequence of chromosome 4s of Pt 19NSW04 and Pt 76. (C) Comparison of nucleotide sequence of chromosome 4s of Pt 20QLD87 and Pt 76. (D) Comparison of nucleotide sequence of chromosome 4s of Pt 19NSW04 and Pt 20QLD87. (TIF) [file pgen.1011207.s026.tif]

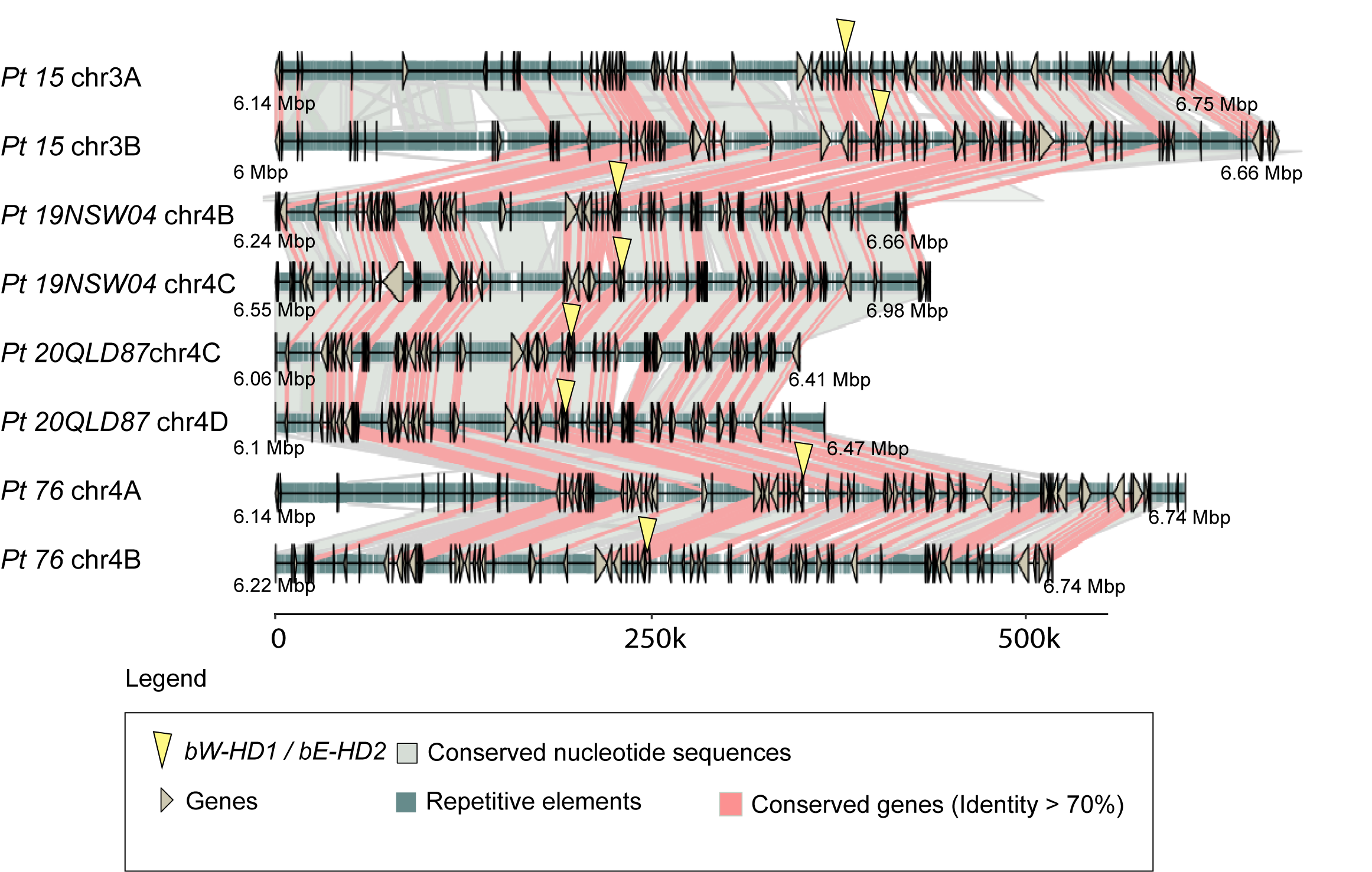

Supplement: S25 Fig — Synteny graphs of HD loci including proximal regions in the four P. triticina isolates Pt 15, Pt 19NSW04, Pt 20QLD87 and Pt 76. Red lines between chromosome sections represent gene pairs with nucleotide sequence identity higher than 70% and grey shades between conserved nucleotide sequences (> = 1000 bp and identity > = 90%). For additional annotations please refer to the provide legend (“Legend”). (TIF) [file pgen.1011207.s027.tif]

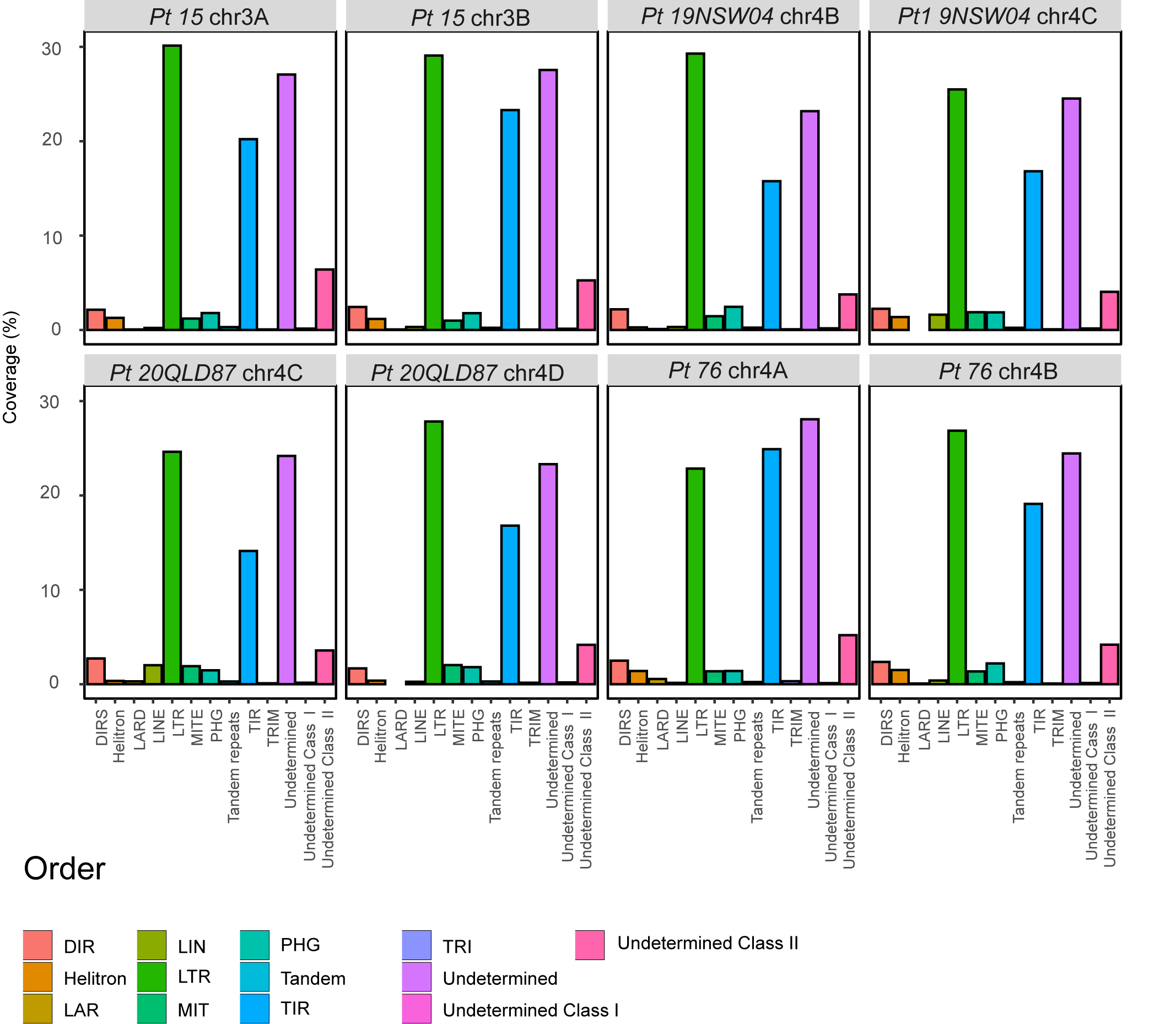

Supplement: S26 Fig — The plots show the percentage of nucleotides covered by different transposable element orders at the HD locus. Each subfigure shows the coverage in each haplotype of the dikaryotic genomes of P. triticina isolates Pt 15, Pt 19NSW04, Pt 20QLD87 and Pt 76. Different TE orders are color coded as shown in the legend. TEs with no assigned class are labelled “Undetermined”. TEs with no assigned order but belonging to Class I (RNA retrotransposons) or Class II (DNA transposons) are labelled “Undetermined Class I” or “Undetermined Class II”, respectively. (TIF) [file pgen.1011207.s028.tif]

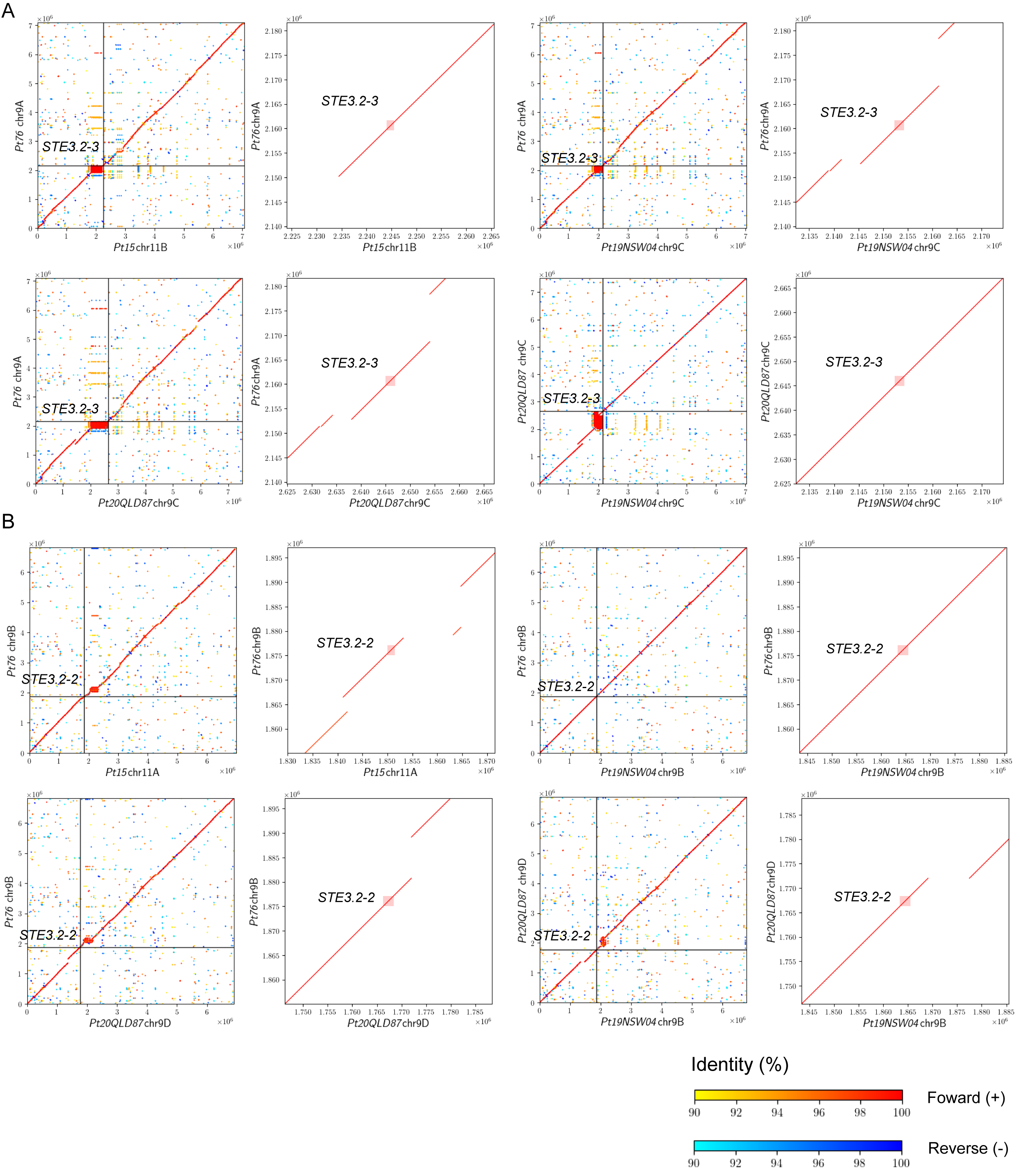

Supplement: S27 Fig — The figure shows dot plots of whole chromosome alignments of STE3.2–2 or STE3.2–3 containing chromosomes derived from distinct dikaryotic genomes of four different P. triticina isolates including Pt 15, Pt 19NSW04, Pt 20QLD87 against Pt 76. Each panel consists of a dot plot of the whole chromosome and a subset dot plot zooming into the proximal region of STE3.2–2 or STE3.2–3. The STE3.2–2 or STE3.2- are labelled and line colors show the nucleotide percentage identity and nucleotide orientation as indicated in the figure legend. (A) Comparison of nucleotide sequence of chromosome 9s containing STE3.2–2 gene (B) Comparison of nucleotide sequence of chromosome 9s containing STE3.2–3 gene. (TIF) [file pgen.1011207.s029.tif]

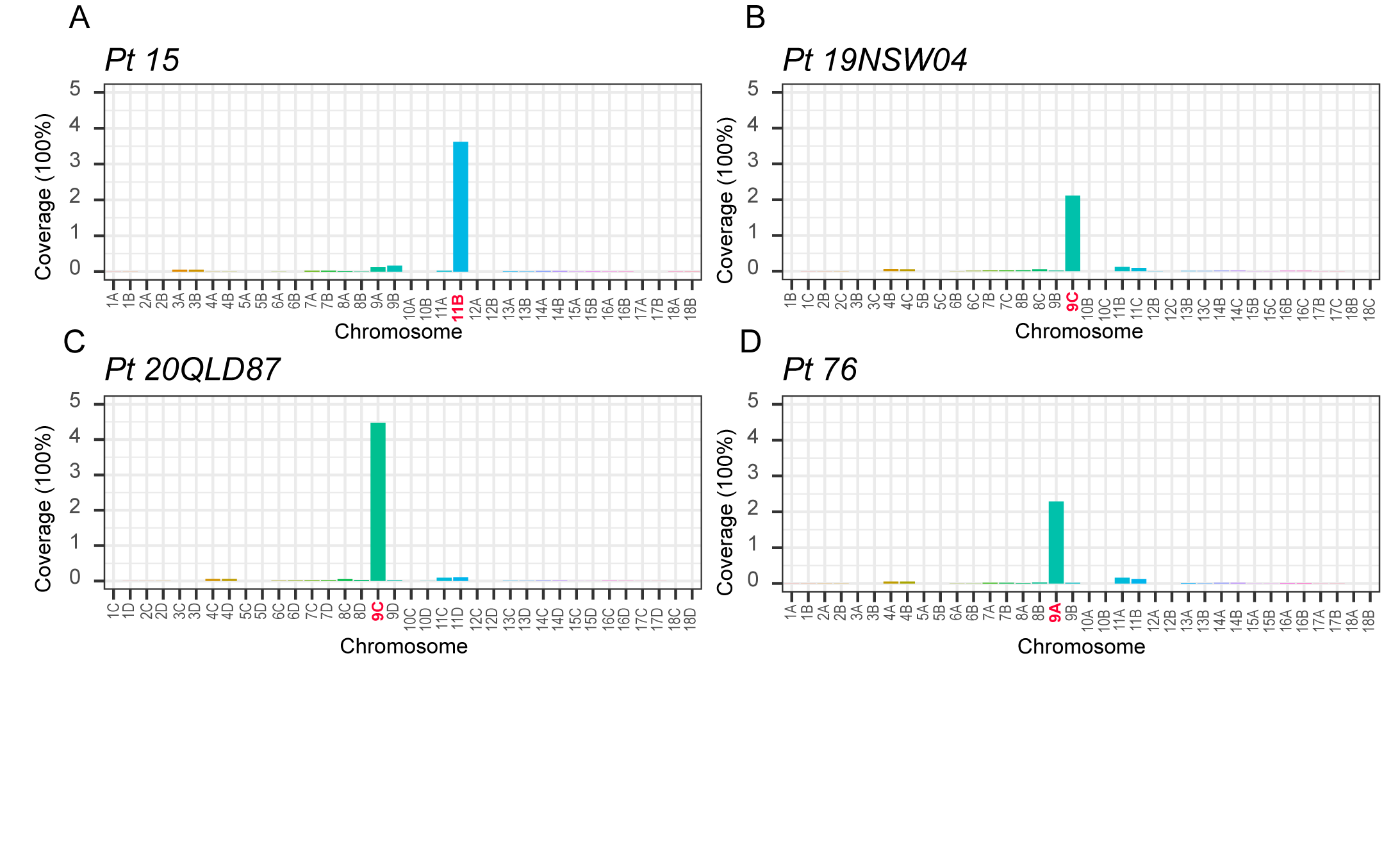

Supplement: S28 Fig — The plots show the percentage of nucleotides covered by the transposable element family Ty3_Pt_STE3.2–3 on each chromosome of four P. triticina isolates Pt 15 (A), Pt 19NSW04 (B), Pt 20QLD87 (C) and Pt 76 (D). Chromosomes carrying STE3.2–3 are highlighted in red. (TIF) [file pgen.1011207.s030.tif]

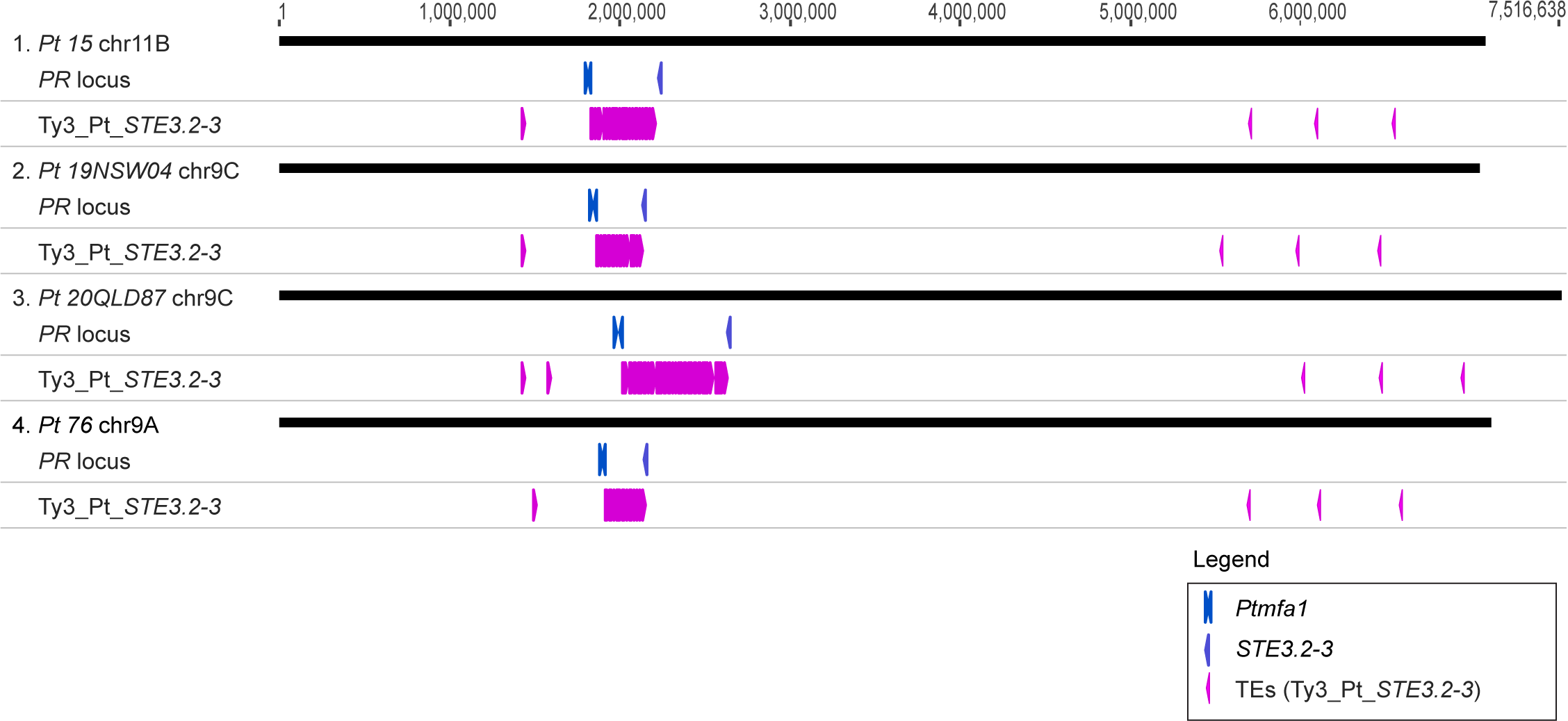

Supplement: S29 Fig — (TIF) [file pgen.1011207.s031.tif]

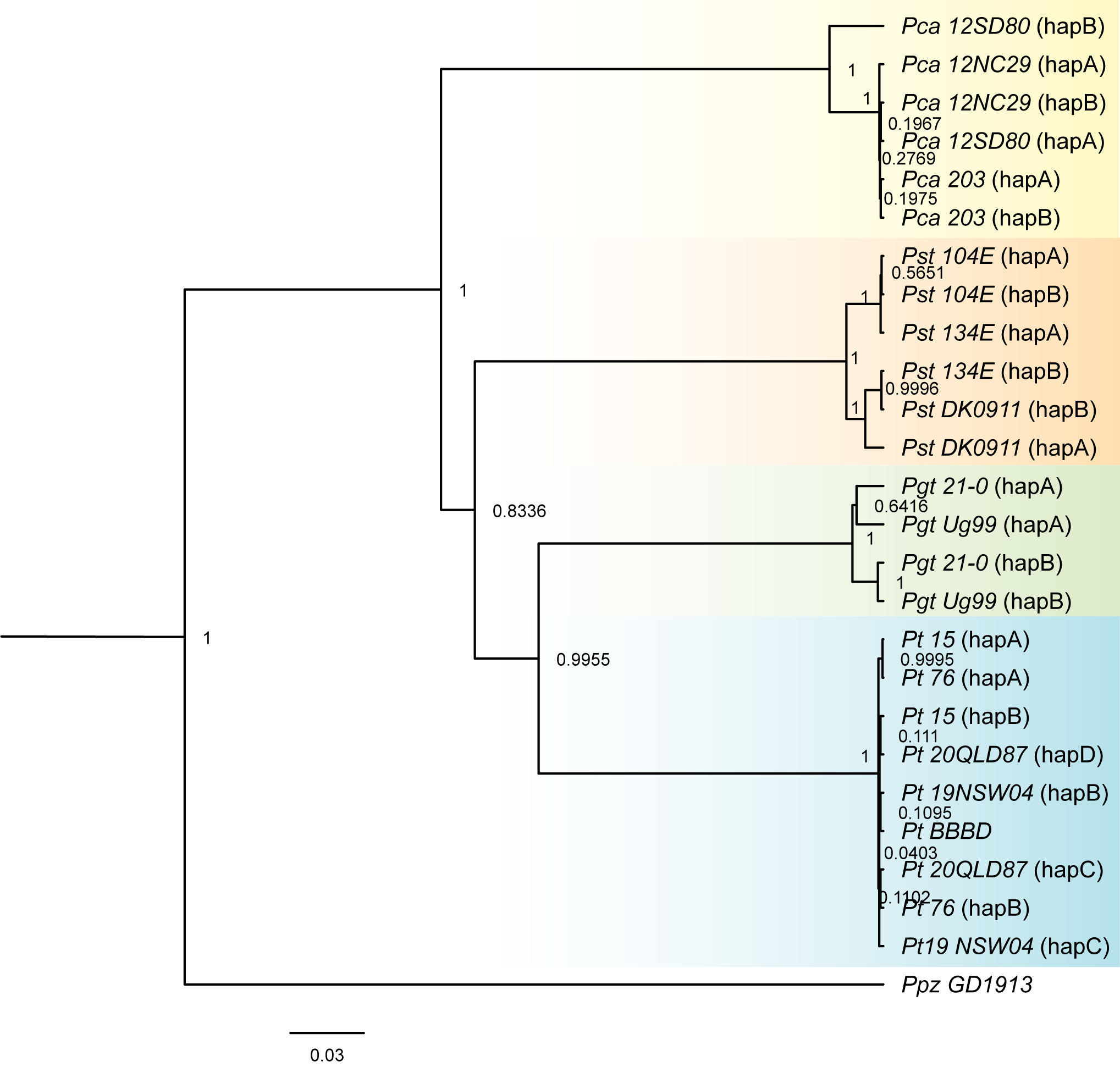

Supplement: S30 Fig — Bayesian rooted gene tree built from STE3.2–1 coding-based sequence alignment from four cereal rust fungi: P. coronata f. sp. avenae (Pca), P. graminis f. sp. tritici (Pgt), P. triticina (Pt) and P. striiformis f. sp. tritici (Pst) and P. polysora f. sp. zeae (Ppz) GD1913 was included as outgroup. Trees are based on a TN93+I model of molecular evolution. Each node is labelled with its values of posterior probability (PP). PP values above 0.95 are considered to have strong evidence for monophyly of a clade and PP values of identical alleles are not displayed. The scale bar represents the number of nucleotide substitutions per site. Alleles of the same species are colored with identical background: Pca (yellow), Pgt (green), Pt (blue), Pst (orange). (TIF) [file pgen.1011207.s032.tif]

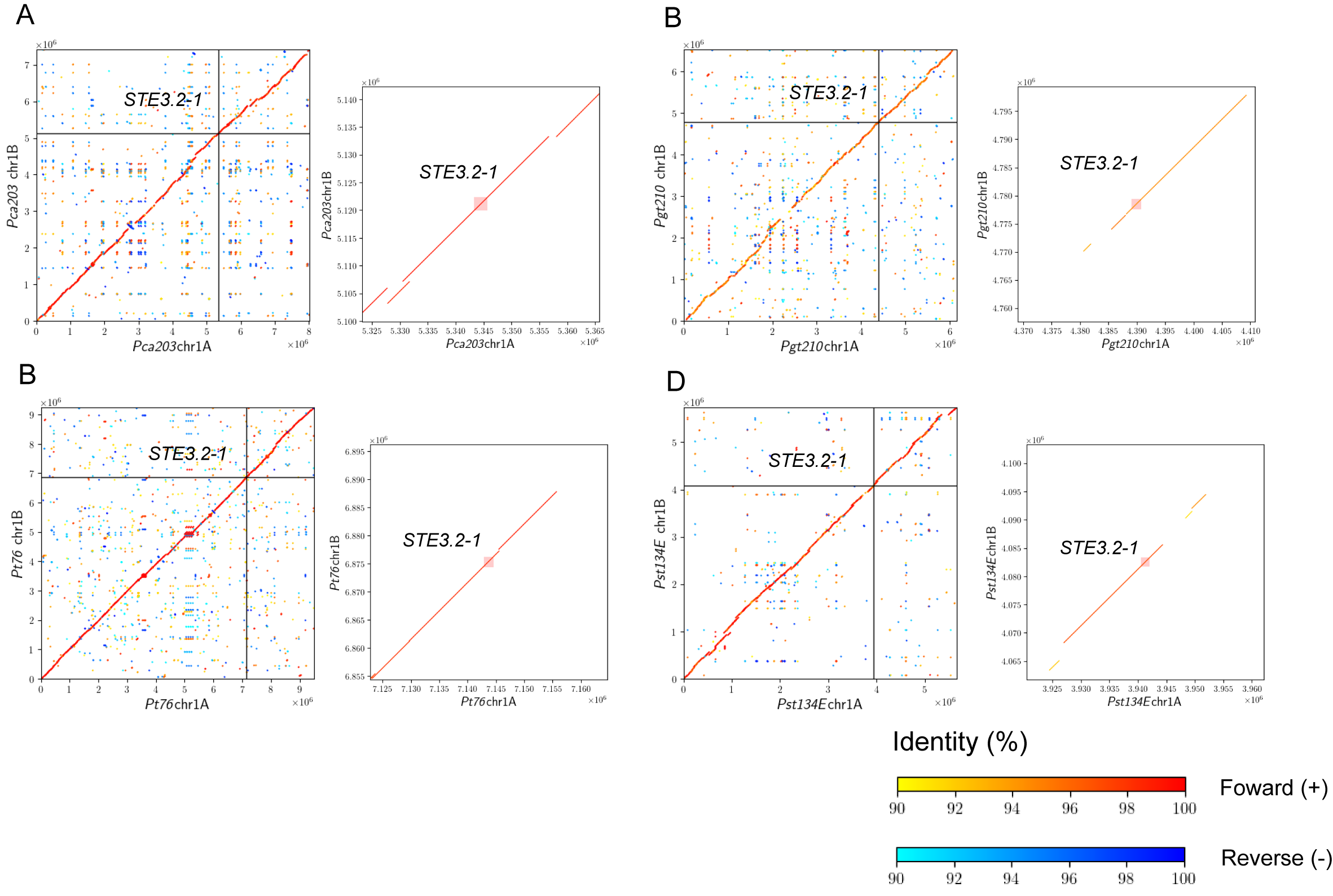

Supplement: S31 Fig — The figure shows dot plots of whole chromosome alignments between the two STE3.2–1 gene containing chromosomes from dikaryotic genome assemblies. Each panel consists of dot plots of the whole chromosome and subset dot plots zooming into the STE3.2–1 proximal region. The position of STE3.2–1 gene is labelled, and line colors show the nucleotide percentage identity and nucleotide orientation as indicated in the figure legend. Subfigures A to D show P. coronata f. sp. avenae (“Pca 203”), P. graminis f. sp. tritici (“Pgt 21–0”), P. triticina (“Pt 76”) and P. striiformis f. sp. tritici (“Pst 134E”), respectively. (TIF) [file pgen.1011207.s033.tif]

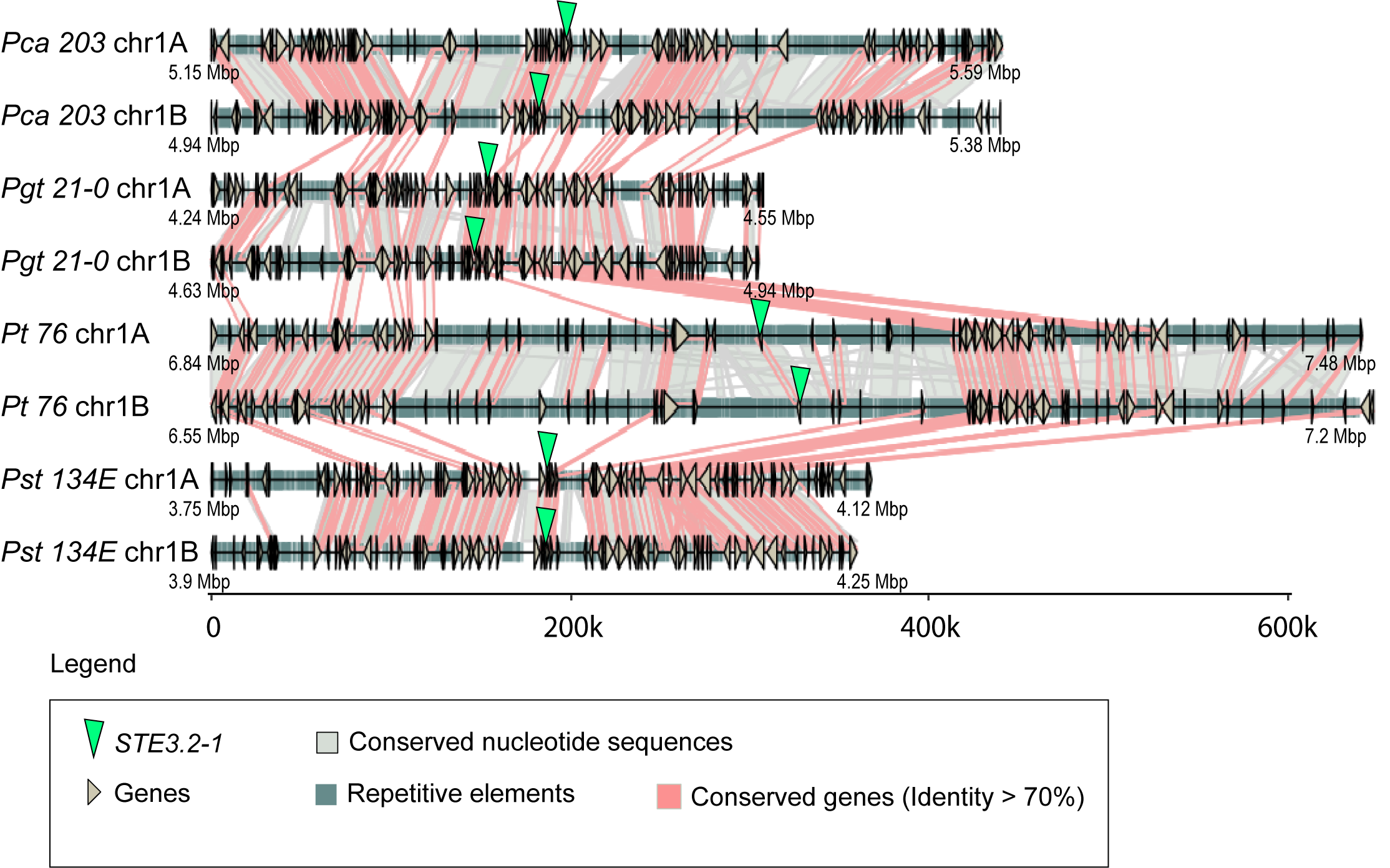

Supplement: S32 Fig — Synteny graphs of the STE3.2–1 locus including proximal regions in P. coronara f. sp. avenae (“Pca 203”), P. gramminis f. sp. tritici (“Pgt 21–0”), P. triticina (“Pt 76”), and P. striiformis f. sp. tritici (“Pst 134E”). Proximal regions are defined as 40 genes downstream and upstream of the STE3.2–1 alleles, respectively. STE3.2–1 proximal regions are highly syntenic within each dikaryotic genome and conserved between species. Red lines between chromosome sections represent gene pairs with sequence identity higher than 70% and grey shades represent conserved nucleotide sequences (> = 1000 bp and identity > = 90%). For additional annotations please refer to the provided legend (“Legend”). (TIF) [file pgen.1011207.s034.tif]

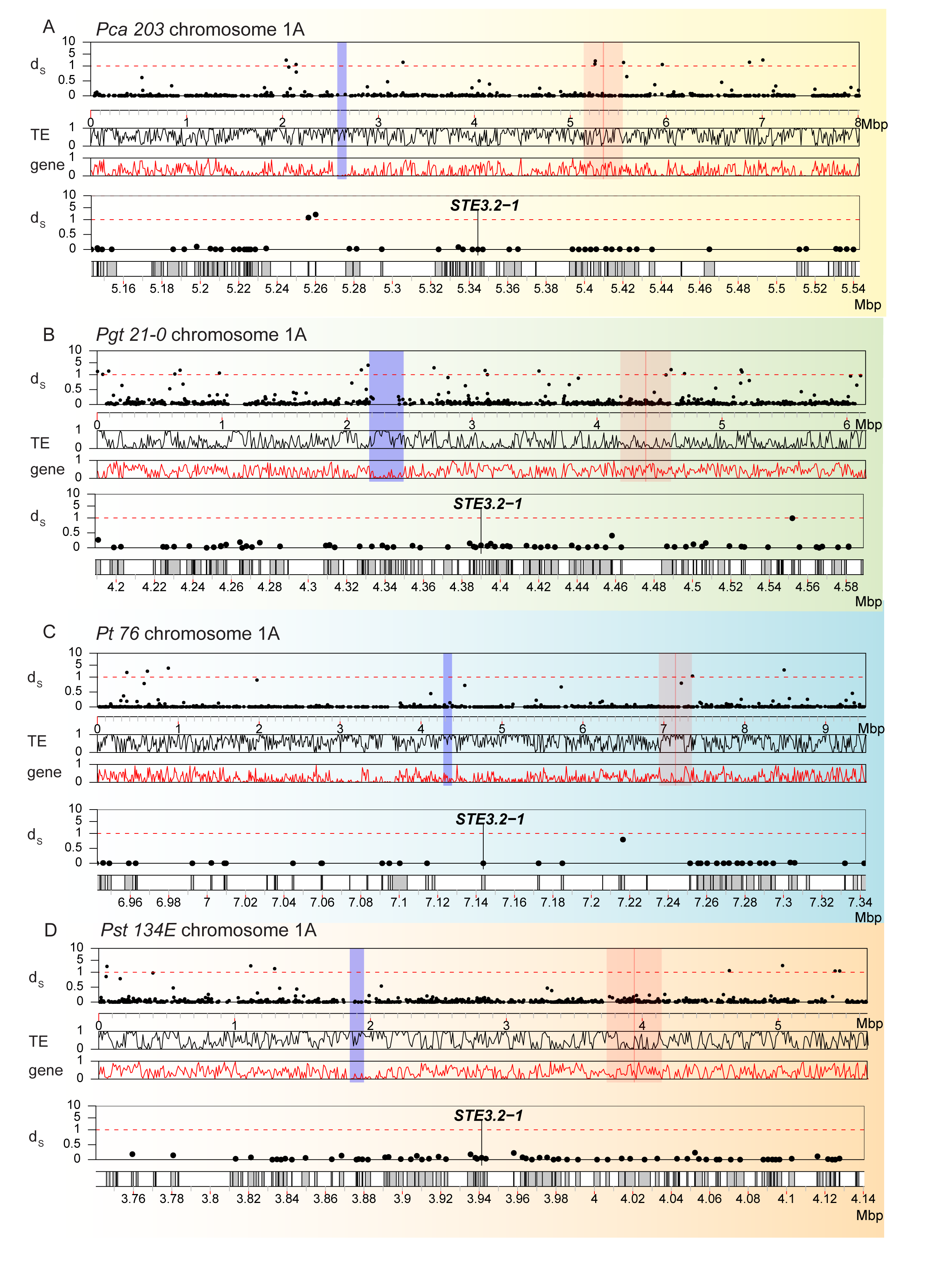

Supplement: S33 Fig — Synonymous divergence values (dS) for all allele pairs are plotted along chromosome 1A for (A) P. coronara f. sp. avenae (“Pca 203”), (B) P. gramminis f. sp. tritici (“Pgt 21–0”), (C) P. triticina (“Pt 76”), and (D) P. striiformis f. sp. tritici (“Pst 134E”). In each panel, the top track shows the dS values (“dS”) of allele pairs along chromosome 1. Each dot corresponds to the dS value of a single allele pair. The second and third track show the averaged TE (“TE”) and gene (“gene”) density along chromosome 1 in 10 kbp-sized windows, respectively. The STE3.2–1 alleles are highlighted with a red line and red shading indicates a 0.4 mbp-sized window around the STE3.2–1 genes. Predicted centromeric regions are marked with blue shading. The two lower tracks (ds values and gene locations) provide a detailed zoomed in view of red shaded area around the STE3.2–1 alleles. Species-specific background coloring is the same as for Fig 1. (TIF) [file pgen.1011207.s035.tif]

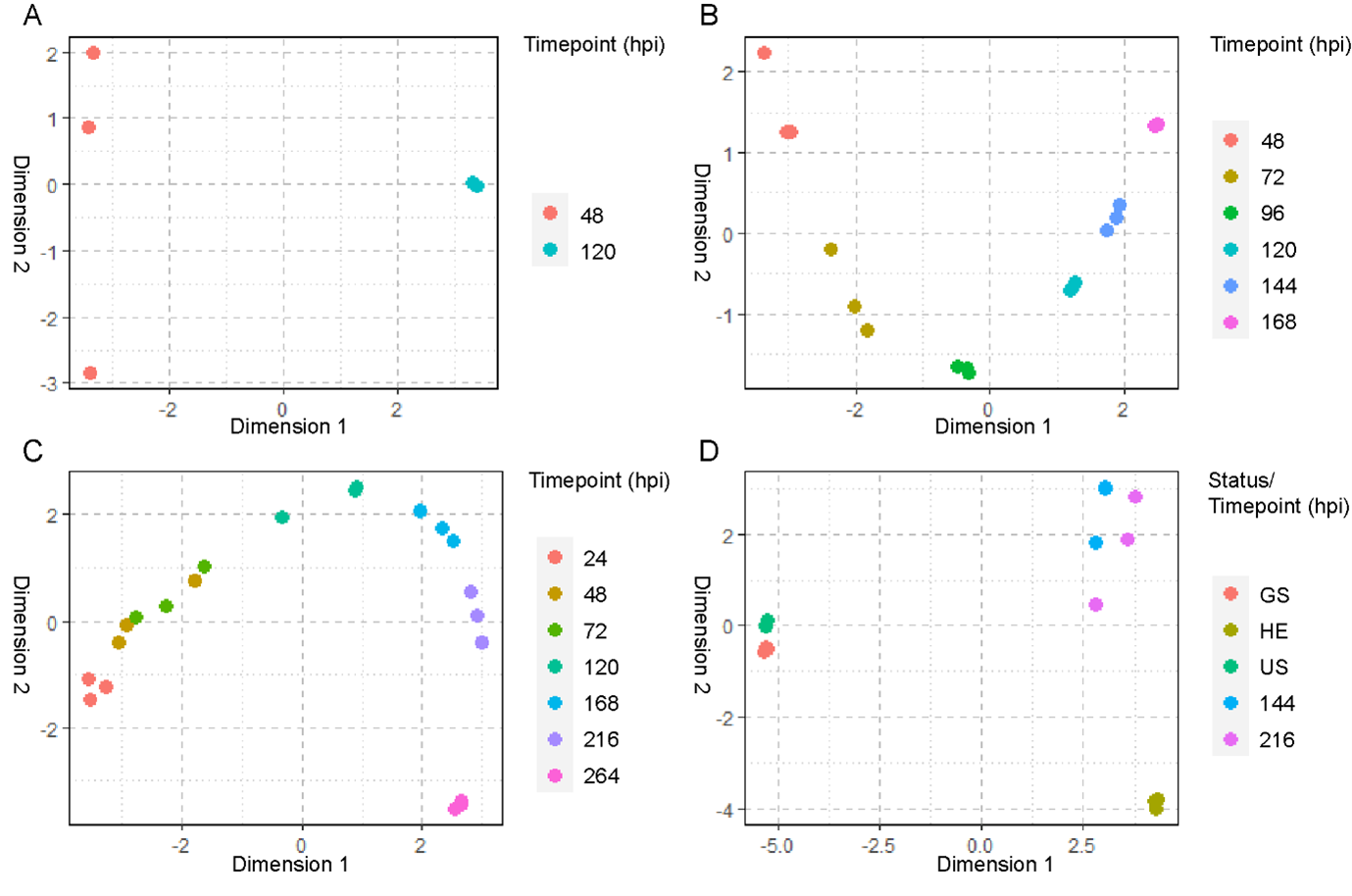

Supplement: S34 Fig — MDS plots were made with TMM normalized counts for quality control, each dot represents a single sample, and replicates were color coded as indicated in each subfigure legend. (A) MDS plot of TMM-normalized value of Pca at 48 hour post infection (hpi) and 120 hpi. (B) MDS plot of TMM-normalized value of Pgt at 48, 72, 96, 120, 148 and 168 hpi. (C) MDS plot of TMM-normalized value of Pst at 24, 48, 72, 120, 168, 216 and 264 hpi. (D) MDS plot of TMM-normalized value of ungerminated spore (US), germinated spores (GS) stages, 144 hpi, 216 hpi and haustoria enriched samples (HE) of Pst. (TIF) [file pgen.1011207.s036.tif]

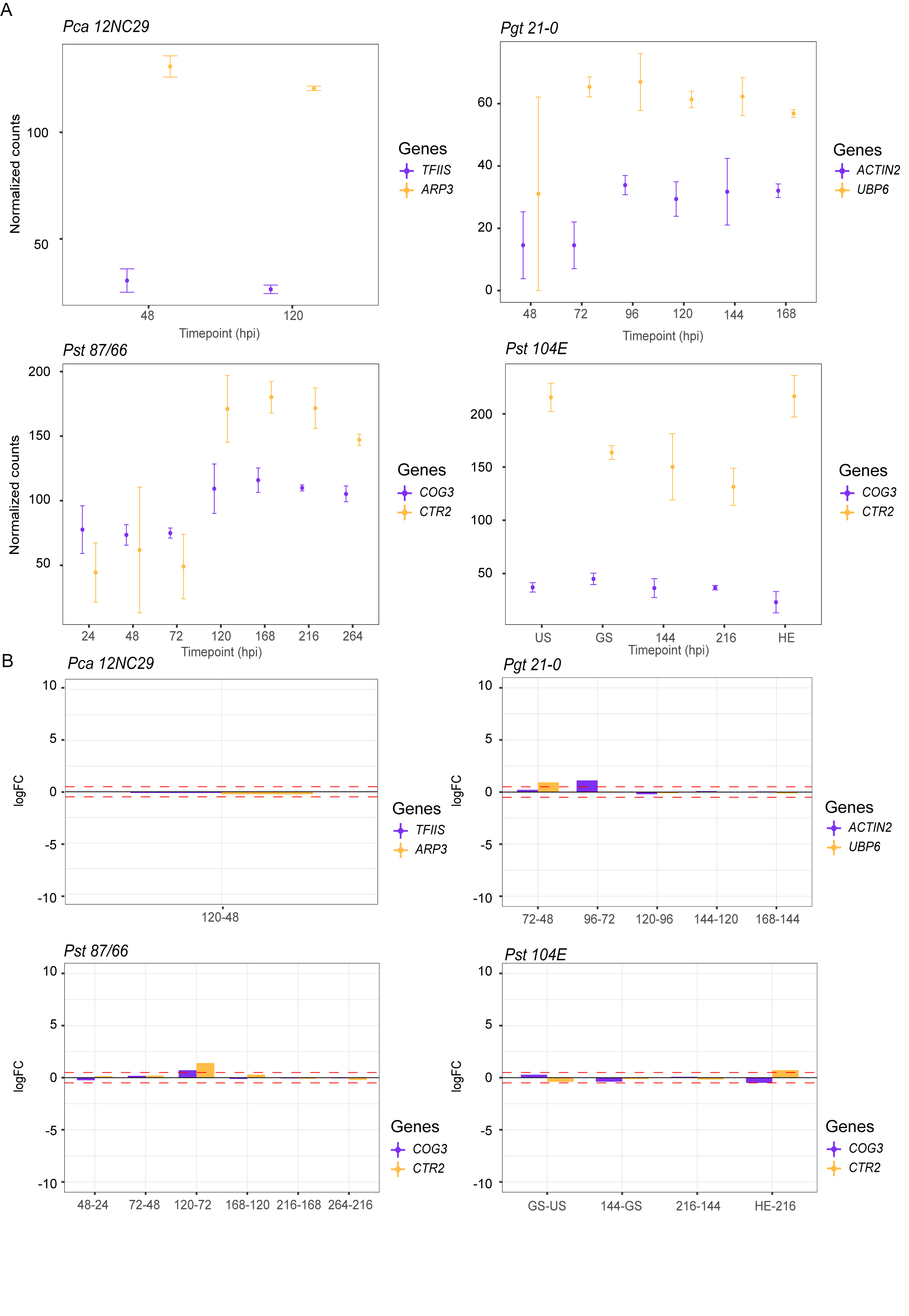

Supplement: S35 Fig — (A) TMM-normalized value of housekeeping genes in P. coronata f. sp. avenae (“Pca 12NC29”), P. graminis f. sp. tritici (“Pgt 21–0”), and P. striiformis f. sp. tritici (“Pst 87/66” and “Pst 104E”). (B) Likelihood ratio test (LRT) method was applied to test significant upregulation of housekeeping genes between timepoints, none of the housekeeping genes show significant upregulation between timepoints. red dashed lines indicate logFC = 0.5 and logFC = -0.5 respectively, stars above or below bars indicate statistically significant differences between the two adjacent time points: *p<0.05, **p<0.01, ***p<0.001. Genes are labelled with different colors. Transcription elongation factor TFIIS (TFIIS), Actin-related protein 3 (ARP3), Actin/actin-like protein 2 (ACTIN2), Ubiquitin carboxyl-terminal hydrolase 6 (UBP6), Conserved oligomeric Golgi complex subunit 3 (COG3), Ctr copper transporter 2 (CTR2). (TIF) [file pgen.1011207.s037.tif]

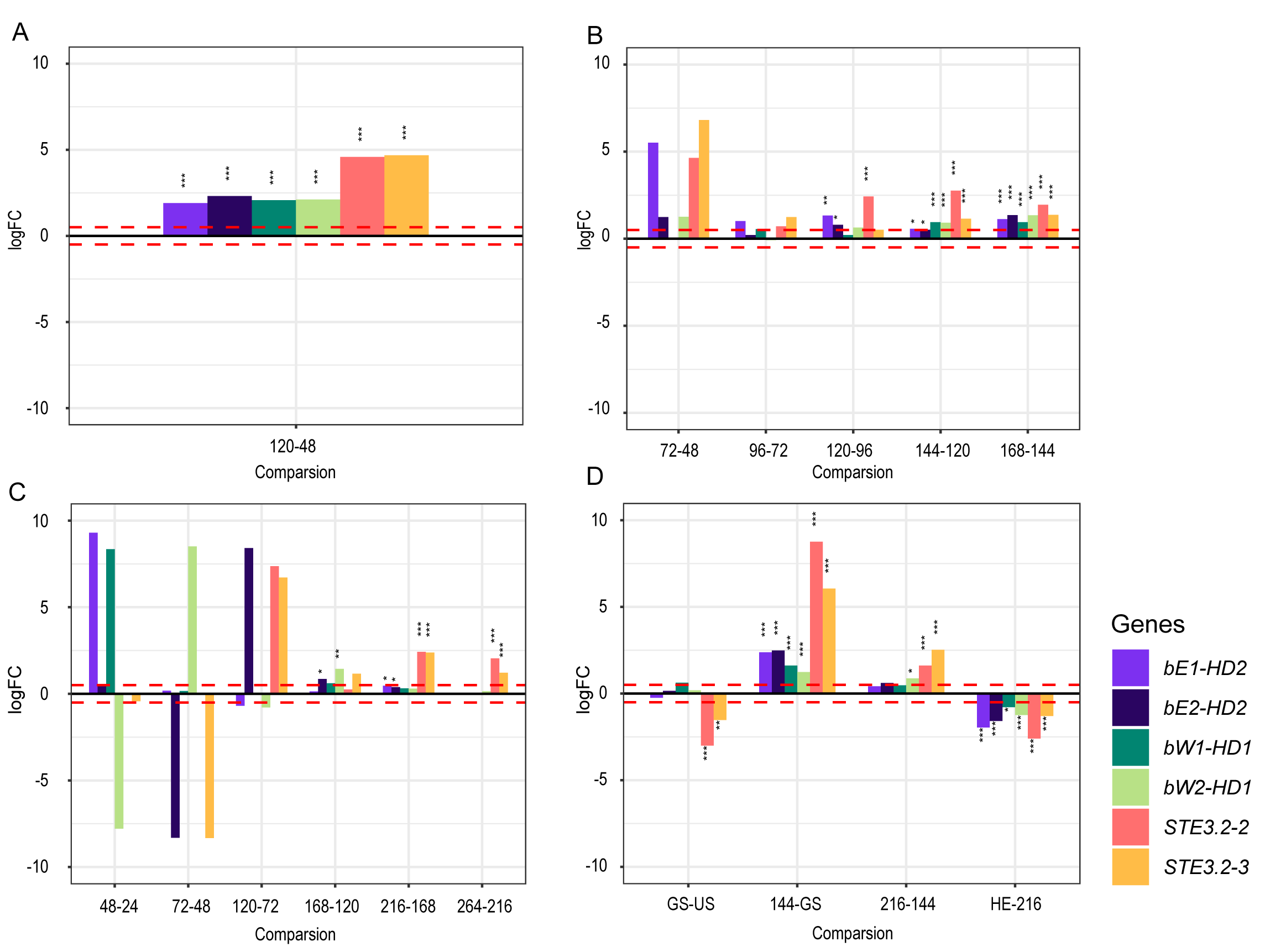

Supplement: S36 Fig — Likelihood ratio test (LRT) method was applied to test significant upregulation of MAT genes between timepoints, red dashed lines indicate logFC = 0.5 and logFC = -0.5 respectively, stars above or below bars indicate statistically significant differences between the two adjacent time points: *p<0.05, **p<0.01, ***p<0.001. MAT genes were labelled with different colors. (A) The expression levels of MAT genes in P. coronata f. sp. avenae (“Pca 12NC29”) were compared between 120 hours post infection (hpi) and 48 hpi. (B) The expression levels of MAT genes in P. graminis f. sp. tritici (“Pgt 21–0”) were compared between 72 hpi and 48 hpi, 96 hpi and 72 hpi, 120 hpi and 96 hpi, 144 hpi and 120 hpi, 168 hpi and 144 hpi. (C) The expression levels of MAT genes in P. striiformis f. sp. tritici (“Pst 87/66”) were compared between 48 hpi and 24 hpi, 72 hpi and 48 hpi, 120 hpi and 72 hpi, 168 hpi and 120 hpi, 216 hpi and 168 hpi, 264 hpi and 216 hpi. (D) The expression levels of MAT genes in P. striiformis f. sp. tritici (“Pst 104E”) were compared between germinated spores (GS) and ungerminated spores (US), 144 hpi and GS, 216 hpi and 144 hpi, haustoria enriched samples (HE) and 216 hpi. (TIF) [file pgen.1011207.s038.tif]

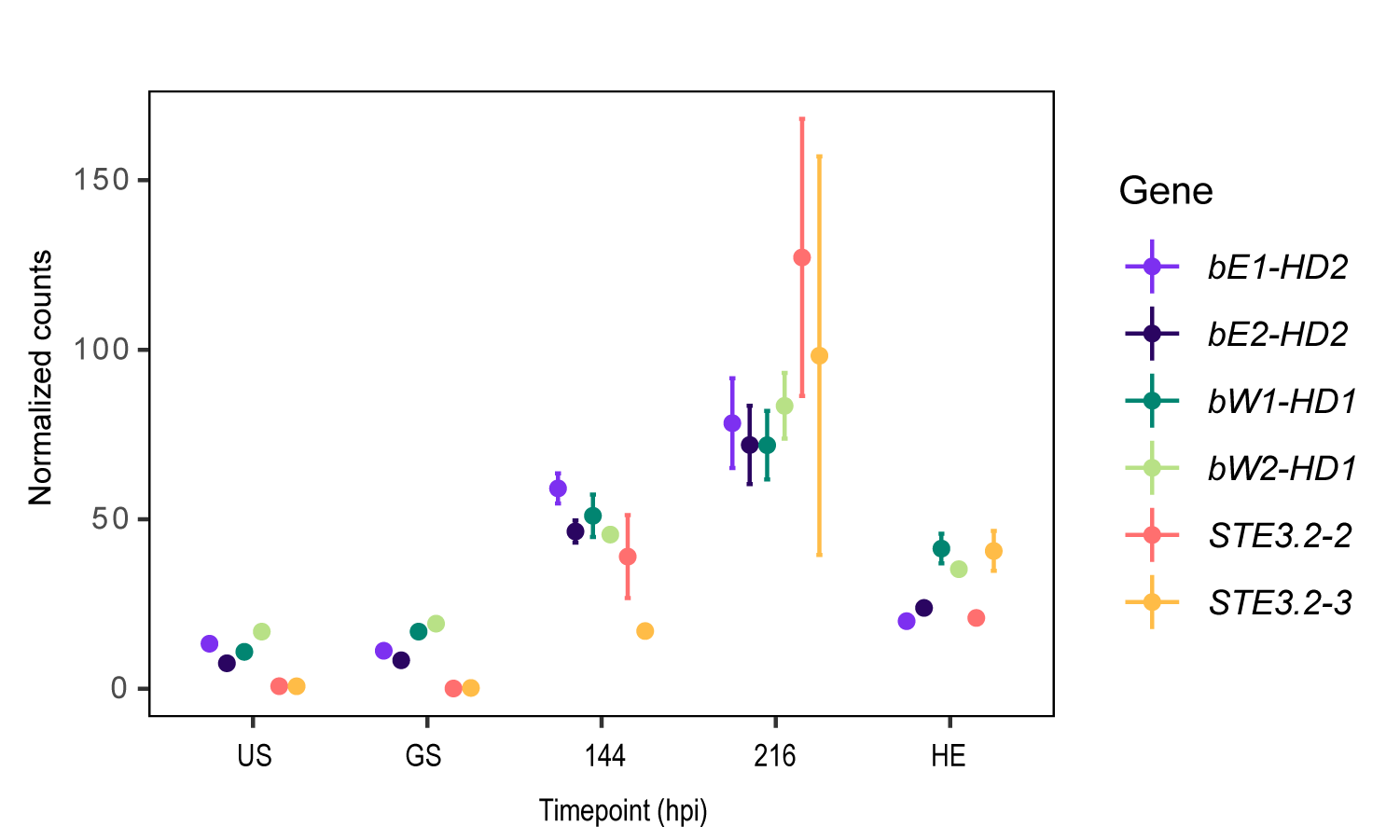

Supplement: S37 Fig — TMM-normalized value of MAT genes in ungerminated spores (US), germinated spores (GS) stages, 144 hpi, 216 hpi and in haustoria enriched samples (HE) of Pst. Genes are labelled with different colours. (TIF) [file pgen.1011207.s039.tif]
