# Supplementary material for: Genome biology and evolution of mating-type loci in four cereal rust fungi
Source: PLoS Genet. 2024 Mar 18;20(3):e1011207. doi: 10.1371/journal.pgen.1011207 (PMC10977897; doi:10.1371/journal.pgen.1011207)
Supplement: Attachment — Submitted filename: 20240223_RebuttalLetter.pdf [file pgen.1011207.s041.pdf]

Response to editors and reviewers comments for "*Genome biology and evolution of mating type loci in four cereal rust fungi*" (PGENETICS-D-23-00971R1)

The Author's response is provided in blue and bold.

Response to editor's comments:

The revised manuscript has been evaluated by the same three referees, who agree that this manuscript on the mating-type loci of rust fungi has been improved following their suggestions. The referees nevertheless still had extensive concerns, in particular about the surprising similarity between PR alleles, the delimitation of the mating-type loci, the clustering of transposable elements as well as the lack of clarity and length of the text. I have to agree with these concerns.

Author's Response: We thank the editor and the reviewers for all the in-depth expert suggestions. It really improved the manuscript significantly. We now have addressed all outstanding concerns and are looking forward to the publication in PLoSGenetics.

About the clustering of Ty elements, note that a previous study found such enrichment in non-recombining regions of mating-type chromosomes (https://doi.org/10.1038/s41467-023-41413-4).

Author's Response: Thank you very much for the positive feedback. The indicated manuscript was discussed and cited in the previous version already.

In addition, the manuscript is still not very well written. It should really be corrected by a native English specialist in the field. Some issues are highlighted below, but there are many others all along the manuscript.

Author's Response: The manuscript has new proofread by two native English speakers who are experts in the field. I hope you appreciate the improvements as much as we do.

I would encourage resubmission only if you are able to revise the manuscript along these lines. The referees also provide a list of excellent additional suggestions and questions, which should also be addressed.

Author's Response: We thank the editor and the reviewer for all the in-depth excellent suggestions. It really improved the manuscript significantly. We now have addressed all outstanding concerns and are looking forward to publication in PLoSGenetics.

E.1. -L66: it's not alleviated but increased!
Author's Response: We have corrected alleviated to increase on L64 of page 3.

-L34, L564: it's not the mating types that are tetrapolar but the species.

Author's Response: We have corrected our statement on L112 and L118 of page 5 accordingly. In general, we now refer to "mating type behavior" term extensively used in a excellent recent review Coelho et al. 2017, Fungal Sex: The Basidiomycota.

-L40, L578: correlation is between two quantitative variables; what is an allele status? Allele number?

Author's Response: We have addressed this issue and rewritten this sentence on L39 of page 2.

-L42: what does evolutionary conservation mean here?

Author's Response: We have addressed this issue and rewritten this sentence on L41 of page 2.

-L71-76: awkward and not really exact. A correct formulation would be for example: "… suggest that non-recombining fragments could fix more easily when linked to biallelic permanently heterozygous loci such as mating-type loci due to the sheltering of deleterious mutations. Indeed, non-recombining fragments with fewer deleterious mutations than average are beneficial and can rise in frequency, but they will suffer from exposing their (few) recessive deleterious mutations at a homozygous stage when becoming frequent, except if they are linked to a permanently heterozygous locus that will shelter them."

Author's Response: We have addressed this issue and rewritten these sentences from L71 to L76 on page 4.

-L77: predicts that

Author's Response: We have corrected 'predict that' to 'predicts that' on L81 of page 4.

-L85-95: keep the general ideas first (i.e. from L91) and the specific statements later (i.e. from L85).

Author's Response: Thank you so much for the suggestion, we have rewritten this paragraph based on the suggestion and would like to keep the remainder as in our publication. Please see page 4 to page 6 for details.

-L107, L118: « heterothallic » is not a mating system (heterothallic fungi can undergo both outcrossing and diploid selfing)

Author's Response: We have rewritten this sentence and removed the term 'heterothallic' on L108 and L109 of page 5.

-L211: tetrapolar is not a mating system either, it does not control selfing and outcrossing either

Author's Response: We have rewritten all sentences mentioned 'tetrapolar' as mating system throughout the manuscript, this issue was corrected on L215 of page 9. In general, we now refer to "mating type behavior" term extensively used in a excellent recent review Coelho et al. 2017, Fungal Sex: The Basidiomycota.

-L221: suggested that (« that » should not be omitted in written text)

Author's Response: We have rewritten this sentence as 'This is consistent with previous preliminary analyses for *Pgt* and *Pt* that suggested HD and PR loci are located on two distinct chromosomes' on L226 of page 10.

-L370: consistent with what ? what does « order level » mean ?

Author's Response: We refer to Wicker et al, 2007, A unified classification system for eukaryotic transposable elements TE classification system, which has attracted over 3000 citations to date.

We follow Wicker et al's classification system of Class-Order-Superfamily-Family system throughout the manuscript to be consistent with generally accepted conventions in the scientific community. We have added the citation of this paper more prominently and added further clarifications throughout the text.

-L374, L404, 462: what is conserved ? gene order ? sequenced identiry ? « conserved » makes no sense « within genomes », conserved applies to long evolutionary times, do you mean are similar ?

Author's Response: We have now addressed this issue by specifying what is conserved in all cases e.g. sequence identity.

-L382, 383 and elsewhere : why loci plural ? There is a single locus, even across species, these are not different loci

Author's Response: Thank you so much for pointing out this issue, we have fixed this throughout the manuscript.

-L388: why mbps plural ?

Author's Response: Thank you for pointing out our mistake, we have corrected this issue on L406 of page 17.

-l389: well, it's not concomittant, it's causal : it's because TE have accumulated that the regions are gene poor

Author's Response: We have removed 'concomitant' and rewrote this sentence from L405 to L407.

-L422: awkward : the polymorphism exist regardless of whether you map (so not only « when mapping «)

Author's Response: We have addressed this issue and rewritten this sentences from L434 on page 18.

-L426: isolates are not populations

Author's Response: We have addressed this issue and rewritten this sentence L440 on page 19.

-L430: again, the locus is the same across species so the formulation is incorrect, the locus is not specific to Pca, the sequence is

Author's Response: We have corrected this issue throughout the manuscript.

-L449: gave rise is not optimal formulation

Author's Response: We have addressed this issue and rewritten this sentence on page 19.

-All along the ms : check that you add a hyphen between two words that qualifies a third one, e.g. L477 protein-coding gene, or L455 chromosome-scale, otherwise your long sentences are hard to read

Author's Response: Thank you for pointing out this issue, we have added hyphen for protein-coding gene, short-reads and long-reads.

P21-22: this is hard to read, much too detailed

Author's Response: We have addressed this issue and rewritten these sentences on page 20 to 22.

-L519: MAT loci are not involved in sexual reproduction per se but in mate compatibility, this is very different. The HD locus is also involed in dikaryotic growth, why shouldn't this be also the case in asexual reproduction ?

Author's Response: We addressed the issue of "mate compatibility" L575 on page 24. We do not appreciate that the manuscript needs any other changes in this regard.

-L541: any expression

Author's Response: We have added 'any' for this sentence on page 24.

-L565: as already said in a previous version, it's not that multiallelism is beneficial under outcrossing (this is wrong group selection reasonning, evolution does not act for the good of populatio,), but that rare alleles are selection for under outcrossing and leads to multi-allelism, this is very different.

Author's Response: We have addressed this issue and rewritten these sentences L594 and 595 on page 25.

-L624: they actually follow the same trend
Author's Response: We have rewritten this sentence to make it more clear on page 27.

Response to reviewer 1's comments:

Reviewer #1: Comments on rebuttal (Jan 2024)
I very much appreciate that the authors took most of the comments and issues from the 4 (!) reviewers to heart and seriously revamped the study. Hence, in my opinion, the manuscript has been substantially improved. All of the issues we raised have been addressed, which made the supplementary data set quite large: but the many added mapping data, alignments and redone (molecular) phylogenies made the data much more supportive of the conclusions. Also, generating the many HD alleles de novo for proper comparisons and subsequent conclusions added much clarity to the manuscript.

The intro is now much more focused on the evolution of the mating-type region and known literature; many concepts are now introduced.

Author's Response: We greatly appreciate the expert in-depth feedback of the reviewer. It has really improved our manuscript.

Some minor suggestions:
-L25, right at the start of the abstract: I don't quite get the term "Obligate heterozygous loci such as". I would delete this whole first sentence since you come back later to it.

Author's Response: We have rewritten the first sentence of the abstract and removed 'Obligate' on L25 of page 2.

-L27, delete "for"

Author's Response: Thank you for pointing out this mistake, we have deleted 'for' on L27 of page 2.

Line 28: suggestion: "To date, an analysis of genome-level mating-type (MAT) loci is lacking in the obligate biotrophic basidiomycetes in…."

Author's Response: Thank you for the suggestion, but we decided to keep using 'lacking for' in this sentence to avoid using 'in for' twice.

-L32: "P. graminis…."

Author's Response: Thank you for pointing out this mistake, we fixed this issue on L32 of page 2.

-L34: "tetrapolar mating systems in the Pucciniales. The HD locus is found to be multiallelic…."

Author's Response: We have fixed this issue by rewriting this sentence on L34 of page 2.

-L36: "were.."; switching to past tense compared to he sentence above. Be consistent!

Author's Response: Thank you for pointing out this mistake, we fixed this issue on L35 of page 2 and checked carefully throughout the text.

-L39: "…with a clear biallelic PR locus"; you already said this a few sentences before….

Author's Response: Thank you for pointing out this issue, we have rewritten this sentence to make it tidy.

-L43: "…is related to correct nuclear pairing during spore formation."; is unclear. IS meant: "..may play a role in maintenance of a proper dinuclear state.", or something?

Author's Response: We have rewritten this sentence on L42 and L43 of page 2. Yes the meaning here is 'may function in maintenance of proper pairing'.

-L85: delete "behind"

Author's Response: We have fixed this issue on L130 of page 6.

-L143: "The role of MAT genes during the life cycle of rust fungi is rudimentary. Though it is hypothesized that MAT genes regulate appropriate nuclear paring during dikaryotic spore production and mediate the compatibility of haploid cell fusions on the "alternate" host"; I do not understand the first sentence, what is meant by "rudimentary", the knowledge of their role(s)? Also, the hypothesis brought forward is heavily based on molecular work in the smuts; maybe this should be mentioned (and a ref).

Author's Response: Thanks for pointing out this issue, we have reworked the first sentence on L150 and added references from L151 to L152 of page 7.

-L202: "unequivocal"; in the abstract the term "appears to be biallelic" is used, which is more carefully phrased. You show the presence of two alleles here in the few surveyed isolates; it's nuanced in the Discussion, with Pgt potentially have more alleles. This may may be confirmed when more isolates genomes are generated worldwide in the future. Even for the other three investigates species; can this be excluded?

Author's Response: We remove the term "unequivocal" and phrased these hypotheses more carefully throughout the text.

-L292: I am not sure that the two genes bE-HD2 and bW-HD1 actually share the same promotor; yes, they are tightly linked, physically close together and outwardly expressed and may depend on similar/the same transcription induction elements, but I don't think the can be defined as the same promoter. Experiments will need to be done to ascertain this.

Author's Response: We have rewritten this sentence to remove the statement which mentioned 'share the same promotor' from L96 to L102 of page 5.

-L296: "…. , which might be caused by recombination within the HD locus." Could different rates of genetic drift (accumulation of nucleotide mutations) followed by different selection pressures be the cause?

Author's Response: We performed additional analysis for detecting recombination events based on Reviewer 2's suggestion. These results further support our initial hypotheses that our observations are more likely to be caused by recombination. We also added further citations to this section.

-L662. Wrt MAT genes expression during spore stages, Zhan e al., 2023: https://doi.org/10.1007/s44154-023-00107-z, can be cited here again (you already mentioned this ref earlier). Ironically, their data set, though publicly available, cannot easily be checked for these MAT genes!

Author's Response: Thank you for the suggestion, we have added the citation of *P. striiformis* f. sp. *tritici* in this sentence. We also requested their raw RNA-seq datasets for re-analysis, however without success. Yet we will expend on analysing the complete sexual cycle of *Pst* more closely with our collaborators.

-Data availability: the link to the repository Dryad (https://doi.org/10.5061/dryad.w0vt4b8zm) does not seem to work.

Author's Response: We apologized for this mistake, the uploaded data got approved recently, this link is available now.

Response to reviewer 2's comments:

Reviewer #2: Luo and colleagues have commendably addressed the concerns and suggestions from my initial review, significantly enhancing the quality and clarity of their manuscript. Notably, improvements in terminology, formatting, figures, and content have been made. The extension of the methods section, coupled with the provision of raw data on Dryad and analytic code on GitHub, is particularly appreciated for its contribution to transparency and reproducibility.

In response to specific points raised in my initial review, the authors have opted to maintain the nomenclature of HD1 (as bWest) and HD2 (as bEast) genes, but with added clarifications provided in the text. Additionally, they provide a more comprehensive approach to explore HD allele diversity and of variability in the N-terminal regions of HD. The authors also acknowledge the limits of their dataset regarding STE3.2-1 expression and added relevant references to similar findings in other fungi.

In this revised evaluation, I have focused on assessing how these and other changes have impacted the overall narrative and quality of the manuscript. Below, I provide additional comments and suggestions on specific points. Overall, the manuscript has undergone significant improvements and is moving closer to aligning with the high standards of PLOS Genetics.

Author's Response: Once again, some excellent points raised by the reviewer for which we are very grateful.

Major points

1) PR and HD locus regions: The manuscript would benefit greatly from a clearer delineation of what the authors consider to be the PR locus region in each of the haplotypes for the species studied. While the dot plot analysis provides valuable information, an overarching view of the synteny for the PR-containing chromosomes in each species would significantly enhance the clarity and comprehensiveness of the findings. The HD locus region (only HD1-HD2?) should also be highlighted.

Author's Response: Thank you so much for the suggestion, we defined both loci more explicitly in accordance with accepted standards in the community e.g. Maia et al 2015.

For the HD locus see L342 and 343 on page 15: "We defined the HD locus as the DNA fragment which includes bW-HD1, bE-HD2, and the DNA sequence in between."

For PR locus see L395 and 396 on page 17: "We investigated organization and synteny of the PR locus which we defined as the DNA fragments including Pra, mfa genes, and DNA sequences in between."

2) Mature Pheromone Peptides Predictions: In the section concerning the predicted Mfa1 and Mfa3 proteins in Pgt (Lines 266-268 and Fig S3), the authors mention the possibility of distinct mature pheromone peptides being produced by these two genes, each potentially having different receptor specificities. However, the authors don't provide the predicted sequences for these mature peptides. Clarifying these sequences is important for assessing their specificity and understanding the functional implications of these differences. Upon closer examination, it appears that the Mfa1 precursor may encode multiple copies of the putative peptide moiety. For instance, the Ptmfa1/3 precursor contains two copies of the sequence "QWGNGSHIC" with CAAX motifs separated by a spacer sequence. This contrasts with the Mfa2 precursors, which seem to encode only one mature peptide. This pattern of multiple peptide encoding is also observed in other Pucciniomycotina species, such as Rhodotorula spp. and Microbotryum spp. and also noted in other Pucciniales.

Author's Response: Thank you so much for this valuable comment, we now added predicted mature peptides in S3 and S23 Figs, and extended the relevant section from L268 to L282. The specific analysis revealed issues with previous mfa gene predictions in in Cuomo et al. (2017) which we overlooked initially. Our re-analysis revealed that we previously missed the first repeat peptide in *Pgt*, *Pst* and *Pca*. Indeed, it seems that *mfa1* precursor may encode 1-3 distinct mature peptide in these species, whereas *mfa2* and *Pgt-mfa3* might only encode one mature peptide. We added appropriate citations of Microbotryum spp and Ascomycetes.

3) Hi-C data and PR/HD locus linkage to centromeres: The conclusion that HD loci are likely not directly linked to centromeres, derived from Hi-C data, warrants further clarification. How does this method definitively determine the absence of direct (genetic?) linkage, and were these assessments also made for the PR locus? The potential limitations in resolution or mapping in complex genomic regions should also be addressed.

Author's Response: We state in the text "physical" linkage as we are not able to address "genetic" linkage in the current study. We do not think that further additional analysis is required for this point as it is no the focus of our current manuscript.

4) Evolutionary history of STE3.2-2 and STE3.2-3 alleles: An intriguing aspect noted is the considerable identity shared between the STE3.2-2 and STE3.2-3 alleles within the same species (~43% identity, ~56% similarity). This raises an interesting question about their evolutionary history. Do you think this level of similarity could indicate a more recent allele turnover, potentially following the loss of the STE3.1 allele variant? This seems particularly noteworthy when considering that in other Pucciniomycotina species, the STE3.1 and STE3.2 alleles are more divergent and often not alignable for dS calculations. Furthermore, I am curious about the divergence rates of non-MAT genes within the presumed PR locus region. Specifically, do these non-MAT genes exhibit increased synonymous substitution rates (dS)

compared to other genes located on the same chromosome but outside the putative PR region? This assumes, of course, that there are genes common to both haplotypes within the PR region, allowing for such a comparison. Alternatively, is the gene content within the PR region too divergent between the haplotypes to enable a meaningful comparison of substitution rates?

Author's Response: Thank you for this insightful question.

(1) We don't know enough about the evolutionary origin/history of STE3.1 to make a meaningful comparison to the functional alleles at the PR locus and we believe to truly resolve the divergence of the STE3.1, the community will need to examine this gene family in other Pucciniomycotina (Septobasidiales, Platygloeales).
(2) In our Fig 4B, we observed a syntenic gap around the *Pra* and *mfa* genes without sufficient allele pairing to perform the suggested d_S calculations of non-MAT genes.

5) Clustering of Ty3_Pt_STE3.2-3 TE family: The observation that the Ty3_Pt_STE3.2-3 TE family predominantly clusters on chromosomes containing the STE3.2-3 locus, particularly within a region bordered by the pheromone and receptor genes, is a notable finding. This specific clustering suggests restriction of this element in this specific genomic region. I'm curious about several aspects regarding this observation. Firstly, what might be the potential mechanisms that restrict the Ty3_Pt_STE3.2-3 to the PR STE3.2-3 locus? Could there be unique genomic or epigenetic features in the vicinity of the STE3.2-3 locus that might explain why this TE family does not transpose more frequently to other genomic regions? Secondly, Have similar specific TE elements been observed within the STE3.2-2-containing haplotype? Thirdly, the absence of a GAG domain in Ty3_Pt_STE3.2-3 TEs is intriguing. Given that GAG domains are typically associated with the structural proteins of TEs, could this absence affect the mobility or functionality of these TEs?

Author's Response:

(1) This is an excellent question, and we think it might be the genomic context and/or the 3D dimensional genome organization of the STE3.2-3 locus. However, we do not want to add further speculation to the main text of our current manuscript.

(2) We didn't observe any specific TE family in *STE3.2-2* containing haplotype like *STE3.2-3*, the coverage of major TE families around *STE3.2-2* are similar across the four isolates we studied.

(3) We also have similar opinions, but we failed to find relative references which can support this hypothesis so we didn't mention this in-text as it is not a focus of our current manuscript.

Minor points:

1) Lines 28-30, Abstract section: The statement, "To date, genome-level mating type (MAT) loci analysis is lacking for obligate biotrophic basidiomycetes in the order Pucciniales, which contains many economically important plant pathogens," could be refined to highlight the novel aspects of the study more explicitly, by utilizing chromosome-level and phased genome assemblies to analyze mating type (MAT) loci.

Author's Response: We adjusted the abstract to improve highlighting the novelties of our study.

2) Line 95/97: "PRA" and "MFA" nomenclature. Please double-check and confirm that the use of this nomenclature for proteins and genes is the adopted by the journal and fungal genetics.

Author's Response: We addressed this issue by using italic for genes (e. g. *HD* genes), capital non-italic for loci (e.g. PR locus) and *Pra* for the pheromone receptor genes.

3) Line 97: use of "matching allelic PR locus", perhaps would more clearly expressed as "compatible PR allele."

Author's Response: Thank you for the suggestion, we have corrected this sentence based L95 of page 4.

4) Line 100: The phrase "also known as bE-HD1 and bW-HD2" could be rephrased for clarity, and to provide a historical context, to "originally designated as bE-HD1 and bW-HD2 in Ustilago maydis" and include in addition, the original reference where this designation was first established.

Author's Response: Thank you for the suggestion, we have corrected this sentence and added relative citation on L99 of page 5.

5) Line 121: "ten to hundred known…", consider revise to "ten to hundreds of known…"

Author's Response: We have corrected this sentence based on this suggestion on L119 of page 5.

6) Lines 140-143: The discussion about cell types and ploidy changes during the life cycle is important in the context of understanding the evolution of non-recombining regions. Therefore, it might be beneficial to consider citing and contextualizing this information in light of the recent findings by Talhinhas et al. (Microbiol Spectr. 2023 11:e0153223. doi: 10.1128/spectrum.01532-23). This study reports the widespread occurrence of replicating haploid and diploid nuclei in various life cycle stages of Pucciniales species, suggesting a unique life cycle that is distinct from traditional haplontic, diplontic, or haplodiplontic cycles.

Author's Response: Thank you for this suggestion, we have cited this paper on L149 of page 7.

7) Lines 293-295: In the context of discussing tree topologies in phylogenetic analysis, using the term "congruent" instead of "concurrent" might be more accurate and descriptive. Furthermore, since the authors suggest the possibility of recombination within the HD locus, conducting a more direct test of recombination employing software like RDP5 could help substantiate this hypothesis. Although different from the hypothesis proposed here, I also recommend considering the study conducted on Ustilago maydis as a pertinent reference as it demonstrates how novel HD specificities can arise from single homologous recombination events, leading to simultaneous changes in the dimerization subdomains of the bE and bW proteins (see Kämper J, et al. 2020. New Phytol. 228:1001-1010. doi: 10.1111/nph.16755; also check Perlin MH. 2020. New Phytol. 228:799-801. doi: 10.1111/nph.16847).

Author's Response: Thank you for this suggestion, we applied RDP5 for detecting recombination events and found potential recombination events similar to Kämper et al. 2020 proposed in

***Ustilago maydis*, we have uploaded corresponding RDP5 project on Dryad as supporting information and cited Kämper's work on L315 to L318.**

8) Lines 387-391: In the section discussing gene density around PRA genes, it is mentioned that STE3.2-2/STE3.2-3 are located within or adjacent to 'gene deserts' and these regions are enriched in TEs. However, the text refers to Pgt 21-0 as an exception, but it's not entirely clear what this exception pertains to. Does the exception refer to the lack of a 'gene desert', the absence of TE enrichment, or a different pattern of gene density around the PR locus in Pgt 21-0? Clarification on this point would help in better understanding the unique characteristics of the PR locus in Pgt 21-0 compared to the other species studied.

Author's Response: Thank you so much for pointing out this issue. Indeed, we wanted to refer the exception such as the lack of extensive "gene deserts". We rewrote this sentence with more details L406 and L407 on page 17, accordingly.

9) Upon reviewing the data presented in S12 Fig related to the TE composition in Pra proximate regions, I noticed that although the proportions of different transposable elements vary, it appears that the classes of TEs present are more or less the same across the different cereal rust fungi. This observation seems somewhat contradictory to the statement in the manuscript (lines 399-402) about the varied TE composition and accumulation in these regions. If so, please clarify.

Author's Response: We classified TEs based on Wicker's classification system as highlighted above as response to the editor. S12 Fig (now S11 Fig) was generated based on the total coverage of TEs classified at the order classification level, whereas the sentence here refers to family level. We deposited a table which contains the coverage of the most abundant TE families on Dryad as supporting information. This type of analysis demonstrates distinct TEs composition at the superfamily and family classification level.

10) Line 421: S18 Fig seems to refer to Pgt not Pca.

Author's Response: Thank you so much for pointing out this mistake, we have fixed this issue L435 of page 18.

11) Line 434: Not sure that "de novo assembly" should be considered a "novel" approach.

Author's Response: We removed the 'novel' for the sentence on L448 of page 19.

12) Line 455-461: Regarding the assessment of more than two functional Pra alleles in Pgt, I am not entirely convinced by this conclusion. It seems more likely that the observed variations are variants of the same allele class, a common occurrence when comparing more divergent strains or lineages. A more cautious or nuanced discussion of this finding would be beneficial, considering the high degree of allele variation in fungal species. Additionally, the authors indicate that mfa1 and mfa3 in Pgt had several non synonymous changes and that this is consistent with significant variation observed in STE3.2-2 and

STE3.2-3. However, I think would be informative to determine if the observed variations at the mfa1 and mfa3 loci translate into differences in the mature peptides produced.

Author's Response: Thank you so much for these valuable suggestions, it is likely that mature peptide produced by *Pgt-mfa3* ended with 'SHYC' which only has single amino acid replacement when compared to the predicted mature peptide produced by *Pgt-mfa1*. We added relative content in the result section on page 12.

13) Lines 498-506: In the section detailing TE analysis around the STE3.2-3 and STE3.2-2 loci, I have some points and suggestions to enhance clarity and comparability of the presented data. First, the current format of presenting Fig 6C and S30B separately makes direct comparison challenging. I suggest either placing these figures together or, alternatively, generating a plot that highlights the TE ratio or differences between STE3.2-3 and STE3.2-2. Secondly, the 'empty' portions of the bars in Fig 6C related to LTRs are not immediately clear and should be explained. Third, regarding the quantification of LTR Ty3 TE Family in Fig 6C, the text mentions that Pt 20QLD87 has the highest coverage of the LTR Ty3 TE family (Ty3_Pt_STE3.2-3), as depicted in Fig 6D. However, it's not evident from Fig 6C how this conclusion is drawn, as the coverage of this specific TE family doesn't seem to be separately quantified in that figure. Could you elucidate how this conclusion was reached or consider revising Fig 6C to explicitly demonstrate the coverage of Ty3_Pt_STE3.2-3?

Author's Response: Thank you so much for your suggestions, we now include S30B in Fig 6C. The empty white portion of the bars represent the coverage of Ty3_Pt_STE3.2-3, we added the description in the legend of Fig 6. Apologies for missing this description in a previous version.

14) I have noticed that the supplementary figures in the provided PDF are of generally low resolution. It's possible that this issue might have arisen due to the PDF conversion process during submission, but please double-check.

Author's Response: We apologize for this mistake, we fixed this issue in our latest supplementary file.

Response to reviewer 3's comments:

Reviewer #3: In this study the author investigated the structure, evolution and function of mating type loci in several rust fungi using publicly available dataset of genomes and expression data analysis.
I found that overall the authors addressed many comments of the reviewers on the previously submitted version and that this version of the manuscript is very interesting.

Author's Response: We thank the reviewer for the thoughtful comments and positive feedback. We are very happy about the excellent review process that really improved our manuscript.

My major comments about this new version are:

-over all the manuscript, I noted inconsistencies between abbreviations for the PR locus (Pra; PR; PRA…); it is unclear if there are differences between these abbreviations;

Author's Response: Thank you so much for pointing out this issue, we already adjusted this in our revised manuscript. '*Pra*' as genes, 'PR locus' as locus and 'PRA' referred to proteins. We refer to comments to reviewer 1 and 2 that clarified these issues further.

-Polymorphism within population between the two PR alleles seems low to me for a region that has likely recombination suppression (L444:one single SNP); it doesn't seem consistent with the dS value computed or I misinterpreted this part of the result.
Did the author use the denovo genome approach to check the polymorphism at PR loci as they did at HD loci?

Author's Response:

(1) We would like to address a potential point of confusion regarding this part: the alignment of amino acid sequences and SNPs detected by mapping short reads to reference genomes are comparing the same *Pra* allele within populations (*STE3.2-3* only compared to *STE3.2-3* allele variants and *STE3.2-2* only compared to *STE3.2-3* allele variants). The ds value plot compares *STE3.2-3* to *STE3.2-2.* The high ds values are caused by recombination suppression between the two alleles (*STE3.2-2* and *STE3.2-3*).

(2) It was not necessary to perform de novo assembly of Pra alleles as these are highly conserved in the populations and short-reads map very well against each allele (*STE3.2-2* or *STE3.2-3*).

-It is unclear if the hypothesis of a role in MAT genes for regulation of nuclear pairing is a new hypothesis or rely on previous observations (two references are cited L145; in the discussion, it appears as a new hypothesis) L678: indications could be given on how to further test this hypothesis.

Author's Response: It is hard to say that this is strictly speaking a new hypothesis considering the large body of work of *MAT* genes. We think it is not necessary, nor advisable to extend the manuscript even further. We hence decided against extending this discussion section.

-I had difficulties to understand the last part of the results about "shared nuclear haplotypes" and what the main conclusion of the author was. maybe a schema would help or clear conclusions.

Author's Response: We think it is not necessary, nor advisable to extend the manuscript even further. We hence decided against extending this discussion section and adding another figure.

-the text overall could be shorten (synteny analyses appear several times in the method, the part about STE3.2-1 loci could be merged in the result part)

Author's Response: Thank you for the suggestion, we have combined the method section of thee different synteny analyses into one paragraph from L804 to L810 and merged STE3.2-1 locus result part into one from L534 to L545 on page 23. In addition, the manuscript has now proofread by two native English speakers who are experts in the field. I hope you appreciate the improvements as much as we do.

Minor comments:
-L35: unclear what "genetic features" refers to

Author's Response: We have rewritten this sentence on L35 of page 2.

-L40: unclear what "allele status" refers to; bi-allelic or multiallelic

Author's Response: We have rewritten this sentence as 'allele diversity' on L40 of page 2.

-L64: states it is an example

Author's Response: We value the reviewers comment and decided to keep this sentence as is.

-L79: the model cited doesn't states that outcrossing mating system is necessary in the model; the model may work under inbreeding or automixis.

Author's Response: We rewrote and clarified this section.

-L84: cite Basidiomycota examples as they are more relevant to your study: Agaricus, Ustilago, Cryptococcus…

Author's Response: Thank you for the suggestion, we have added citation of *Cryptococcus*, *Ustilago hordei* and *Agaricus bisporus* on L87 and L88 of page 4.

-L120 : what is known about the PR locus? It is explained L155, but could be explained here

Author's Response: Thank you for the suggestion, we now moved the introduction of PR locus earlier from L91 to L96.

-L125: the term evolutionary strata needs to be defined

Author's Response: Thank you for the suggestion, we removed the term evolutionary strata here.

-L143: unclear why the term “rudimentary” means

Author’s Response: We have removed the word ‘rudimentary’ here and rewritten this sentence on L150 of page 7.

-L147-148: indicate on what evidence rely previous studies

Author’s Response: We addressed this issue accordingly.

-L195: use “degeneration” instead of “deterioration”

Author’s Response: Thank you for the suggestion, we now use ‘degeneration’ instead of 'deterioration’ for L203 of page 9.

-L617 : no s at model if only one is cited

Author’s Response: Thank you for pointing out this issue, we have corrected this issue accordingly on L644 of page 27.

-L625: It is unclear to me “the locus is extended” ; do the author mean “the region with signal of recombination suppression around the PR locus”? how did the author define this region precisely ?

Author’s Response: Thank you for pointing out this issue. We defined both loci more explicitly in accordance with accepted standards in the community e.g. Maia et al 2015.

For the HD locus see L342 and 343 on page 15: “We defined the HD locus as the DNA fragment which includes bW-HD1, bE-HD2, and the DNA sequence in between.”

For PR locus see L395 and 396 on page 17: “We investigated organization and synteny of the PR locus which we defined as the DNA fragments including Pra, mfa genes, and DNA sequences in between.”

-L630-631: TE composition and coverage at the order level: not very clear to me what the order means

Author’s Response: We used Repet pipeline for TE annotation which classified TEs based on Wicker’s TE classification system. See the comment to the editors feedback above for more details.
