# Supplementary material for: Genome biology and evolution of mating-type loci in four cereal rust fungi
Source: PLoS Genet. 2024 Mar 18;20(3):e1011207. doi: 10.1371/journal.pgen.1011207 (PMC10977897; doi:10.1371/journal.pgen.1011207)
Supplement: Attachment — Submitted filename: 20240229_ResponseToReviewer.pdf [file pgen.1011207.s042.pdf]

Response to editors and reviewers' comments for "*Genome biology and evolution of mating type loci in four cereal rust fungi*" (PGENETICS-D-23-00971R1)

The Authors' response is provided in blue and bold.

Response to editors' comments:

The manuscript has been much improved. However, there remains multiple occurrence of awkward writing, including formulations already highlighted as incorrect in the previous rounds of reviews, as highlighted below.

Authors' response: We thank the thoughtful review and valuable feedback.

-L4, L29, L213: mating-type loci

Authors' response: Thank you for pointing out these issues, we have fixed them accordingly on L4, L29 and L213.

-L33/ as already said in the previous rounds of reviews, it's not mating that is tetrapolar but the mating compatibility system

Authors' response: We fixed this issue by using 'tetrapolar mating compatibility system' instead.

-L41: we confirm that

Authors' response: We addressed this issue by adding 'that' on L41.

-L38: deleter extensive

Authors' response: We removed 'delete' according to this suggestion.

-L44: delete « of Puccinia » or « pathogenic Puccina species »

Authors' response: Thank you for the suggestion, we decided to use 'pathogenic *Puccinia* species' on L44.

-L46: delete « from sexual reproduction or somatic recombination » it does not make sense here

Authors' response: Thank you very much for your comment, we considered your request and decided to keep the phrase as part of *our* abstract and adjusted it to 'generated by sexual reproduction or somatic recombination,' from L45 to L46.

-L49: sexes are, not sex is (sex is sexual reproduction)

Authors' response: Thank you for pointing out this issue, we have fixed this sentence as 'Sexes in animals and some plants are determined by sex chromosomes' on L49.

-L50: replace « allele divs » by « features », this sentence makes no sense

Authors' response: We have substituted 'allele divs' with 'features' for L50.

-L57-58, L210-211, 725-727: this is too far-fetched

Authors' response: We considered this comment of the editor, however we disagree with the comment and therefore decided to keep this important thought about virulence evolution. We have discussed this concept with several leading experts in rust genome biology and evolution who all agreed that this is an important message of our manuscript. Any thoughtful reader will be able to decide for themself if they agree or disagree.

-L61: delete the ' signs ; delete loci or chromosome

Authors' response: We are surprised by this comment because the editor suggested to add ' signs during the initial review #1 as follows "-L60: I would rather say "sex and mating-type" **". We now decided to remove the ' signs but keep "loci or chromosome" for clarity reasons.**

-L71, 78, 80: delete « or new TE insertions », it is unclear how this can suppress recombination here

Authors' response: We deleted 'or new TE insertions' on L71 and L78 accordingly.

-L72-76: this is still very awkwardly explained and makes no sense in the present state.. replace by : « Instead, mathematical modelling and stochastic simulations suggest that non-recombining DNA fragments, caused for example by inversions, can be fixed solely due to the presence of deleterious mutations in genomes. Non-recombining fragments are beneficial if they carry fewer deleterious mutations than average and can increase in frequency. However, when becoming frequenct, their (few) recessive deleterious mutations will be exposed and selected against,

preventing fixation. They can fix at permanent biallelic, heterozygous loci due to their sheltering of deleterious mutations (2). »

Authors' response: Thank you so much for the detailed suggestion, we have rephrased this paragraph based on your suggestion.

-L106, 108 and elsewhere: replace « mating behavior » by « mating compatibility system »

Authors' response: We have replaced 'mating behavior' with 'mating compatibility system' throughout the manuscript according to this suggestion.

-L113: bipolar mating compatibility, not behavior

Authors' response: We are now using 'bipolar mating compatibility system' on L112.

-L115, 120, 160, 214, 215, 592 and elsewhere: tetrapolar mating compatibility, not behavior (you say L116 outcrossing behavior, which is here correct and shows the confusion around the term mating behavior in the crrent state)

Authors' response: We replaced mating behavior with mating compatibility system based on this suggestion.

-L117: compatibility odds

Authors' response: Thank you for pointing out this issue, we added 'odds' after 'compatibility' on L117.

-L157: the mating compatibility system (compatibility would be between two cells)

Authors' response: We have fixed this issue on L157.

-L179: these studies did not « open » gaps, they brought insights and left gaps, or gaps remain after these studies

Authors' response: Thank you so much for the suggestion, we fixed this sentence to 'These initial studies brought insights and left several outstanding knowledge gaps for MAT loci in rust fungi' on L179.

-L207: at least three ? or is the fourth one multi-allelic ?

Authors' response: We adjusted the sentence in question to clarify it further.

-Please stick to the accepted Latin abbreviation system for species names

Authors' response: We appreciate the comment, however it is unclear what the specific concern of the editor is in this case. We prefer to keep the short abbreviations of *Pca*, *Pgt*, *Pt,* and *Pst* as they are very commonly used in the community and greatly improve the readability of the manuscript.

-L362: replace « aberrant patterns of gene or transposon density » by « particular patterns of gene or transposon density » (enriched TE patterns are not « aberrant » under recombination suppression)

Authors' response: Thank you for pointing out, we have replaced 'aberrant patterns of gene or transposon density' with 'particular patterns of gene or transposon density' on L363.

-L431: « whole-genome »

Authors' response: Thank you for pointing out this issue, we have fixed it on L432 and addressed it elsewhere in the manuscript.

-L432: genome singular as there is « respective » before

Authors' response: Thank you for pointing out this issue, we have made genome singular on L433.

-L474: « This species rank variation » : it is the level that is ranked not the variation itself

Authors' response: We have replaced 'rank' by using 'level' on L475.

-L476, 497 : level instead of rank

Authors' response: We used 'level' instead of 'rank' for L477 and L498 based on this comment.

-L543 : « the two MAT genes » : it sounds as you already know the two MAT genes while in the

sentence you aim to investigate a potential mating-type function for a third one, so this sounds unclear, reformulate

Authors' response: We adjusted the end of the sentence in L 546 to clarify our point.

-L593 : as already said, the causality is reverse : it's not that outcrossing benefits from new alleles (which is wrong, group selection reasonning), but that, under outcrossing, new, rare allleles are selected for, this is completely different.

Authors' response: We decided to delete this sentence.

-L601 : unclear formulation, add « each » after species ? or is the number all four species pooled ?

Authors' response: We adjusted the sentence as follows.

"We investigated the allelic diversity of bW-HD1 and bE-HD2 in 12–15 additional isolates for each cereal rust fungal species. For each species these were sampled in a way to provide the best available representation of unique genotypes from global populations"

-L618 : this is also the case in Microbotryum, see Michael Hood's studies

Authors' response: Thank you for pointing out this, we added Microbotryum species here and cited Michael Hood's work.

-L650 : « exact trends » seems contradictory, replace by patterns

Authors' response: We replaced "trends" with "patterns" on L 651.

-L688 : why would they have other functions ? Delete this sentence, or rephrase saying there is no evidence of additional functions

Authors' response: We considered this comment carefully and disagree with the evaluation of the editor. We decided to keep this sentence as is in *our* manuscript.
