# Supplementary material for: Genome biology and evolution of mating-type loci in four cereal rust fungi
Source: PLoS Genet. 2024 Mar 18;20(3):e1011207. doi: 10.1371/journal.pgen.1011207 (PMC10977897; doi:10.1371/journal.pgen.1011207)
Supplement: Attachment — Submitted filename: 20240108_ResponseToEditorAndReviewers.docx [file pgen.1011207.s040.docx]

**Response to editors and reviewers comments for “*Genome biology and evolution of mating type loci in four cereal rust fungi*” (PGENETICS-D-23-00971R1)**

**The author’s response is provided in blue and bold.**

**We are very grateful for the thoughtful feedback and critical suggestions by the editor and the external reviewers. We provide on overarching reply in the cover letter. Our detailed point by point replies can be found below.**

**Response to editor’s comments:**

The manuscript has been evaluated by three referees, who agree that this manuscript on the mating-type loci of rust fungi could be of interest for PLoS Genetics, providing valuable insights into mating-type loci in rust fungi. Despite the positive assets acknowledged by the referees, however, serious concerns were raised, in particular about methods, generalization from one isolate to species, unwarranted conclusions about HD gene recombination, language, format, clarity of the text and figures, lack of citations of previous works, the inclusion of a single PR allele in gene genealogies, which does not allow analysing trans-specific polymorphism, lack of discussion on the drivers of recombination suppression, lack of information on the size of recombination suppression, lack of analyses of polymorphism data and of recombination suppression at HD genes, and lack of data availability. I have to agree with these concerns. The manuscript is not very well written, many sentences are unclear or make no sense, are awkward or with wrong syntax. The introduction includes irrelevant information about resistance genes but lack essential ones, and the predictions at the end of the introduction seem ad hoc, while more interesting one could be stated. Some examples are highlighted below and by referees but the text needs to be edited all along the manuscript, and the figures improved. I would encourage resubmission only if you are able to revise the manuscript along these lines and add the corresponding analyses. The referees also provide a list of excellent additional suggestions and questions, which should also be addressed. Please also be sure to cite previous works on mating-type loci in rusts.
**Author’s Response:**

**Please refer to the cover letter for the overarching reply to the editor’s comments provided in this paragraph.**

E.1. -Please number the pages

**Author’s Response: We have numbered the pages.**

-L29: It's not the loci that give rise to recombination suppression, they undergo recombination suppression; It's not necessarily the linkage either.

**Author’s Response: We have addressed this issue and updated the abstract and introduction accordingly.**

-L60: I would rather say "sex and mating-type”

**Author’s Response: We have addressed this issue on the page 3 accordingly.**

-L66-68, L516 : it’s not really « initial sheltering » but rather « a combination of an initial advantage of carrying fewer deleterious mutations than average and the sheltering of these few deleterious mutations when increasing in frequency ». I would also highlight here the relevant prediction made in Jay et al 2022 that recombination suppression extension should only occur in dikaryotic species, and only around biallelic loci not multi-allelic ones. It would thus fits the findings that it occurs around the biallelic PR locus and not the multiallelic HD locus, and come back to this in the discussion.

**Author’s Response: Thank you very much for these excellent suggestions. We have followed the provided suggestions. We adapted the logic flow and arguments made in the updated manuscript accordingly.**

-L90, L93 : advantageous

**Author’s Response: We have corrected this error on page 5.**

-L93 : awkward sentence. It’s not that « multi allelism » is advantagous (this is wrong group selection reasonning), but that any new, rare allele is advantageous, which leads to multi-allelism ; this reasonnning is very different.

**Author’s Response: We have included more detail description around multiallelism on page 5-6.**

-L98 : awkward sentence : the mating system is selfing versus outcrossing while the mating compatibility system is bipolar or tetrapolar, this is very different and the relationships between the two systems is not simple. A tetrapolar compatibility system does not decrease inbreeding (you can always self), but it is less advantageous under inbreeding, this is very different. I would delete this whole paragraph, not relevant to the study anyway.

**Author’s Response: Thank you very much for these detailed suggestions we updated the manuscript accordingly.**

-L105-132 : delete also this paragraph, it’s not relevant to the study and there are several awkward sentences (for example an isolate is not « adapted », it’s populations that adapt not individuals). Just give the minimal information on the role of MAT genes in the life cycle.

**Author’s Response: Thank you very much for these detailed suggestions we updated the manuscript accordingly.**

-L133 : why « likely » ? based on what ?

**Author’s Response: We have explained this more greater detail on page 7.**

-L136 : MAT loci not alleles.

**Author’s Response: We have addressed this issue in the manuscript.**

-L143-150 : awkward predictions, we don’t understand why, they seem like ad hoc, after you knew the results. I would rather explain predictions about recombination suppression around biallelic loci and not multiallelic ones as explained above based on Jay et al. 2022.

**Author’s Response: Thank you very much for these excellent suggestions. We adapted the logic flow and arguments made in the updated manuscript accordingly. Indeed, we only observe strong signals of suppression of recombination and genomic degeneration for clearly biallelic *MAT* loci.**

-avoid abbreviations, and in particular stick to conventional Latin abbreviations for species names.

**Author’s Response: We have addressed this issue in the manuscript. We clearly explain abbreviations and stick to conventional Latin abbreviations whenever possible.**

-Table legends are too short, remove capitals, explain abbreviations, explain all lines and columns

**Author’s Response: Thank you for the suggestion, we have addressed this issue by adding more information in caption (a.k.a. figure legends). We revised all figure and table legends including in the supplement.**

-L184-186 : awkward sentence

**Author’s Response: We have deleted and rewritten this sentence on page 10.**

-L201 : « fully conserved » is unclear : present ? at the same location ? identical sequence ?

**Author’s Response: We have deleted and rewritten this sentence on page 10.**

-L205 : « composition » is unclear : identical sequence ?

**Author’s Response: We have replaced ‘composition’ with ‘sequence’.**

-L218 : see Petit et al (2012) Evolution 66: 3519–3533.

**Author’s Response: We have included Petit et al 2012 in the citations as appropriate.**

-L222, L252, L522 : explain better what trans-specific polymorphism means and refer to the first paper on this on the PR gene : Devier et al 2009 Genetics 181:209-223. Predictions about this in the introduction would be better than the predictions currently at the end of the introduction.

**Author’s Response: We have extended our explanation of trans-specific polymorphism in introduction and cited Devier et al 2009 accordingly.**

-L232 : either rather than both

**Author’s Response: We have rewritten this sentence on page 12.**

-L235 : unclear what is your initial hypothesis and why. This whole page is hard to follow, having the last sentence at the beginning would help (but see referees’ comments on this inference, likely unreliable due to low bootstraps)

**Author’s Response: Thank you very much for these excellent suggestions. We adapted the logic flow and arguments made in the updated manuscript accordingly.**

-L261 : run a formal test of congruence

**Author’s Response: We preformed AU-test for testing congruence of *bW-HD1* and *bE-HD2* for each species separately. We updated the manuscript accordingly in the results and methods section.**

-L267 : these are not phylogenetic trees but gene genealogies (phylogenies are for species)

**Author’s Response: We have updated the manuscript accordingly.**

-L271 : alleles and not genes

**Author’s Response: We have corrected figure captions based on this comment.**

-L300 HD locus

**Author’s Response: We have fixed this sentence on page 14.**

-L310 and elsewhere : S in dS should be subscript

**Author’s Response: Thanks for pointing out this issue, we corrected ds to d_s_ in the whole manuscript.**

-L326 : delete « pair » and « are »

**Author’s Response: We have fixed this issue in captions of Figs 4-5.**

-L397 : conservation is unclear : in synteny ? sequence ? within instead of « of each »

**Author’s Response: We have fixed this issue and added additional explanations on page 17.**

-L398 : any conserved

**Author’s Response: We have fixed this issue on page 17.**

-L404 : awkward wording

**Author’s Response: We have addressed this issue.**

-L401 : ancestral to what ?

**Author’s Response: We have rewritten this sentence on page 13.**

-L415 : awkward wording

**Author’s Response: We have rewritten this sentence on page 18.**

-L428 : no plural at polymorphism

**Author’s Response: We have corrected this on page 18.**

-L439-440 : this makes no sense.

**Author’s Response: We adapted the logic flow and arguments made in the updated manuscript accordingly.**

-L445-449 : unclear ; what is the « status » ? conserved means identical sequences ?

**Author’s Response: We clarified the usage in the updated manuscript.**

-L453 : I don’t think this is true beyond this particular species in the given reference

**Author’s Response: We have rewritten this sentence on page 22, removed the specific example, and added more relevant citations.**

-L457 : delete significant

**Author’s Response: We have fixed this issue on page 23.**

-L460 : describe is not the right term here

**Author’s Response: We have rewritten this sentence on page 22.**

-L463, L466 : we’re lost with the jargon here, these terms should be explained in the introdcution instead of the long irrelevant paragraph on R genes. Explain whether these findings are consistent with exectations given the life cycle and where the MAT genes act.

**Author’s Response: We adapted the logic flow and arguments made in the updated manuscript accordingly. We used the introduction to explain the necessary details of cereal rust life cycles and the known or hypothesized roles of *MAT* genes. Unfortunately, we don’t know much about *MAT* genes in rust fungi. The new introduction makes it easier to follow the presented RNAseq analysis and its logic.**

-L482 : mating compatibility not sexual reproduction, this is very different

**Author’s Response: We have addressed this in the updated manuscript.**

-L487 : make it clear whether or not other genes are trapped into the non-recombining region

**Author’s Response: We have addressed this in the updated manuscript.**

-L489 : the PR locus

**Author’s Response: We have addressed this in the updated manuscript.**

-L491 : the hypothesis

**Author’s Response: We have addressed this in the updated manuscript.**

-L492 : delete parasexual reproduction, this has not been defined nor shown.

**Author’s Response: We have addressed this in the updated manuscript by deleting parasexual reproduction.**

-L494 : mating is not tetrapolar, mating compatibility is, this is very different

**Author’s Response: We have addressed this in the updated manuscript.**

-L506 : why mitosis activity ?

**Author’s Response: We have rewritten in the updated manuscript.**

-L508 : delete « between haplotypes but » and begin a new sentence with « Chromosomes… »

**Author’s Response: We have addressed this in the updated manuscript.**

-L519 : likely caused

**Author’s Response: We have addressed this in the updated manuscript.**

-L529 : awkward sentence, the causal « because « sounds odd : it’s not the cause but the observation allowing the inference

**Author’s Response: We have addressed this in the updated manuscript.**

-L532 : see referees’ concerns about this, I agree this should not work and is poorly supported.

**Author’s Response: We have addressed this in the updated manuscript.**

-L537 : it’s not consistent but similar

**Author’s Response: We have addressed this in the updated manuscript.**

-L545-6 : this is wrong group selection reasoning : selection does not work for the good of the species

**Author’s Response: We have rewritten this sentences in the updated manuscript.**

-L548 : pheromone receptor gene and singular or locus ?

**Author’s Response: We have addressed this in the updated manuscript.**

-550 : at each ?

**Author’s Response: We have addressed this in the updated manuscript.**

-L556 : unclear

**Author’s Response: We have addressed this in the updated manuscript.**

-L558-560 : unclear and unconvincing, delete

**Author’s Response: We have addressed this in the updated manuscript.**

-L566 : unclear ; of different mating types ?

**Author’s Response: We have addressed this in the updated manuscript.**

-L573-3 : this should be explained in the introduction instead of the long digression about R genes

**Author’s Response: We have addressed this in the updated manuscript.**

-L576 : unclear ; is a verb missing ?

**Author’s Response: We have addressed this in the updated manuscript.**

-L582-596 : this makes no sense and is irelevant, delete

**Author’s Response: We have addressed this in the updated manuscript.**

-L601 : demete between species, pleonasm
**Author’s Response: We have addressed this in the updated manuscript.**

**Response to reviewer 1’s comments**

Reviewer #1: In this study, the group of B. Schwessinger made use of several Puccinia striiformis f.sp tritici (Pst) genome assemblies which they generated previously; they were among the first pioneering the proper haplophasing of the two haploid genomes residing in the two haploid nuclei in each urediniospore (the source of gDNA used for sequencing projects). Here, they compared these to genomes from three other Puccinia species pathogenic to cereals (mostly wheat), and whose haplophased genomes were recently published. They present the organization and variation of the MAT loci (HD and PRA/mfa) among these species/ forma speciales. Though the various mating-type genes (HD and PRA/mfa) have been reported on elsewhere in a few studies including their likely tetrapolar arrangement in Puccinia species, there has not yet been done a more in depth comparison of their arrangement and genomic neighborhood in various Puccinia species (the four presented here). This is becoming feasible only recently because of the above-mentioned haplophased assemblies. From the comparisons, they infer evolutionary principles of the MAT loci for these rust fungi.

Claims:
- the tetrapolar arrangement of the HD and PRA/mfa mating-type loci in all four species; has been substantiated
- similar, little degeneration around HD loci is found when comparing haplotype regions, as to other genomic regions as an indication of low recombination suppression; has been substantiated
- PRA/mfa mating-type locus much more degenerated among haplotypes, surrounded by higher levels of repetitive DNA (transposable elements or TEs, and low gene density / “gene desserts”) as other genome regions, as an indication of recombination suppression preceding speciation; substantiated to a certain degree but see comments below on TEs
- tried to locate centromers and link these to mat loci; see comments below
- the HD genes are multiallelic “highly polymorphic” within various species isolates; see comments below
- the PRA locus is biallelic with one allele in each haplotype (STE3.2-2 and STE3.2-3) in the sampled species; substantiated. Potential polymorphisms may indicate more allelic variants, particularly in Pgt (correlating with potential three allelic PRA genes?); see comments below
- use of publicly available transcriptome data for the four species indicates both MAT loci are expressed during the asexual infection cycle and higher expression is seen in later stages of the infection (very few data exists on expression during the sexual stages on alternate hosts); tentative, see comments below.

Overall, a nice assessment of MAT loci in this group of fungi, but some issues would need to be resolved before statements / claims can be made.

**Author’s Response: We thank the reviewer for the nice summary of our manuscript and the critical feedback.**

Line 350-351: “Composition of transposons vary across species, DNA transposons dominated the HD locus of Pst134E but HD loci of other rust fungi had higher coverage of RNA transposons (S9 Fig).”
And Line 400-401: “Pst 210 and Pt 76 showed strong signals of accumulation of independent transposable element families in each nuclear haplotype, which indicates invasion by TEs since cereal rusts shared a most recent common ancestor.”
- As the supplementary figure only contains single representatives of the species, can species-wide conclusions be drawn? Could you provide more explanation on the families of TEs and how the results were generated for figure S9?

**Author’s Response: We have now updated the manuscript to address these questions. First we extended our analysis of TEs around the *HD* and *PR* loci (S12 Fig). We also extend the analysis of TEs around *HD* and *PR* loci in three additional phased chromosome scale assemblies for *P. triticina*. Overall, the TE composition is very consistent between copies of the different *HD* and *PR* alleles within this species. The TE coverage though is variable between copies of *PR* alleles. We discuss these new findings and general considerations around inferences at the species level in the manuscript. We point out that more phased and chromosome scale assemblies are necessary for these cereal rust fungi.**

Lines 568-575: “detailed transcriptomic analysis of MAT genes now gives an initial indication that these genes are important for nuclear pairing in the asexual stage, which is consistent with earlier preliminary reports in Pt (21) and M. larici-populina (62, 63). Our analysis suggests that MAT genes are expressed in asexual life cycle stages of cereal rust fungi. In our detailed transcriptomic analysis, we found both HD and PRA genes were upregulated during the infection of the asexual host with a peak at final infection stages that align with spore production and dispersal HD genes are known to control post-mating growth, such as control development of fruiting-body in some Ascomycota and Basidiomycota species (64).“ Also Figure 6.
- Does the up-regulation of these genes really warrant this conclusion? Moreover, from figure 6C, bE2-HD2 does not change in expression. Based on the plot, between 24h and 48h, there is a lot of variation for the bW1-HD1, bW2-HD1, and Ste3.2-3 transcripts, and using the median point no increase of expression when compared to 5days post infection, is there an explanation?

**Author’s Response: We now updated the manuscript to phrase our hypotheses more as such. For former figure 6C (now figure 7C), one of *bE-HD2* expression didn’t change because the specific allele of *Pst 87/66* isolate was not well reflected in the *Pst 134E* gene set used for RNAseq quantification. We addressed this problem by replacing the *bW-HD1* and *bE-HD2* cds with those from *Pst 87/66* to accurately reflect the expression of these genes in this dataset. This correction clearly shows that both *bE-HD2* are upregulated Fig 7 and S36 Fig.**

- Is there a lot of variation between genes at the early time points? For early time points, is there is a possibility that there is not enough fungal biomass to get meaningful number of reads, or depth, to make these inferences?

**Author’s Response: The reviewer is correct that fungal biomass is low early on during infection. However, our detailed analysis as presented in the updated manuscript (e.g. expression of house keeping genes, validation of RNAseq datasets) supports our observations made.**

- Is it possible to get information from kallisto or downstream EdgeR of how many genes have counts at the early time points relative to spore or late sample times. And do you have sufficient counts at these early time points for differential expression analysis? Another quick method would be to look at housekeeping genes throughout time points or genes that don’t change.

**Author’s Response: These are excellent suggestions. We have deposit a table of fungal read counts as part of our Dryad repository (https://datadryad.org/stash/share/XCyaQ2j8q8yI28amg3ct1WplAOlh-yTUHfaz_jyDYK4) linked to the manuscript. We also provide expression analysis of two housekeeping genes per species in the updated manuscript.**

- Also, there is / we could not find any reference to parameters used with EdgeR within the github, only deseq2, the parameters and methodology would be good to see.
**Author’s Response: We now have completely updated the methods section and provide a detailed Github repository (https://github.com/ZhenyanLuo/codes-used-for-mating-type/) with all analysis code used in the manuscript.**

Issues with Methods.
Lines 631 -635: “Estimation of centromeric regions…. Hi-C reads of Pca 203 and Pt 76 were mapped back to corresponding diploid genome assembly with Juicer 1.6 (71) and processed with 3d-dna (72) pipeline. Juicerbox 1.8.8 (71) was used to visualize Hi-C maps and manually estimate centromeric regions by identifying strong interaction signals.”
- Please, include the Hi-C contact maps used for centromeric identification. As discussed with respect to the PR locus, this region is marked by repeats and transposable elements which would affect the mapping of Hi-C reads, how can you be confident where the centromere is? Is there a centromeric specific sequence motif between the analyzed isolates like found in other fungi?

**Author’s Response: We now include S22-25 Figs for Hi-C heatmaps of *Pca 203* and *Pt 76*, centromeric of *Pgt 21-0* (Sperschneider et al. 2021) and *Pst 134E* (Schwessinger et al. 2022) have been published previously. Identifying centromeric regions from Hi-C heatmaps are a common practice and also has been automated with bioinformatics pipelines such as HiCAT (Gao, et al. 2023).**

- I would if it was mentioned at the beginning of the methods that specific scripts and parameters used in the study are in the attached git repo. Also, include how REPET/TEdenovo was done in the main methods, as transposable elements are in the main figures.
**Author’s Response: We have now completely updated the methods section. All analytic code used for the manuscript can also now be found on our associated Github repository https://github.com/ZhenyanLuo/codes-used-for-mating-type/.**

Lines 658-664: “…before and after trimming. Bwa-mem2 (65) was used to map trimmed reads to reference genome, MarkDuplicates (Picard) 1.70 (92) was applied to remove PCR-generated duplicates. Qualimap 2 (93) was used to check mapping coverage and quality. Freebayes-parallel 1.3.6 (94, 95) was processed to detect variants, samtools 1.12 (96) was used to filter out regions of interest. The Ensembl Variant Effect Predictor (VEP) 88.9 (97) was used to annotate sequence variants. Bcftools 1.12 (96) was used to generate consensus sequence, nucleotide and protein alignments were generated and visualized by Geneious Prime v. 2022.1.1 (66).”
- Could you provide coverage plots for each of the genes for all the isolates to ensure that the genes are fully covered by short reads, and that there is sufficient coverage for consensus sequences to be created from the variants.

**Author’s Response: We now provide screenshots of the *HD* and *Pra* loci when mapping Illumina short-read datasets against the whole genome (S17-20 Figs and S22-25 Figs). These show variable coverage for *HD* genes and full coverage of *Pra* genes. We therefore developed a novel *de novo* reconstruction approach for *HD* alleles directly from Illumina short-read datasets. We validated this method using *Pca 203* short-read data and compared the reconstructed alleles against the reference (S21 Figs). The *de novo* reconstruction of *HD* alleles showed that *HD* loci are multiallelic in cereal rust fungi. Our novel approach will be useful for others interested in fungal community.**

- Are there regions where reads are not aligning due to differences? This has also implications for the (HD) allele diversity you claim.

**Author’s Response: We deposited the mapping graphs in S17-20 Figs and S22-25 Figs, these graphs show coverage of reads after removing duplicated mapping and low-quality mapping (<40). *HD* alleles have low coverage in several isolates due to variations. *Pra* genes in all cases have good coverage in S22-25 Figs. We therefore developed a novel *de novo* reconstruction approach for *HD* alleles directly from Illumina short-read datasets. We validated this method using *Pca 203* short-read data and compared the reconstructed alleles against the reference (S25 Fig). The *de novo* reconstruction of *HD* alleles showed that *HD* loci are multiallelic in cereal rust fungi. Our novel approach will be useful for others interested in fungal community.**

Other issues and suggestions:
Line 76: …’ to govern nuclear compatibility and inbreeding within populations’; maybe better: ‘… to govern mate selection within populations and nuclear compatibility’? It is not solely for “inbreeding”.
**Author’s Response: We have addressed this in the updated manuscript.**

Line 84: maybe add ‘..which are outwardly transcribed in opposite directions.’?
**Author’s Response: We have addressed this in the updated manuscript.**

Line 85: ‘cascade’, one word
**Author’s Response: We have fixed this mistake on page 5.**

Line 87: ‘…and the involvement of one or both loci in mating’?? Better: ‘Different segregation patterns of these mating-type loci define the terms bipolar or tetrapolar mate compatibility.’
**Author’s Response: We have addressed this in the updated manuscript.**

Line Suggested: ‘…are physically linked and hence only one MAT locus controls mate compatibility.’
**Author’s Response: We have addressed this in the updated manuscript.**

Line 90: advantageous
**Author’s Response: We have addressed this in the updated manuscript.**

Line 98: The tetrapolar..
**Author’s Response: We have addressed this in the updated manuscript.**

Line 101-104: A very nice new example is: Coelho et al., 2023: doi:10.1073/pnas.2305094120
**Author’s Response: We now have included this manuscript as example in the introduction.**

Line 113: ‘is prevented by management strategies that remove the sexual host e.g. Berberis’. I think, though this has helped in the past, the fact that the alternate host(s) may not be available in that region of production of the primary host, may be more important?
**Author’s Response: We have addressed this in the updated manuscript.**

Line 118: ‘it poses serious risks’
**Author’s Response: We have addressed this in the updated manuscript.**

Line 122: Suggestion: ‘As alternatives to sexual reproduction and gene assorting, somatic hybridisation in which nuclei are exchanged during hyphal interactions can increase genotypic diversity including the generation of novel effector complements (29, 30).’
**Author’s Response: We have addressed this in the updated manuscript.**

Line 126: ref 31 is cited but this publication is based on a seriously flawed Pt genome assembly (this publication should be retracted and not cited). If Pt genome assembly information is needed, a better resource is a preprint by Sperschneider et al. bioRxiv preprint [https://doi.org/10.1101/2022.11.28.518271](https://aus01.safelinks.protection.outlook.com/?url=https%3A%2F%2Fdoi.org%2F10.1101%2F2022.11.28.518271&data=05%7C01%7CZhenyan.Luo%40anu.edu.au%7Cf4271e4252b8425c781f08dbbfbd44d2%7Ce37d725cab5c46249ae5f0533e486437%7C0%7C0%7C638314592224101393%7CUnknown%7CTWFpbGZsb3d8eyJWIjoiMC4wLjAwMDAiLCJQIjoiV2luMzIiLCJBTiI6Ik1haWwiLCJXVCI6Mn0%3D%7C3000%7C%7C%7C&sdata=fNSUWWk7HH5VbjCxIOKFRGPpD2P4Vn5%2FiLsoHqAVGDQ%3D&reserved=0)

**Author’s Response: Thanks for these suggestions. We updated the citation. Our analysis is based on *P. triticina* genomes published in Sperschneider et al. 2023 and Li et al. 2022, and not the one in the previous reference 31.**

Line 146: delete ‘hypothesis’
**Author’s Response: We have rewritten this sentence on page 9.**

Line 157 – 159: “Here we made use of the four available chromosome-level genome assemblies of cereal rust fungi at the time of the study including Pca 203, Pst 134E, Pt 76 and Pgt 210 (Table 1). We included P. polysora f. sp. zeae (40) or Melampsora larici-populina as an outgroup.”
- It would be nice to see in the table which assemblies are complete/assembly information sizes etc.

**Author’s Response: We agree with this suggestion, the completeness of assembly information is in Table 1, and reference/query genes used are now included in Table 2.**

Line 169: ‘…that are thought to bind to’; this has not yet been proven, and is thought to be analogous to other, more-studied basidiomycete fungi….

**Author’s Response: We have rewritten this on page 11.**

Line 174 – 175: “In the case of Pst 104E, STE3.2-2 was absent from the genome assembly but we recovered its haplotype by mapping raw sequencing reads of Pst 104E to the Pst 134E phased chromosome scale genome assembly (34)”.
- Could you clarify what was done here? Did you map reads then assemble this region ? Or take the consensus of the mapped reads as STE3.2-2. ?

**Author’s Response: We mapped whole genome sequencing reads from *Pst 104E* against the reference of *Pst 134E* and then reconstructed *STE3.2-2* from the consensus of the mapped reads. This is similar to our analysis presented in now S25 Fig. We amended the text accordingly.**

Line 216: ‘…is also diverse…’

**Author’s Response: We have rewritten this on page 11-12.**

Line 392: ‘the PRA genes of Pst 134E were found to be ~164kbp from the centromere of chromosome 9A and ~473kbp from that of chromosome 9B, likely physically-linked to their centromeres’.
- to which figure is this referring, and are these typos? Those distances don’t look like “physically-linked’?!
**Author’s Response: We thank the reviewer for these suggestions. We now rephrased this section of the manuscript which refers to Fig 4.**

Line 317-318: “The information of centromere locations suggests that the HD loci are likely not directly linked to centromeres in all four species”.
- This was based on juicer hi-c contact plots, can they be included in the supplementary?

**Author’s Response: We included S22-25 Figs for Hi-C heatmaps of *Pca 203* and *Pt 76*, centromeric of *Pgt 21-0 Pgt 21-0* (Sperschneider et al. 2021) and *Pst 134E* (Schwessinger et al. 2022) have been published previously.**

Line 386-387: “Gene density dropped significantly around PRA genes with STE3.2-2/STE3.2-3 located within or adjacent to ‘gene deserts’ that extended over 1mbps in the case of Pst 134E.”
- Could this be a factor of current annotation limits as these regions contain large repetitive regions / transposons and normally these would be masked in conventional annotation pipelines? Do all gene annotations have RNA support, or vice versa, are there RNA reads mapping to areas where no genes have been “annotated”?

**Author’s Response: These are interesting thoughts and suggestions. However, it is outside of the scope to reannotate genomes, manual check for open reading frames around *PR* loci, or to check if RNAseq datasets map to open reading frames that are not TE related in these regions. This would be more part of follow on studies around TEs and genes surrounding the *PR* loci.**

Line 423: I do not see the Pca HD maps in this S14 figure to substantiate the text….?

**Author’s Response: In previous manuscript, since *HD* loci didn’t map well in *Pca*, we failed to generate consensus sequence. We now updated S17 Fig and S21 Fig for showing the multiallelic *bW-HD1* and *bE-HD2* in *Pca*. This new analysis is based on a novel de novo reconstruction approach of *HD* alleles directly from Illumina short-read datasets as outlined above.**

Line 424-430: “This is likely caused by the fact that isolates selected were mostly linked to sexual populations of Pca found in the USA (49). Hence we also used the most closely related available reference of each sample for short read mapping purposes including references from Pca 203 (50), Pca 12NC29 and Pca 12SD80 (49). This showed that the HD loci in Pca display high levels of polymorphisms including heterozygous SNPs, multiple unaligned regions and gaps, which suggests the HD loci present in our selected Pca isolates are not well reflected in the available references.”
- Would you expect this for the other isolates?
**Author’s Response: We now explain this in more detail in the update manuscript. Many *Pca* isolates come from sexually recombining population and hence they are expected to have the most diverse HD allele set. See page 18-19 for more details.**

Line 439-444: “We identified slightly more SNPs in STE3.2-2 and STE3.2-3 of Pt which we estimate to give rise to a maximum of four distinct haplotypes. Yet each predicted haplotype has only a very limited number of amino acid substitutions. Pgt was the most polymorphic at the STE3.2-2 and STE3.2-3 loci. We identified three haplotypes of STE3.2-2 with several amino acid changes. STE3.2-3 was the most polymorphic in Pgt including two isolates, TTTSK and UVPgt60, which contain potential nonsense mutations leading to pre-mature stop codons.”
- If these regions are marked by increase of repeats, what is the mapability of the short reads to these genes and loci?

**Author’s Response: Short reads mapped to the *Pra* genes have high Q-scores (>40) because *Pra* alleles are unique and conserved at the species level. This is shown in the S22-25 Figs which provide screen shots of short reads being mapped at the *PR* locus. Indeed, we reconstructed *Pra* copies from short read datasets filtering reads based on mapping quality greater 40. It is the surrounding repetitive regions that show poor mapping quality due to their repetitiveness. We do not use Illumina short-read datasets to analyse these regions.**

And, as mentioned at line 513-514: “and complete loss of synteny within and among species surrounding he PR loci”.
- Doesn't this suggest that there will be issues of mapping from other isolates to these regions if haplotypes of the same isolate show high heterozygosity for the PR loci as in Fig 4 B?
**Author’s Response: We refer the reviewer to the reply above. In addition, we now make use of three more fully phase chromosome scale genome assemblies of *P. triticina*. This shows that *PR* alleles are overall conserved despite some allele specific TEs extensions.**

Line 484: ‘two loci that control mate compatibility’; I think such statements (and in general throughout the manuscript, e.g. also lines 548-550) need to be presented as tentative since no functional molecular work has demonstrated this in the rusts. All based on analogous work in other basidiomycetes, primarily Ustilago and Cryptococcus.
**Author’s Response: We have addressed this in the updated manuscript.**

Line 485: “…HD loci are highly multiallelic”; again, overstated since only cursory, indirect data from a few isolates per species has been presented; see comment above on potential problems with mapability of (Illumina) short reads.
**Author’s Response: We thank the reviewer for pointing out this issue. Indeed, Illumina short-read datasets don’t map well for *HD* loci considering their high allelic diversity. The updated manuscript now provides a novel *de novo* reconstruction approach for *HD* alleles directly from Illumina short-read datasets. This approach does not rely on mapping against existing *HD* reference alleles. We refer to above replies and the methods section for details. We reconstructed between six and twelve *HD* alleles for each species. This is consistent with the ten reported *HD* alleles for *Pst* by Holden et al. 2023. We discuss this in greater detail in the manuscript and we think it is fair to say that the *HD* locus is multiallelic in cereal rust fungi.**

Line 519: “…likely be caused’; ‘…is likely caused’
**Author’s Response: We have rewritten this sentence.**

Lines 520-521: “Interestingly, the TE families surrounding PR loci are not shared within or among species.”
- Again, is this a valid claim when only looking at four species and single isolates from each?

**Author’s Response: We thank the reviewer for these suggestions. We now extended our analysis to three additional phased chromosome scale genome assemblies for *P. triticina*, which is the only cereal rust fungus with multiple reference grade public genomes at the moment. In these cases the TE composition, while not the coverage, was conserved for *PR* loci. This suggests that it might be possible to make general hypotheses for species looking at a single reference genome in case of *MAT* loci. Several publications of *MAT* loci in Microbotryum *sp.* also relied on single reference genomes to make inferences about these loci between species. Of course, this is not ideal, yet this is the best we can do given current absence of population wide phased chromosome scale reference genomes for cereal rust fungi. Of course, our current hypotheses might change with new datasets becoming available.**

Line 555 and on, Discussion on potential multiallelic PRA alleles in Pgt; there is precedence in Sporisorium reilianum: Schirawski et al. 2005 DOI:10.1128/EC.4.8.1317-1327.2005
**Author’s Response: We have cited Schirawski et al. 2005 on page 26.**

Line 564 – 566: “This is even though nuclei of the same mating type share the same cytoplasm during colonisation of the asexual host."
- I do not understand this comment. If it read “…sexual host...”, it would maybe make sense ((haploid) teliospores undergo another mitotic division so a dikaryotic cell / infection hypha directly penetrates the alternate host leaf / epidermal cell.

**Author’s Response: We addressed this issue by rewriting this sentence on page 28.**

Line 567: …is unknown; I don’t think that this preliminary transcript analysis is enough to prove that ‘… (MAT genes) are important for nuclear pairing in the asexual stage’. Some experimentation (Cuomo et al., 2017) suggests that they affect overall virulence…..
**Author’s Response: We have addressed this in the updated manuscript. We simplified the discussion on this topic and phrased it more tentatively.**

Line 574: ‘…and dispersal.’ Period
**Author’s Response: We have addressed this in the updated manuscript.**

Lines 582-586: “Individual nuclear genomes of rust fungi are entire linkage groups, with effector haplotype complements being tied to specific MAT haplotypes if somatic hybridisation occurs in the absence of parasexual reproduction. Hence, we can predict the possible combinations of effector complements that can arise by somatic hybridisation based on the knowledge to which MAT haplotypes they are linked to.”
- Doesn’t this suggest that there are only a few combinations based on haplotypes that can exist? Can this claim be made when there is a large amount of transposable elements? Are you confident there is no asexual mechanism for information exchange between haplotypes during somatic hybridization?
**Author’s Response: Indeed, this is the suggestion made by Sperschneider et al. 2023 Nature Microbiology for *P. triticina* on wheat. It is beyond the scope of this particular manuscript to test for information exchange between haplotypes during somatic hybridization (while there might be some based on older literature). We adjusted the discussion accordingly.**

Fig 1. Pca203: bW26HD1? (delete 6?); Pca12SD80 should be bW6-HD1?? It’s confusing. Please, call haplotype allele numbers consistently. Check all!

**Author’s Response: We addressed this issue in the updated manuscript and check for consistency with previous publications and throughout the manuscript.**

Fig 2. Wrt incongruency and inconsistency in a phylogenetic tree when analyzing STE3.2-2 and STE3.2-3 together; in several analyses in other studies, the pheromone receptor sequences were C-terminally truncated to exclude the cytoplasmic tail to optimize the alignment and allow various PRA alleles to be directly compared (e.g., Coelho et al. 2017, Cuomo et al. 2017; Bakkeren et al., 2008,FGB 45,S15). No detail is given on how the pile-ups and analyses were done.

**Author’s Response: We replaced the maximum likelihood tree with Bayesian tree for Fig 3, now *STE3.2-2* and *STE3.2-3* are being directly compared. We updated the results on Pages 13-14 accordingly. We improved the method section and included how alignments are generated and model used for inferring Bayesian trees. We provide all alignments with our Dryad repository for this manuscript.**

Legend Fig S10: ‘yellow boxes indicate PR loci’; I do not see yellow boxes….?

**Author’s Response: We updated all mummer plots, *PR* loci are now indicated in red boxes in S13 Fig.**

Reviewer #2: Luo and colleagues offer a genome-level analysis of mating type (MAT) loci in four economically important cereal rust species. The study reveals that these species possess a tetrapolar mating system characterized by unlinked biallelic pheromone/receptor (PR) locus and a multiallelic homeodomain transcription factor (HD) locus. Through a range of genomic analyses, the authors report signs of recombination suppression and genomic degeneration around the PR locus. Conversely, the HD locus appears to be more conserved and does not show signs of genetic degeneration, suggesting ongoing recombination in its neighboring regions as normally observed in other tetrapolar basidiomycetes. The work also emphasizes the potential role of these MAT loci in promoting nuclear pairing during the asexual cycle, offering insights into the evolution of virulence in these critical pathogens. Overall, the paper has the potential to significantly advance our understanding of mating compatibility mechanisms in this important group of fungi with possible repercussions for agriculture and disease management.

While the manuscript provides valuable insights into mating type loci in rust fungi, it needs several revisions. First, the language needs polishing for clarity and readability, and typographical errors and inconsistent formatting should be fixed. Second, the figures need a thorough review to improve readability and presentation. Finally, the Methods section is too vague, making it hard to understand the research process and raising concerns about reproducibility. These general issues should be addressed alongside my more specific comments/suggestions below.
**Author’s Response: We thank the reviewer for the critical suggestions and careful evaluation. We now have update the language, form and content of the whole manuscript. We extended the methods section, deposited associated raw data on Dryad (https://datadryad.org/stash/share/XCyaQ2j8q8yI28amg3ct1WplAOlh-yTUHfaz_jyDYK4), and provide all analytic code of the manuscript on Github (https://github.com/ZhenyanLuo/codes-used-for-mating-type). We judge the manuscript significantly improved thanks to the reviewers input.**

- Major points -

Line 83: It appears that the manuscript uses reversed nomenclature for the bW (bWest) and bE (bEast) genes, potentially following the precedent set in a previous paper on rust fungi mating type loci (Cuomo et al. 2017, G3, 7:361-376). However, based on foundational research on mating type loci in smut fungi (Ustilaginomycotina), the bW gene corresponds to HD2 and bE to HD1. Therefore, I recommend revising this information for the sake of accuracy and ensuring that this corrected nomenclature is consistently applied throughout the manuscript, so that it can be adopted and reflected in future studies.

**Author’s Response: We thank the reviewer for pointing this out. Although we agree that this is an important consideration, we decided to keep using the gene name as Cuomo et al. 2017, since we used their gene models as initial queries for starting this study. This is consistent with another recent study on *HD* alleles in cereal rust fungi presented by Holden et al. 2023 using the same naming method. To clarify this point, we added a sentence on page 5 for pointing out the specific naming convention used in our manuscript.**

Lines 163-164: The manuscript establishes that the HD and PR loci are located on separate chromosomes, thereby supporting a tetrapolar MAT organization. Given the significance of this finding to the overall narrative of the study, it would be beneficial to emphasize this information through visual representation in a main figure. Specifically, I recommend the addition of a figure (potentially as Fig. 1) that provides a simplified version of what is currently shown in S1 Fig. This figure could graphically depict the chromosomes containing the PR and HD loci for each of the four studied species. To provide additional evolutionary context, this could be juxtaposed with the species phylogeny that is currently presented in S4 Fig. Merging these key elements into a main figure would enhance the comparative aspects of the study and facilitate a more immediate understanding of the findings.

**Author’s Response: We updated Fig1, combining the species tree and the gene tree of *HD* alleles. We decided to leave S4 Fig as a separate figure to make it easier to follow by not overloading figure 1.**

Lines 205-207: The authors present interesting findings regarding the genomic organization of pheromone receptor and pheromone genes across the Puccinia species examined. Specifically, they snote that STE3.2-2 is found adjacent to MFA2, while MFA1 is located at varying distances from STE3.2-3. Additionally, in Pt, two identical MFA1 genes were identified, possibly indicative of a recent duplication event. On the other hand, in Pgt, two distinct pheromone genes (MFA1 and MFA3) were found in association with STE3.2-3. These observations raise several questions: (1) Why were the two identical genes in Pt labeled as MFA1/3? Is this a nomenclatural choice or does it imply something about the gene function? (2) Given that MFA3 is unique to Pgt, it could be that this gene product may not function in mating type recognition, or might not even be a mating pheromone? (3) Could the authors speculate on what might constitute the protein sequence of the mature, active form of the pheromone? (4) How does this compare with other Pucciniales previously sequenced (e.g., Melampsora larici-populina).

**Author’s Response: We followed the naming convention for the two near identical copies of Ptmfa1/3 as originally presented by Cuomo et al. 2017 which named these genes *Ptmfa1=3* and *Ptmfa3=1*.**

**We now significantly extended the discussion around *PGTMFA1* and *PGTMFA3* found encoded at the *STE3.2-3* locus. Based on previous publications, this could be also an indication that the *PR locus* is not strictly biallelic in *P. graminis* f. sp. *tritici*, yes it is also possible to be a gene product, which is not related to mating.**

**We added a brief discussion that the predicted mature *PGTMFA1* and *PGTMFA3* peptides are sequence diverse and might have different receptor specificities.**

**Cuomo et al. 2017 reported that *Melampsora larici-populina* had more than two potential *Pra* genes (not considering *STE3.2-1* as functional *Pra* gene).**

Lines 234-241: The authors suggest that the differing branching patterns of HD1 and HD2 within each species may indicate recombination between these genes. This could potentially lead to self-compatible HD1-HD2 allele pairs. However, an alternative explanation could be a lack of strong phylogenetic signal, as evidenced by some low bootstrap values. I recommend a more cautious interpretation of these results. Additionally, a phylogenetic analysis using protein sequences might be informative. It's important to see if the nucleotide differences result in functionally distinct proteins. Also, in many other basidiomycetes, the N-terminal regions of HD1 and HD2 are more variable, possibly affecting self/non-self-recognition. Have you explored this in the available HD alleles?

**Author’s Response: We thank the reviewer for pointing out this issue. We replaced the maximum likelihood tree with Bayesian tree, which provides better support values. We also ran AU-test for testing whether topologies of *bW-HD1*** **gene tree are consistent with *bE-HD2* on page 12-13. We discussed the obtain results more carefully. Yes our result also suggesting the N-terminal regions of *bW-HD1* and *bE-HD2* alleles are more variable as shown in the new S21 Fig.**

Lines 263-264: The conclusion in this section suggests that the incongruent topologies of STE3.2-2 and STE3.2-3 indicate long-term recombination suppression around the PR locus. However, the actual evidence supporting long-term recombination suppression would be phylogenetic clustering of alleles by mating type across species, rather than clustering by species (i.e., trans-specific polymorphism). This is because the two alleles ceased recombining long ago and have subsequently accumulated substitutions independently. It is also worth noting that the incongruent topologies between the two trees are associated with relatively low bootstrap values in the STE3.2-2 tree. Again, these low bootstrap values may affect the confidence level of the inferred phylogenetic relationships, potentially complicating the interpretation.

**Author’s Response: We replaced the maximum likelihood tree with Bayesian tree for Fig 3, result has been updated on pages 13-14 accordingly. We also combined all *Pra* alleles into a single tree to clearly highlight trans-specific polymorphisms. We updated the results section accordingly.**

Lines 450-478: This study provides important data on mating type gene expression. Since STE3.2-1 doesn't appear to be mating type-specific based on your results, it might be useful to examine if this gene is upregulated under the conditions you studied, or how its expression varies during infection or spore formation. This could give more context on a possible role of this gene. Additionally, you might consider referencing similar genes in other basidiomycetes that don't directly affect mating type. For instance, in Cryptococcus deneoformans, the Cpr2 gene competes with the Ste3 receptor and triggers unisexual reproduction when overexpressed. Similarly, in some mushroom species, receptor-like genes near the PR locus are linked to vegetative growth, not mating type (e.g., Wirth S, et al. 2021). This could hint at broader functions for genes like STE3.2-1 in rust fungi.
**Author’s Response: The expression of *STE3.2-1* was beyond the detection limit in all analysed datasets. This is consistent with previous observations of Cuomo et al. 2017. We included this in the discussion and provided references to similar observations in other fungi as suggested.**

- Minor points -

Line 43: The term “di-om mating” appears in the abstract but is not explained or mentioned elsewhere in the manuscript. The terminology is not only unclear but might also be too technical for a broader readership at the abstract level. Did you intend to write “di-mon mating”?

**Author’s Response: We have addressed this in the updated manuscript. We removed di-mon mating.**

Lines 62-65: The sentence “Genetic degeneration footprints are higher rates of (non)synonymous substitutions (dN, dS), accumulation of transposable elements, reduced gene expression and reduced gene numbers which are all a consequence of recombination cessation” could benefit from clarification. Are all these “footprints” of genetic degeneration always observed in non-recombining regions?

**Author’s Response: We have addressed this in the updated manuscript and clarified that not all have to appear at the same time.**

Line 72: Please consider changing “100s” to “hundreds”.

**Author’s Response: We replaced “100s” to “hundreds” on page 4 based on your suggestion.**

Line 74: According to standard taxonomic nomenclature, the abbreviation “sp.” in Microbotryum sp. should not be italicized. Furthermore, if referring to multiple species within the Microbotryum genus, the correct form should be “Microbotryum spp.” Please verify this and other instances throughout the text for consistency, such as on lines 101 and 501.

**Author’s Response: We have addressed this in the updated manuscript.**

Line 77: The phrase “In most cases, two MAT loci control mating” could be more accurately stated as, “In most cases, two MAT loci determine mating type identity.”
**Author’s Response: We have addressed this in the updated manuscript on page 4 by using ‘determine’ rather than ‘control’.**

Lines 78-79: To enhance clarity and consistency in the manuscript, consider standardizing the nomenclature for the pheromone receptor genes, choosing either “PRA” or “STE3” for uniform reference throughout. Additionally, please take the opportunity to review and standardize the formatting of gene names (italics vs. non-italics, uppercase vs. lowercase) in both the main text and figure legends, such as in S2 Fig.
**Author’s Response: We have added more information to describe the relationship between *Pra,* STE3, *STE3.2-2* and *STE3.2-3* on page 7. We decided to use *Pra* when we refer to mating type receptor genes and *STE3.2-2/STE3.2-3* when we refer to specific alleles thereof. We also addressed the format of gene/protein names issue carefully.**

Line 81: The term “PR haplotype” is used, which may not align well with conventional nomenclature for individual variations of a gene or locus, especially in the context of mating type loci literature. To ensure clarity and consistency, consider using the term “PR allele” or “idiomorph” instead. Furthermore, when discussing STE3.2-2 and STE3.2-3, it might be more appropriate to refer to these as two different alleles of the same gene. This would align better with terminology used in other parts of the manuscript, such as lines 252-253, and contribute to a more cohesive narrative."

**Author’s Response: We agree with the reviewer and updated the manuscript accordingly.**

Line 84: It is important to clarify that the products of HD1 and HD2 from the same allele do not form heterodimers. Heterodimerization occurs exclusively between HD1 and HD2 products that are derived from different alleles. Consequently, these products must differ in compatible mating partners.
**Author’s Response: We have added more information on page 5 to clarify this issue.**

Line 90: The term “Biopolar” should be corrected to “Bipolar,” and the phrase “are advantages in” should be revised to “are advantageous in.” Similar corrections are needed on line 93.

**Author’s Response: We have corrected the manuscript accordingly.**

Line 91: The phrase “that undergo selfings” should be corrected to “that undergo selfing.”

**Author’s Response: We have corrected ‘selfing’ on page 5 accordingly.**

Lines 93-95: The sentence, “In outcrossing populations it is advantages that MAT loci are multiallelic because this increases the success of compatible gametes (in doing what?) derived from two independent individuals,” needs revision for clarity and completeness.

**Author’s Response: We have rewritten this sentence in page 5-6 for clarity.**

Lines 93-95: The sentence regarding the advantages of multiallelic MAT loci in outcrossing populations is currently unclear and incomplete. Specifically, it would be helpful to clarify what is meant by “increases the success of compatible gametes.” Are you referring to a higher likelihood of successful fertilization, greater genetic diversity, or some other form of “success”?
**Author’s Response: We have rewritten this part to better explain ‘increase the success of compatible gametes’ on page 5.**

Lines 96-97: The segment “with ten to hundred known and estimated haplotypes” is unclear and needs clarification.
**Author’s Response: We updated the manuscript for clarity and removed this sentence.**

Line 98: While the term “plesiomorphic” is technically accurate, it might come across as overly technical and potentially less accessible to a broader audience. In this case, it might be beneficial to use the term “ancestral” as a replacement to enhance readability.

**Author’s Response: We agree. We have replaced “plesiomorphic” with “ancestral” to enhance readability.**

Lines 100-103: To offer more background and improve clarity, consider rewording this section along the lines of: “For example, Microbotryum spp. are highly selfing, and multiple independent events have linked the HD locus and the PR locus into large non-recombining regions. These regions differ in both their size and age and have been formed by successive steps of recombination suppression, resulting in several adjacent “evolutionary strata” of differentiation between non-recombination chromosomes.
**Author’s Response: We have rewritten this sentence on page 6. We added information of evolutionary strata on page 6.**

Line 112-113: You may want to rephrase the sentence to: “as sexual reproduction is prevented by management strategies that remove the secondary host (e.g., Berberis) required for sexual reproduction.”

**Author’s Response: We have rewritten this sentence on page 6.**

Lines 116-117: To better articulate the point, consider revising the sentence to: “The asexual epidemic potential of a given rust isolate is largely determined by the complement of effector genes encoded in its two nuclei, as well as by the resistance (R) genes present in crop varieties grown in a specific region, which confer them the ability to recognize and mount defenses against specific pathogens or their effectors”.
**Author’s Response: We have rewritten this sentence on page 6-7.**

Lines 146: “loci” should not be italicized.
**Author’s Response: We fixed this mistake in this manuscript.**

Line 158: There seems to be a discrepancy in the notation for “Pgt 210.” Is this the same as “Ptg 21-0”? If so, please consider standardizing.

**Author’s Response: Thank you for pointing out, we have corrected all *Pgt 210* to *Pgt 21-0*.**

Line 184: For enhanced clarity, consider rewording the sentence along the lines of: “We employed a phylogenetic approach to trace the inheritance of PRA and HD genes in each haplotype. Under a tetrapolar system, compatible haplotypes in a dikaryotic genotype are expected to have one copy of either STE3.2-2 or STE3.2-3 and exhibit different alleles at the HD locus.”
**Author’s Response: We updated the manuscript for clarity.**

Lines 207-208: To conform with standard notation, please separate the numbers from the units in the manuscript. For example, change “0.54Mb” to “0.54 Mb” and “10kb” to “10 kb,” and apply this consistently throughout the text.
**Author’s Response: We have corrected this throughout the text and used mbp/kbp for consistency.**

Line 222: For the mention of trans-specific polymorphism, consider adding a relevant citation to provide further context and support for this concept.
**Author’s Response: We updated the manuscript accordingly. We extended the introduction on trans-specific polymorphism and added relevant citations.**

Line 308: The use of the term “sister chromosomes” could introduce ambiguity. I recommend using the term “homologous chromosomes” instead. Additionally, it would be insightful if the manuscript could also address the extent of synteny conservation among the MAT chromosomes across the species studied. Providing information on the average level of divergence between the analyzed haplotypes in different species would also be beneficial.

**Author’s Response: We decided to stick with the term “sister chromosomes” because homologous chromosomes was too unspecific. We deem the whole chromosome comparisons of *MAT* loci carrying chromosomes between species out of scope of our current manuscript which is extensive enough already. We will include this in follow up publications.**

Line 314-318: The phrase “user features” in this sentence is unclear and might cause confusion. Could you please elaborate on what you mean by this term or consider using a more straightforward expression? Also, it is mentioned that no “obviously aberrant patterns of gene or transposon density” were observed around the HD locus. Could you specify whether there is a particular analysis that supports this assertion? Lastly, I believe the methods used to predict the centromere locations are not very well discussed – if these were determined based on previous research, please provide the appropriate citations.
**Author’s Response: We updated the manuscript accordingly and improved clarity. We also extended the methods section to cover centromere predictions more explicitly.**

Lines 386-387: It is noteworthy that in Pca and Pst, there is a marked drop in gene density on only one side of the pheromone receptor gene locus. Could this asymmetry be directly correlated with the position of the pheromone genes that is distinct between the two PR alleles/idiomorphs?
**Author’s Response: This is an interesting observation and suggestion. We have not followed up in the current version of this manuscript. We discuss the extension on TE rich region and the Pra one locations in more detail in the discussion.**

Line 423: It is stated that “Pca showed the highest level of variation of HD loci at the species level (S14 Fig).” However, upon reviewing S14 Fig, I noticed that this figure only displays HD1-HD2 allele variation for three of the species (Pst, Pgt, and Pt). Therefore, the data supporting this claim for Pca appears to be missing or not clearly presented in the supplemental figure. Could you please clarify or correct this?”
**Author’s Response: We now extensively updated the analysis of *HD* alleles in all four species. We developed a novel *de novo* reconstruction approach for *HD* alleles directly from Illumina short-read datasets. We validated this method using *Pca 203* short-read data and compared the reconstructed alleles against the reference (S21 Figs). The *de novo* reconstruction of *HD* alleles showed that *HD* loci are multiallelic in cereal rust fungi. We identified twelve distinct HD alleles for *Pca*.**

Line 439: A higher number of SNPs in the STE3.2-2 and STE3.2-3 genes was found in Pgt compared to other species. Could this increase be attributed to the Pgt isolates analyzed be more divergent? It might be helpful to expand on this by comparing SNP frequencies in other genomic regions as well. For instance, S16 Fig. suggests that STE3.2-1, which is not directly related to mating type determination based on your findings, also seems to exhibit a high level of variation. This could indicate that the elevated SNP levels in STE3.2-2 and STE3.2-3 may not be gene-specific but rather a broader characteristic of the Pgt isolates examined? If so, this might challenge the suggestion made in Discussion section (lines 489-490) that “Pgt might be multiallelic at the PR locus.”

**Author’s Response: We now extended the analysis and discussion of the *PR* locus in *Pgt* significantly. This discusses the potential of *Pgt* not being biallelic more carefully and calls for more phased chromosome scale genomes at the population level for cereal rust fungi. Performing genome wide SNP analysis for all four cereal rust fungi is out of this scope of the current manuscript.**

Lines 503-507: About the role of STE3.2-1, I find it important that it should be mentioned that other non-mating type pheromone-like receptor genes have been found in other basidiomycetes, including the Cpr2, which is known in Cryptococcus deneoformans to compete with the Ste3 receptor for signaling and whose overexpression elicits unisexual reproduction (Hsueh et al., 2009, EMBO J. 28:1220-1233). In mushroom species, pheromone receptor-like genes are also found close to the PR locus, but these do not determine mating type identity. A recent report suggest they may have a role in vegetative growth, and self-signaling (e.g., see Wirth S, et al. 2021, J Fungi, 7:399), thus revealing an important role for hyphal growth and development.

**Author’s Response: We included this in the discussion and provided references to similar observations in other fungi as suggested.**

Lines 526-528: While it is appropriately highlighted the presence of trans-specific polymorphism in the STE3 alleles of rust fungi, it might be beneficial to the discussion to also note that STE3.1 and STE3.2 alleles in other Pucciniomycotina species such as Microbotryum, as well as in red yeasts and Leucosporidiales (for further context, see Coelho et al. 2010, PLoS Genetics 6:e1001052 and Maia et al., 2015, Genetics 201:75-89), appear to be evolutionarily older than those specific to the Pucciniales. This contextual information could provide additional depth to your discussion on the evolutionary aspects of these alleles.

**Author’s Response: We addressed these suggestions in the updated manuscript and shifted the focus away from trans-specific polymorphism for *Pra* genes.**

Line 539: Typo in the term “Pucciniomycotina”
**Author’s Response: We fixed this in the updated manuscript.**

Lines 600-601: As it reads, the statement "There is no evidence of recombination suppression at HD loci, as there are no trans-specific polymorphisms between species and support for macro-synteny among species" appears to be contradictory or unclear.
**Author’s Response: We fixed this in the updated manuscript.**

Line 625: As stated above, the Method section requires careful revision and added details. For example, in the “Phylogenetic analysis of the HD and PRA genes” subsection, the absence of parameters used in OrthoFinder for orthology inference and in IQ-TREE2 for phylogenetic analysis is a notable omission.
**Author’s Response: We have addressed this issue and updated. We significantly extended the methods section, provide a Github and Dryad repository.**

Line 637: Should be Branco et al.
**Author’s Response: We have addressed this in the updated manuscript.**

Fig 3, Line 329: The phrase "10k bp sized windows" should be amended to "10 kb-sized windows". Moreover, the function of the shaded black/grey areas below the dS plots is unclear. If these are intended to represent gene-containing regions, it would be helpful to make this explicit in the figure legend or main text. I also noticed that not all shaded areas have corresponding dS values. If this discrepancy exists because an allele is missing in one of the two haplotypes, please clarify that point. My concerns extend to the calculation of dS values, as mentioned in my comment for S8 Fig below. Additionally, to improve visual distinction between the tracks, consider applying different color schemes for Transposable Elements (TEs) and genes. Lastly, as a general comment for all figures in the manuscript, maintaining a consistent color code for labeling different species would enhance readability and interpretation, as observed in Figures 1 and 2.

**Author’s Response: We have addressed this issue and updated in the revised manuscript.**

Fig 4: The gene tracks are rather small, making it difficult distinguishing individual genes. To enhance readability, I suggest enlarging the gene tracks.
**Author’s Response: We fixed this Fig 4 and other microsynteny plots with larger gene tracks.**

S2 Fig. Line 36: Please correct the phrase “cross species” to “across species” for clarity.
**Author’s Response: We fixed the figure caption based on the suggestion.**

S3 Fig. Line 47: The word “there” appears to be a typographical error and should be corrected to “their.”
**Author’s Response: We fixed this error.**

S4 Fig: To aid in cross-referencing between the text and the figure, consider adding the species abbreviations next to each species name in the phylogenetic tree.

**Author’s Response: We have addressed this in the updated manuscript by adding species abbreviations and corresponding species name in the figure caption.**

S5 Fig: I suggest that the phylogenetic tree be rooted at the midpoint. This would prevent inadvertently implying a directional bias in the tree when using the MlpPh1 gene as the outgroup.
**Author’s Response: We updated S4 Fig, since using *MlpPh1* gene or *MlpPh2* gene as outgroup can result in a directional bias, we constructed the protein tree without outgroup and rooting at the midpoint as suggested.**

S8 Fig: The figure includes calculated dS values for the STE3.2-2 and STE3.2-3 genes (also plotted in Fig 5). Given that these two genes are not easily alignable, it would be helpful to clarify the methodology used to calculate these dS values. Additionally, for enhanced transparency, consider providing the percentage of pairs of genes for which dS was possible to be calculated in each chromosome across all studied species and/or include the associated raw data of these dS

calculations.

**Author’s Response: We have rewritten how we done this analysis in the method section. This includes pairing of orthologs, aligning and calculate d_s_ values. We deposited the code on Github. We also provide raw data of d_s_ values of gene pairs in Dryad.**

References Section: I noticed several inconsistencies in the formatting of the references, particularly concerning the use of italics. For example, references 33 and 59 do not follow the same formatting as others in the list. Please carefully revise the entire section.

**Author’s Response: We fixed this carefully.**

Reviewer #3: In this study the author investigated the structure, evolution and function of mating type loci in several cereal rust fungi using publicly available dataset of genomes and expression data analysis.
Very little is known on the evolution of mating type loci in rusts compared to other Basidiomycota and their study can be highly relevant to better understand sexual and asexual cycle of these damaging pathogens.
The study goal is very interesting and I didn’t detect any major methodological flaws. However, I found overall that the state of art was not enough described, some part of the material and method were not enough detailed and some results need to be better discussed in their context.
**Author’s Response: We thank the reviewer for the critical suggestions and careful evaluation. We now have update the language, form and content of the whole manuscript. We extended the methods section, deposited associated raw data on Dryad (https://datadryad.org/stash/share/XCyaQ2j8q8yI28amg3ct1WplAOlh-yTUHfaz_jyDYK4), and provide all analytic code of the manuscript on Github (https://github.com/ZhenyanLuo/codes-used-for-mating-type). We judge the manuscript significantly improved thanks to the reviewers input.**

Major comments:
-For example, recombination suppression around the PR locus was shown previously to be likely ancestral to Basidiomycota (see Devier et al 2008 Genetics); this doesn’t appear clearly in neither the abstract, the introduction and the discussion of the results.

**Author’s Response: We now updated the manuscript and extended the introduction including the provided reference.**

-The estimated size of the region with recombination suppression found around PR locus is not clearly described; how much this region varied in size between species ? between haplotypes?

**Author’s Response: We now updated the manuscript accordingly and provide estimates in the discussion.**

-The gene tree for the PR locus should include both haplotypes to conclude to transpecific polymorphism (Fig2). Transpecific polymorphism is clear from mfa genes however.

**Author’s Response: We have fixed the gene tree of *Pra* genes in Fig 2, *STE3.2-2* and *STE3.2-3* are now in the same tree.**

-It is to note that there seems to have some haplotype clustering within species for bW-HD1 gene both for Pt and Pst species. This may suggest some recent recombination suppression (Fig1), which is consistent with high dS value for this gene.

**Author’s Response: We now included statistical AU test to compare tree topologies of *bW-HD1* and *bE-HD2* within each species. We discuss these results in the context of recombination or not at this locus.**

-Looking at the allelic diversity within species, I wonder if the authors consider retrieving the PR/HD sequences by blast analysis in de novo assemblies; read mapping in a region rich in TE as in the PR region may be challenging. The method of estimation of the number of alleles at HD and PR locus need to be further described

**Author’s Response: These are excellent suggestions. We now developed a novel *de novo* reconstruction approach for HD alleles directly from Illumina short-read datasets (see methods section for details). We validated this approach with *Pca 203* and reconstructed between six to twelve *HD* alleles for each species. We also provide S17-25 Figs to show Illumina short-read mapping to *MAT* loci. Both *STE3.2-2* and *STE3.2-3* have good mapping coverage with high mapping qualities (>40) in all four species.**

-the finding that the MAT genes are expressed late in the asexual infection cycle during spore production is interesting ; however, it is likely not the only gene to be overexpressed in this stage; more evidence / functional analysis need to be done to check that MAT loci govern compatibility of nuclei in somatic hybridization;

**Author’s Response: We now added additional analysis to confirm the expression of MAT genes late during the infection cycle. We also clarified in the discussion their potential role in nuclear pairing and somatic hybridization. We rephrase our observations more carefully. Functional analysis, e.g. mutants, is well out of the scope of this manuscript.**

L576-582: conclusions need to be alleviated
-the evolutionary drivers of recombination suppressions are presented in the introduction, but the finding of recombination suppression around the PR loci is not discussed in this context in the discussion.

**Author’s Response: We have addressed this in the updated manuscript. We significantly improved the logic flow and discussion of the manuscript. We also removed the “Conclusion” section as it did not add substantially to the manuscript.**

Minor comments:
Introduction
-l60-68: other models predict recombination suppression around mating-type loci and could be presented here

**Author’s Response: We have addressed this in the updated manuscript.**

-l75: more Basidiomycota examples could be quoted as recombination suppression was described in some species of Cryptococcus, Agaricus, Ustilago… It could be interesting to describe if recombination suppression was associated to bipolarity/tetrapolarity, mating system, number of alleles at mating-type loci in these systems.

**Author’s Response: This is an excellent suggestion and we rephrased the manuscript accordingly.**

-l93: typo for “advantages”

**Author’s Response: We have corrected this issue on Page 5.**

-l101: the recurrent comparison with Microbotryum should be justified with a phylogenetic context that may not be obvious to readers; furthermore evolution of recombination suppression in this genus should be better descrivzs: if it is in bipolar or tetrapolar species, around HD or PR genes or linking them, etc…

**Author’s Response: We have addressed this in the updated manuscript.**

-l105-106: this sentence is not related to the previous paragraph and the main goal of the study in my opinion

**Author’s Response: We have addressed this in the updated manuscript. We completely restructured the flow of the whole manuscript.**

-l143-145: More context need to be given to understand why the author have the presented
hypothesrs ; I failed to understand why the authors tested these hypotheses in particular when reading the introduction.

**Author’s Response: We now have rewritten this part on page 8-9. The new logic flow clearly supports the new objectives.**

Results
- Table 1: statistics about the genome assemblies (N90, L50 …) seems lacking and should be presented

**Author’s Response: We added BUSCO values for showing the completeness of genome assemblies in new Table 1.**

Author mention “chromosome scale” assemblies, are telomere and centromere well identified? Is it haplotype-phased assemblies? Are all genomes dikaryotic genotypes?

**Author’s Response: These genomes are published in other studies, we labelled haplotype-phased assemblies with star symbol in Table 1.**

-l184: “phylogenetic hypothesis” is not clear

**Author’s Response: We have rewritten this in the updated manuscript.**

-l345: previous sentences state the opposite

**Author’s Response: We have addressed this in the updated manuscript.**

-l393: a distance of 473 kb to the centromere may be high for physical linkage; are other elements supporting linkage to the centromere such as segregation analyses?

**Author’s Response: We tried to perform segregation analysis for supporting linkage to the centromere before, but there is only a limited set of *Pst* isolates available that are suitable for such kind of an analysis as most isolates are from asexual clonal lineages.**

Material & method
-l621: the mapping approach needs to be futher described; how was the sequence retrieved after mapping?

**Author’s Response: We now have updated the methods section completely including an associated Github and Dryad repository.**

-l623: the search of orthologs with GeniousPrime needs to be further described; which dataset was used?

**Author’s Response: We fixed our method section based on the provided suggestions. *Mfa* genes were identified by using *mfas* *of Pt BBBD*, *Pgt SCCL* and *Pst 78* studied by Cuomo et al.2017 as a custom motif database to search similar pattern in all studied species with Geneious Prime v. 2022.1.1 (88), using a function built from fuzznuc.**

-l626-627: which dataset was used to build the rooted species tree? Is it coding sequence?

**Author’s Response: We have now update the methods section accordingly. To construct a species tree for cereal rust fungi, one protein sequences of one nuclear genome for each cereal rust fungus species was used as input data. The data was processed by Orthofinder 2.5.5 to infer maximum likelihood trees from multiple sequence alignments (MSA). Fasttree v2.1 was used to calculate likelihood-based local support values. We used single-copy orthologous proteins identified by Orthofinder for constructing the species tree with fasttree.**

-L637: typo “Branco et al”

**Author’s Response: We have fixed this mistake in the method session.**

-One separate section could summarize all strains/genomic data used in this study

**Author’s Response: We now have Tables 1-2 and S1 Tables for summarizing data used in this study.**

-the RNAseq dataset used (time points, number of replicates by time points) need to be better described

**Author’s Response: We now have S2 Table to describe the RNA dataset we used in this study.**

-TE annotation and gene models used need further description

**Author’s Response: We now updated the method section. We provide more extension description of the performed TE annotation including all code in the associated Github repository. All gene annotations have been published previously and are described accordingly in the manuscript and included tables.**
